# Reducing the Use of Pesticides with Site-Specific Application: The Chemical Control of *Rhizoctonia solani* as a Case of Study for the Management of Soil-Borne Diseases

**DOI:** 10.1371/journal.pone.0163221

**Published:** 2016-09-26

**Authors:** Ronan Le Cointe, Thomas E. Simon, Patrick Delarue, Maxime Hervé, Melen Leclerc, Sylvain Poggi

**Affiliations:** INRA UMR1349 IGEPP, 35653, Le Rheu, France; Universita degli Studi di Pisa, ITALY

## Abstract

Reducing our reliance on pesticides is an essential step towards the sustainability of agricultural production. One approach involves the rational use of pesticides combined with innovative crop management. Most control strategies currently focus on the temporal aspect of epidemics, e.g. determining the optimal date for spraying, regardless of the spatial mechanics and ecology of disease spread. Designing innovative pest management strategies incorporating the spatial aspect of epidemics involves thorough knowledge on how disease control affects the life-history traits of the pathogen. In this study, using *Rhizoctonia solani/Raphanus sativus* as an example of a soil-borne pathosystem, we investigated the effects of a chemical control currently used by growers, Monceren^®^ L, on key epidemiological components (saprotrophic spread and infectivity). We tested the potential “shield effect” of Monceren^®^ L on pathogenic spread in a site-specific application context, i.e. the efficiency of this chemical to contain the spread of the fungus from an infected host when application is spatially localized, in our case, a strip placed between the infected host and a recipient bait. Our results showed that Monceren^®^ L mainly inhibits the saprotrophic spread of the fungus in soil and may prevent the fungus from reaching its host plant. However, perhaps surprisingly we did not detect any significant effect of the fungicide on the pathogen infectivity. Finally, highly localized application of the fungicide—a narrow strip of soil (12.5 mm wide) sprayed with Monceren^®^ L—significantly decreased local transmission of the pathogen, suggesting lowered risk of occurrence of invasive epidemics. Our results highlight that detailed knowledge on epidemiological processes could contribute to the design of innovative management strategies based on precision agriculture tools to improve the efficacy of disease control and reduce pesticide use.

## Introduction

The sustainability of agricultural production requires ecological management practices and, among others, parsimonious use of chemical-based control strategies that should be based on thorough understanding of epidemiological processes [1,2]. The sustainable use of pesticides in cropping systems is an important issue and the development and the adoption of well-reasoned practices for controlling crop pests and diseases depend on technical and sociological issues [3–5]. Institutional indicators, such as the treatment frequency indicator (TFI) have been defined to monitor pesticide use in fields [6,7]. TFI measures the number of "approved doses" sprayed on the whole surface of a plot during a cropping season. This definition highlights three possibilities for reducing the amount of pesticides used: (1) reducing the number of spray applications during the cropping period, (2) decreasing the applied dose (compared to the reference dose), and (3) restricting the treated area.

The frequency of spraying can be reduced through the use of decision-making tools [8]. These tools usually rely on predictive models and recommend treating crops at the right time only if necessary and with the most appropriate chemical product. Applying a lower dose is often carried out in cereal production systems for economic reasons, but this practice is controversial with regard to fungicide resistance. On the one hand, the Fungicide Resistance Action Committee [9] recommends limiting the number of applications, using the full recommended dose and combining fungicides with different modes of action. This advice is based on the hypothesis that using lower doses may potentially promote pesticide resistance [10,11]. On the other hand, others argue that lower doses reduce the selection pressure favouring resistant strains and therefore improve fungicide durability [12–14]. The third pesticide reduction strategy is to restrict the treated area by spatially localizing applications. This method called “site-specific application” allows, through precision farming, to target a specific area accurately and spare the rest of the crop [15–18].

Site-specific applications can be implemented as either preventive or curative control strategies. Regarding preventive control strategies, current site-specific application consists in adjusting the dose across the field surface according to canopy density. With this strategy, denser canopies receive a higher dose of fungicide, assuming that a dense canopy creates a microclimate more conducive to the disease, and contributes significantly to crop yield [19,20]. For curative fungicides, site-specific application is sometimes practised by farmers in fields, e.g. by using a knapsack sprayer on patches of diseased plants. The difficulty lies in accurately differentiating the targeted symptoms from those of other diseases and from abiotic stress [21,22]. In addition, fungal disease symptoms must be detected early enough in the cropping season; for example, roots infections caused by soil-borne pathogens such as *Rhizoctonia solani* Kühn on sugar beet can result in detectable symptoms only late in the cropping season on above-ground plant parts (wilting) [23,24], thereby preventing early fungicide treatment. Designing spatial management strategies using the site-specific application method therefore requires good knowledge of epidemiologic processes.

In the case of invasive fungi, and particularly for soil-borne pathogens, a significant body of literature deals with criteria for invasion and persistence of diseases (e.g. [25,26]). These studies demonstrate that epidemics at the population scale occur only if the probability of spread between individuals lies above a threshold probability [27,28]. In particular, percolation theory predicts that if a certain fraction of sites is unavailable for colonization, invasion stops. Previous studies combining epidemiology and percolation theory have demonstrated that: (i) epidemic dynamics can change dramatically (switching from invasive to non-invasive patterns) according to a spatial threshold [29], and (ii) a threshold proportion of protected sites may shield the whole population [30,31]. In an agronomic context, such experimentally tested findings suggest that controlling the spread of locally-spreading pathogens is possible through local application of treatments that decrease the transmission of the pathogen between sites.

Plant disease epidemics can be broken down into two epidemiological processes: (i) the spatial spread of the pathogen (mycelium or spore) within the environment (soil or air) and (ii) the infectivity of the pathogen that describes its ability to actually infect its host when they are in contact. Biological and chemical treatments can affect these epidemiological processes through various biological and toxicokinetic-toxicodynamic processes and their effects may change with the genetic background of both the host and the pathogen, and environmental conditions. Thus, good knowledge about the effects of treatments is central for designing new management strategies, for instance strategies based on localized control, that would contribute to the reduction of pesticide.

In this study, we considered the effects of the commercial fungicide Monceren^®^ L on the life-history traits of *R*. *solani*, a soil-borne pathogen that causes substantial loss on various crops worldwide. Following previous studies that have significantly contributed to the understanding of soil-borne disease epidemics [32–34], we used *R*. *solani/Raphanus sativus* as a model of soil-borne pathosystem in vegetable crop and combined placement experiments, statistical analyses and modelling to assess the effects of this fungicide on both the saprotrophic spread of the pathogen from inoculum-donors (i.e. a mycelium) and its infectivity once it reaches the immediate vicinity of its host. We finally tested the potential localized “shield effect” of Monceren^®^ L on pathogenic spread in a site-specific application context, i.e. the efficiency of this chemical to contain the spread of the fungus from an infected host when the application is highly localized, in our case, sprayed on a narrow strip of soil between the infected host and a recipient bait.

To assess the effect of the fungicide on mycelial spread, we introduced the individual-level concept of saprozone, directly derived from the concept of pathozone defined by Gilligan [35] which has been central to the study of soil-borne disease epidemics. The pathozone is a host-centred form of a dispersal kernel which is described by a surface indicating the change in the probability of successful infection P(*x*,*t*) of a recipient-host by an inoculum-donor located at distance *x* after a time of exposition *t*. Unlike the pathozone concept, the saprozone concentrates on the spatial spread of the pathogen and does not take account of the infection of the host by the pathogen, which may vary with host resistance.

Finally, we discuss how our findings obtained on a model pathosystem in controlled conditions could contribute to the design of new management strategies to control locally-spreading pathogens and reduce pesticide use on commercial crops.

## Materials and Methods

Three experiments were performed under the same controlled growth conditions. The two first assessed the effect of Monceren^®^ L on key epidemiological components (saprotrophic spread and infectivity) of the *R*. *solani/R*. *sativus* pathosystem. The third experiment tested the potential “shield effect” of Monceren^®^ L to contain pathogenic spread (i.e. from an infected host) in a site-specific application context, when its application is limited to a narrow strip of soil between the infected host and a recipient bait.

### The *Rhizoctonia solani*/*Raphanus sativus* pathosystem

*R*. *solani* is a soil-borne pathogenic basidiomycete with a wide host range [36]. It is known as a major soil-borne pathogen on several crops and causes various types of symptoms depending on host phenology at the time of infection, i.e. damping-off at early stages or necrosis and sclerotium formation later, particularly on tuberizing hosts [37]. Its spread in soil is sustained by organic matter (saprotrophic spread) or tissues of the infected host (pathogenic spread) through translocation processes [38,39]. The *R*. *solani* strain used (FM1, an anastomosis group 4 (AG4) strain isolated from lettuce in 2009 in southern France) is extremely sensitive to Monceren^®^ L (half maximal effective concentration (EC50), 45 μg/L), compared with other strains tested in [40]. The standard inoculum consisted of a 3 mm diameter mycelium disc produced on malt-agar medium following the methodology described in Gilligan and Bailey [41] and carefully placed at a depth of 5 mm in the soil. Bare soil pots consisted of pots with inoculum, but without hosts. Host inoculation pots were sowed with radish plants; in these pots, the mycelium disk was not placed on the host but rather in its immediate surroundings (5 mm away).

*R*. *sativus*, i.e. radish, is a fast-growing commercial host plant of *R*. *solani* and easy to experiment in climate chamber, and it has been used as a host model in several studies [32–34]. In this study we focused on only one cultivar which is known to be susceptible to *R*. *solani*. More precisely we used an early F1 hybrid widely used by French producers (Expo, Vilmorin S.A., France).

### Chemical control

For this work we used pencycuron, marketed in France as Monceren^®^ L (pencycuron 250 g/L), which is a protective phenylurea-based fungicide originally developed for selective control of *R*. *solani* on rice and potato [42,43]. It has since been tested on a variety of crops, alone or in combination with biological control [44–47]. Monceren^®^ L is a specific contact fungicide for *R*. *solani* and it is known to inhibit mitosis through its action on cellular microtubules [48]. After binding to the saturated fatty acids of the cell membrane, pencycuron appears to decrease the fluidity of the cell membrane and cause variation in the osmotic pressure of the cell [49,50]. These effects cause changes in cellular functions, such as the formation and dissociation of microtubules, which are involved in the migration of chromosomes during mitosis. In France, Monceren^®^ L is also registered for lettuce. It can be used in nursery on peat balled roots or in field conditions on vegetation. It can also be sprayed on soil after sowing, manner in which this chemical is usually employed by French vegetable producers [51].

### Microcosm, soil and host

Experiments were performed in a climate chamber (16 h:8 h photoperiod at 25°C, during the light period, lit by blue/red fluorescent lights and at 20°C during the dark period) with 50% humidity. Soil was a 50% v/v mix of 2.25 mm sieved sand (from the estuary of the Loire River, Montoir-de-Bretagne, France) and 2.25 mm sieved potting soil (NFU 44551, type 992016F1, Falienor S.A., Vivy, France). Soil moisture was maintained at 30% with daily tap water sub-irrigation. The soil was not sterilized, but the absence of pre-existing *R*. *solani* mycelium was checked during experiments using negative control pots. In treatments on hosts, one radish seed of cv. Expo F1 (Vilmorin S.A., France) was sown 25 mm deep at the centre of each pot. The first two experiments were conducted in polystyrene pots 7 x 7 x 6.2 cm filled with 160 cm^3^ of soil, and the third experiment was performed using pots consisting of PET cable trays (section 5.8 x 5 cm) with two layouts: one 14 cm long, filled with 320 cm^3^ of soil to assess pathogenic spread in soil at 25 mm and 50 mm, and the other 28 cm filled with 640 cm^3^ of soil to assess the probability of fungal spread at 75 mm. Pots were placed in trays and separated by a few centimetres to prevent the mycelium passing from one pot to another.

### Detection of *Rhizoctonia solani* in soil

To detect the presence or absence of *R*. *solani* in soil, a non-destructive capture technique was applied. Sterilized millet seeds used to bait *R*. *solani* were placed on the soil surface. Two days later, the bait was removed and placed in Petri dishes on a semi-selective medium: KHP medium without fenaminosulf and with nitrates instead of nitrites [52]. Presence or absence of *R*. *solani* was assessed after three days of incubation in a growth chamber (darkness; 20°C) using an optical microscope (50× magnification).

### Application of the fungicidal spray solution

Spray mix was prepared with commercial Monceren^®^ L and tap water, at the recommended dose for lettuce in nursery equivalent to 2 L/ha. To prevent experimenter bias, the application was performed with a spray gun (Spray Gun EARLEX HV2900V EU; spray mode equivalent to an even-flat fan nozzle) kept immobile at a fixed height from the ground, while the pots filed by on a small conveyor belt 38 cm below the spray gun. Spray distance and spray angles were chosen to ensure homogeneous application on pots, and checked using paper. All spray-gun settings were constant throughout experiments, and the spray mix was regularly replaced to prevent changes in flow rate due to low volume in the tank. Water control pots were sprayed using the same system with an equivalent volume of tap water. In Experiment 3 (see below), we used a cut-out to apply Monceren^®^ L on defined areas of the soil (strips of various widths).

### Placement experiments

#### Experiment 1: Impact of the fungicide on *R. solani* saprotrophic spread

The impact of Monceren^®^ L treatment on *R*. *solani* saprotrophic spread was quantified by inoculating bare soil and measuring the ability of the fungus to colonize millet seed baits located at three distances from the inoculum source (10, 12.5 or 25 mm). Each pot was inoculated, then sprayed with either Monceren^®^ L or tap water and then baits were placed on the soil surface. The baiting design consisted in placing four baits at a given distance (among the three distances: 10, 12.5 or 25 mm) from the inoculum source. There were 24 replicates per treatment and per distance. The spread of *R*. *solani* in soil was monitored for 2, 9 and 16 days after soil inoculation using the above-described baiting technique. For each treatment, we derived colonization profiles describing the probability of fungal colonization P(*x*,*t*) according to distance *x* and time *t* [50]. The experiment was repeated twice.

#### Experiment 2: Effect of the Monceren^®^ L treatment on pathogen infectivity

To assess the effect of Monceren^®^ L on the infectivity of the pathogen, radishes were placed with an *R*. *solani* inoculum in individual pots during the whole cropping period (30 days) with and without the fungicide. First, one radish seed was sown 25 mm deep at the centre of the pot; second, a mycelium disc was placed 5 mm deep in a 5 mm wide area above the sowing point; and third, the pot was sprayed with either Monceren^®^ L or tap water. There were 20 replicates per treatment. Pots without inoculum were used to check the absence of *R*. *solani* in soil and on seeds. Thirty days after sowing, symptoms were assessed either as “damping-off” (necrosis on the radish collar, cotyledons and leaves, after which the seedling falls over onto the soil surface) or “necrosis” (a dark spot on the tuber only). Damping-off incidence (DO) and disease incidence (DI) were calculated as:

DO = (number of hosts affected by damping−off/total number of emerged hosts) × 100

DI = (number of infected hosts/total number of emerged hosts) × 100

This experiment was repeated twice.

#### Experiment 3: Efficiency of site-specific applications for controlling pathogenic spread

The site-specific application investigated here consisted in a strip placed between the infected host-donor and a recipient bait. Monceren^®^ L was applied, taking care to leave the sowing row untreated within 12.5 mm on either side (total untreated width, 25 mm). We measured the effectiveness of this spraying design by quantifying the proportion of colonized recipient baits located at three distances (25, 50 and 75 mm) from the sowing row, corresponding to the three treated strip widths (12.5, 37.5 and 62.6 mm, respectively). The spread of *R*. *solani* in soil was assessed for 2, 9 and 16 days after host inoculation. At the end of the experiment, radishes were checked for the presence or absence of symptoms (because some symptoms are underground). Only data from the pots with infected hosts were kept for the analysis of pathogenic spread. There were 14 replicates per treatment and per distance and the experiment was repeated twice.

### Statistical analysis

In Experiment 1, the impact of chemical treatment on saprotrophic spread was assessed using generalized linear mixed models (GLMMs) [53] (“lme4” package) for binary data (distribution: binomial, link: logit). A separate model was built for each distance (10, 12.5 and 25 mm). In all of these models, treatment (tap water or Monceren^®^ L at 2 L/ha) was included as a fixed factor, time as a covariable and their interaction as a fixed factor. Repetitions and pots nested in repetition were included as random factors. The effects of treatment, time and their interaction were tested using a Wald test.

In Experiment 2, the effect of pencycuron treatment on the proportion of infected plants, the proportion of plants with damping-off and plants with necrosis was assessed using GLMMs for binary data (distribution: binomial, link: logit), separately for disease incidence (DI), damping-off incidence (DO) and necrosis. In each model, treatment (tap water or pencycuron) was included as a fixed factor. Repetition was included as a random factor. The effects of treatment were tested using a Wald test.

In Experiment 3, the potential localized shield effect of Monceren^®^ L against pathogenic spread was quantified through the ability of the fungus to pass through treated strips of soil and colonize baits located at three distances (25, 50 and 75 mm). For each distance, a GLMM model was built with treatment (tap water or pencycuron 2 L/ha) as a fixed factor, time as a covariable and their interaction as a fixed factor. Repetitions and pots nested in repetition were included as random factors. The effects of treatment, time and their interaction were tested using a Wald test.

All statistical analyses were carried out using R version 3.1.0 (R Development Core Team, 2014).

### Saprozone Modelling

According to the available knowledge on *R*. *solani*, we considered that saprozone profiles, i.e. surfaces describing the probability of fungal colonization P(x,t) according to distance *x* and time *t*, can vary with the inoculum source and the level of nutrients it contains. We used two previously developed, quasi-mechanistic pathozone models for describing saprozone profiles in two contrasting situations: (i) when the inoculum source contains a limited level of nutrients for the fungus and (ii) when the inoculum source provides unlimited nutrients during the considered period (typically large infected hosts). These models describe respectively the rate of saprotrophic spread (A1) and the rate of pathogenic spread (A2) by combining the diminishing effects of several biological processes on a basic (maximum) rate of colonization:

$${Α}_{1}=\left(\begin{array}{c} Maximum\\ rate\\ of\\ colonization\\ (\alpha_{1)} \end{array}\right)\left(\begin{array}{c} Delay\\ in\\ onset\;of\\ colonization\\ \left(\vartheta_{1}\right) \end{array}\right)\left(\begin{array}{c} Spatial\;decline\\ due\;to\\ distance\;from\\ inoculum\\ \left(\phi_{1}\right) \end{array}\right)\left(\begin{array}{c} Time\;deccline\\ due\;to\\ nutrients\\ depletion\\ \left(\psi_{1}\right) \end{array}\right)$$

$${Α}_{2}=\left(\begin{array}{c} Maximum\\ rate\\ of\\ colonization\\ (\alpha_{2)} \end{array}\right)\left(\begin{array}{c} Delay\\ in\\ onset\;of\\ colonization\\ \left(\vartheta_{2}\right) \end{array}\right)\left(\begin{array}{c} Spatial\;decline\\ due\;to\\ distance\;from\\ inoculum\\ \left(\phi_{2}\right) \end{array}\right)$$

After some derivation, (see details in [54]), we obtained explicit models for the dynamics of the saprozone:

$$P_{1}\left(x,t\right)=1-exp\left[-\alpha_{1}e^{-\sigma_{1}x^{2}}\frac{e^{-d_{1}\delta_{1}x}-e^{-d_{1}\left(t-\tau_{1}\right)}}{d_{1}}\theta \left(t-\tau_{1}-\delta_{1}x\right)\right]$$ (1)

$$P_{2}\left(x,t\right)=1-exp\left[-\alpha_{2}e^{-\sigma_{2}x^{2}}\left(t-\tau_{2}-\delta_{2}x\right)\theta \left(t-\tau_{2}-\delta_{2}x\right)\right]$$ (2)

where *t* is the time since soil or host inoculation, and *x* is the distance from inoculum. We assessed and parameterized the models for the rate of saprotrophic and pathogenic spread Eqs (1) and (2) by fitting the experimental data with the following random process, which describes the number of baits that were colonized:

$$n_{col}\left(x,t\right)\sim Binomial\left(n_{tot},P\left(x,t\right)\right)$$ (3)

where *P = P*_1_ or *P*_2_ is the probability of colonization given by Eqs (1) or (2). Specifically, we fitted *n*_*col*_ to the number of colonized baits among *n*_*tot*_ replicates, for distance *x* and time after inoculation *t*. We implemented this estimation of the parameters in Eqs (1) and (2) via Bayesian Markov chain-Monte Carlo sampling with a likelihood function based on Eq (3) and non-informative prior distributions, run in OpenBugs [55] and with outputs analysed in R [56]. We verified the consistency of the models with the observed data by checking that the observations of the probability of colonization P(x,t) were contained within the posterior predictive distribution of the fitted models (1) and (2) (see S1 File).

These two models were used for analysing data obtained in Experiments 1 and 3 and assessed the impacts of the fungicide on saprozone behaviour by comparing treatments (fungicide vs tap water).

## Results

### Experiment 1: Impact of the fungicide on *R*. *solani* saprotrophic spread

#### Exploratory and statistical analysis

**Fig 1:**
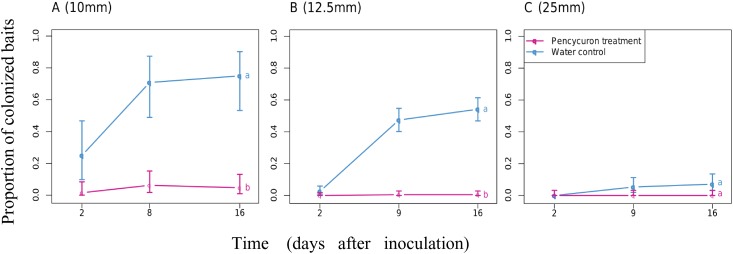
Proportion of recipient baits colonized by *Rhizoctonia solani* upon saprotrophic spread at (A) 10 mm, (B) 12.5 mm and (C) 25 mm after treatment with Monceren^®^ L (pink lines) or tap water (blue lines). Different letters indicate significant differences in the proportion of colonized baits (P-value < 0.05, Wald test).

In the absence of fungicide, the fungus was able to quickly colonize the surrounding soil surface. As shown in Fig 1A, 25% of recipient baits placed 10 mm away from the mycelium disc were colonized within 2 days after inoculation and a large proportion (75%) of recipient baits were colonized after 16 days. However, without a large source of nutrients (e.g. host), saprotrophic spread was spatially limited and declined abruptly with distance. At the end of the experiment, 54% of baits were colonized at 12.5 mm (Fig 1B) and only 7% at a distance of 25 mm (Fig 1C). The Monceren^®^ L treatment radically altered colonization profiles (Fig 1). Fungicide treatment restricted saprotrophic mycelial growth to a smaller perimeter and colonization did not exceed 12.5 mm. Furthermore, the probability of saprotrophic spread was lower than that observed with the tap water control. There were significant differences between colonization profiles with or without fungicide only at short distances (10 mm, p<0.001, and 12.5 mm, p<0.001).

#### Saprozone model

**Fig 2:**
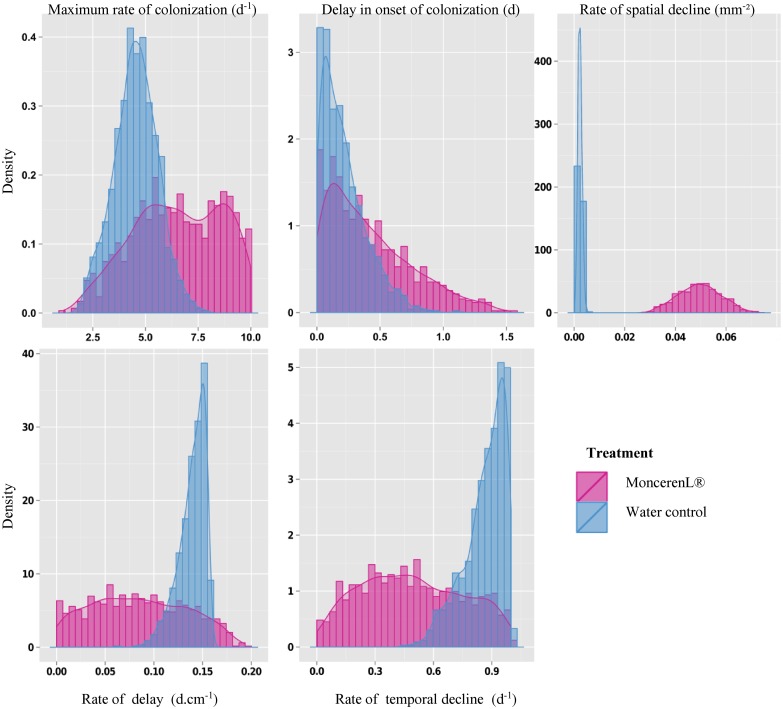
Histogram describing the posterior distributions of the parameters of the saprotrophic spread model.

**Fig 3:**
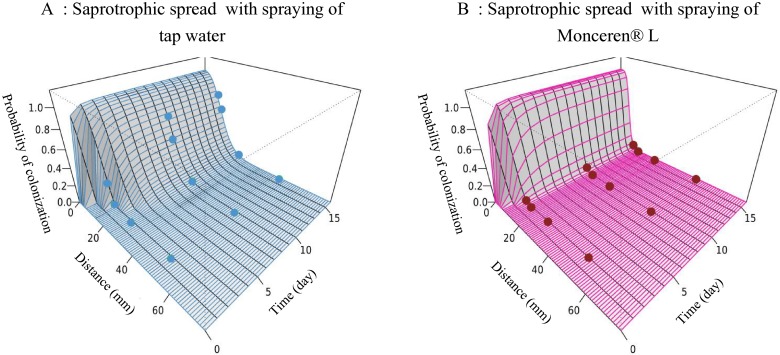
Saprozone dynamics for saprotrophic spread of *Rhizoctonia solani*, describing the change in the probability colonization for a bait placed at a given distance from a mycelium disc and after a given time of exposure. Circles indicate data observed in the placement experiments.

Although some parameters were difficult to estimate (Table 1 and Fig 2), the saprozone model (A1) captured the general overall pattern of the data (Fig 3 and S1 File), allowing us to analyse the effects of fungicide application—the main aim of this experiment. While the statistical inference provided relatively satisfactory estimations with narrow posterior distributions for the water treatment, the fit of the model to the fungicide treatment data showed higher uncertainty in the Bayesian estimation of parameters (wider posterior distributions). The comparison between treatments corroborates our empirical analysis and provided a better visualization and mechanistic understanding of the effect of the fungicide on mycelial spread through soil. Our results suggest that the fungicide most affected the rate of spatial decline (σ1) and thus, the ability of the fungus to develop its mycelial network in space from a primary propagule (i.e. from the mycelium disc). The estimated median of the spatial decline parameter (σ1) in the water treatment (control) was 25 times higher in the Monceren^®^ L treatment (0.002 and 0.049 mm², respectively; Table 1). The observed local discrepancies of model fitting and the uncertainty in parameter estimation for the fungicide treatment may be due to the lack of information at critical points of the saprozone profiles, and/or to the need to relax and modify some assumptions of the model.

**Table 1:** Values of the parameters of the model for saprotrophic spread and estimated distributions.

Parameter	Symbol	Unit	Treatment	Mean	SD	q-2.5%	Median	q-97.5%
**Maximum rate of colonization**	**α1**	d^-1^	Water	4.498	1.013	2.631	4.501	6.595
			MoncerenL^®^	6.542	2.030	2.855	6.577	9.831
**Delay in onset of colonization**	**τ1**	d	Water	0.208	0.172	0.007	0.163	0.649
			MoncerenL^®^	0.412	0.325	0.011	0.332	1.160
**Rate of spatial decline**	**σ1**	mm^-2^	Water	0.002	0.0008	0.0008	0.002	0.004
			MoncerenL^®^	0.049	0.0078	0.0338	0.049	0.064
**Rate of delay**	**δ1**	d cm^-1^	Water	0.139	0.0137	0.103	0.143	0.156
			MoncerenL^®^	0.085	0.0480	0.0065	0.083	0.173
**Rate of temporal decline**	**d**_**1**_	d^-1^	Water	0.867	1.01	0.618	0.888	0.997
			MoncerenL^®^	0.504	0.260	0.061	0.501	0.958

### Experiment 2: Effect of the Monceren^®^ L treatment on pathogen infectivity

**Fig 4:**
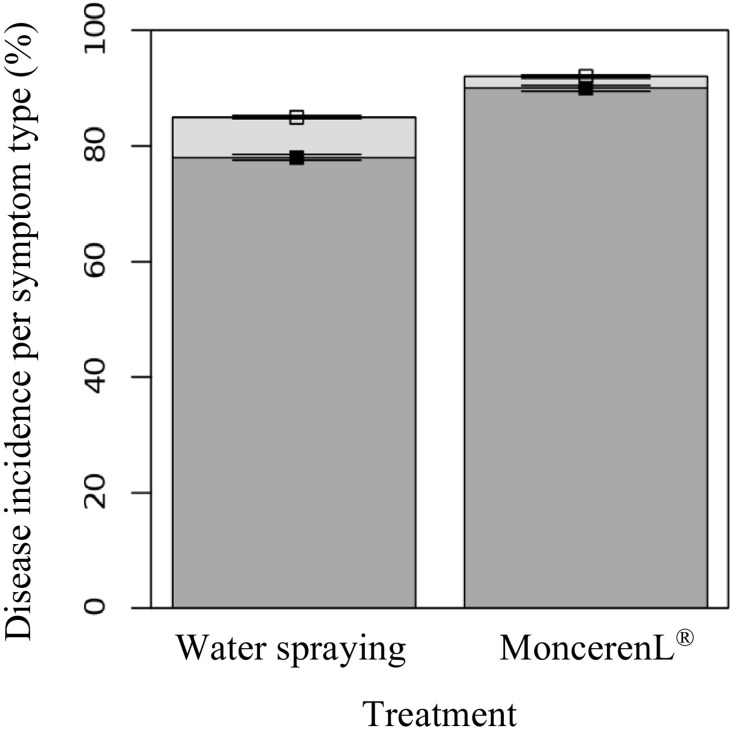
Effect of fungicide treatment on pathogen infectivity. Bars show the mean cumulated incidence at harvest (i.e. 30 days after sowing); dark and light grey refer to the type of symptom: damping-off and tuber necrosis, respectively. The analysis of deviance using a Wald chi-square test shows no significant differences between treatments.

The Monceren^®^ L treatment had no significant effect on pathogen infectivity (Fig 4). Disease incidence (DI) was not significantly affected by the presence of Monceren^®^ L (p = 0.271). Given that inoculations were performed during the seedling stage, they led to a very high proportion of damping-off disease with (90 ± 5%) or without (78 ± 5%) the fungicide. The statistical analyses we performed showed no significant differences between treatments for the proportion of damping-off (p = 0.116). Only a few necroses were observed with the water treatment (7 ± 3%) and only one plant showing necrosis symptoms (2 ± 3%) was observed in the fungicide treatment. The analysis of deviance using Wald chi-square test showed no significant differences between treatments for the proportion of necroses (p = 0.317). In short, our findings show the ineffectiveness of Monceren^®^ L treatment against the pathogen infectivity once the fungus had reached its host.

### Experiment 3: Efficiency of site-specific application for controlling pathogenic spread

#### Exploratory and statistical analysis

**Fig 5:**
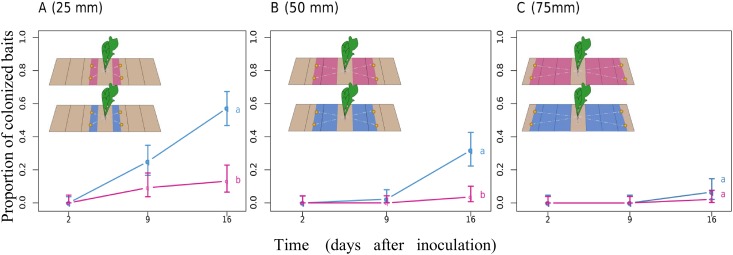
Proportion of recipient baits colonized by *Rhizoctonia solani* upon pathogenic spread at (A) 25 mm, (B) 50 mm and (C) 75 mm after treatment with Monceren^®^ L (pink lines) or tap water (blue lines). Different letters indicate significant differences in the proportion of colonized baits (P-value < 0.05, Wald test).

Previous studies [33,37] have already reported that pathogenic spread is more extensive than saprotrophic spread. The exploitation of the resources of the infected host allows the fungus to sustain its spread through translocation processes. Fig 5 gives the proportion of recipient baits colonized by *R*. *solani* after infection and exploitation of a host according to treatment. Compared with saprotrophic spread in Experiment 1 (Fig 1), there were more colonization profiles due to pathogenic spread after treatment with tap water at 25 mm distance (Fig 5A), with 25% of baits 9 days after inoculation and 57% of baits being colonized after 16 days. Furthermore, the utilization of the infected host augmented the mycelial development of *R*. *solani* which was observed at longer distances: 16 days after inoculation 32% of baits placed 50 mm away from the infected host were colonized and 7% of baits at 75 mm.

In contrast, the spatially localized spraying of Monceren^®^ L greatly decreased the pathogenic spread of *R*. *solani* in soil (Fig 5). A 12.5 mm wide treated soil strip was enough to contain fungal spread and lower the proportion of colonized baits located at 25 mm from 57% to 13% 16 days after inoculation (Fig 5A). At greater widths of Monceren^®^ L-treated soil strips, the proportion of colonized baits decreased from 32% to 4% (Fig 5B) at 50 mm and to 2% at 75 mm (Fig 5C). The statistical analysis showed significant differences between treatments for the proportion of colonized baits located at 25 mm (p<0.001) and at 50 mm (p<0.001).

#### Saprozone model

**Fig 6:**
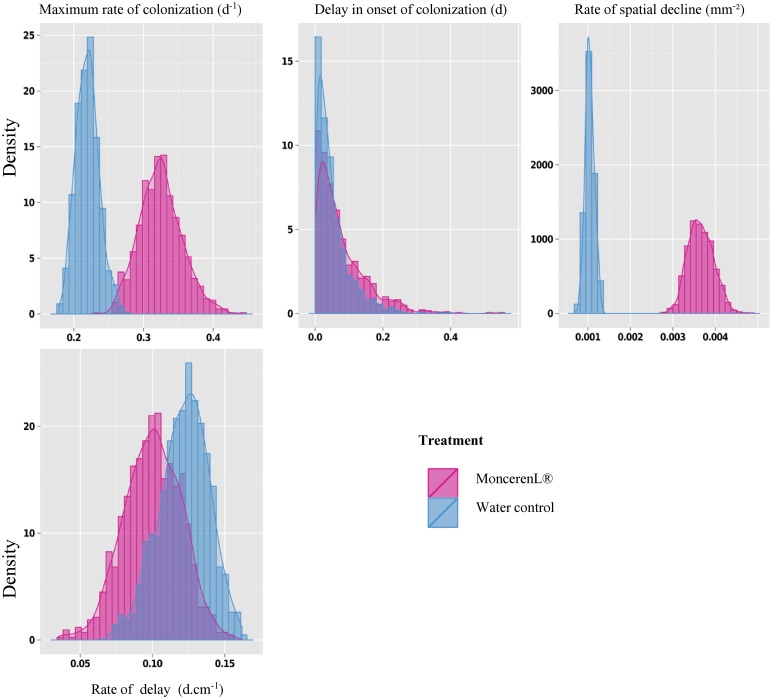
Histogram describing the estimated distribution of the parameters of the pathogenic spread model.

**Fig 7:**
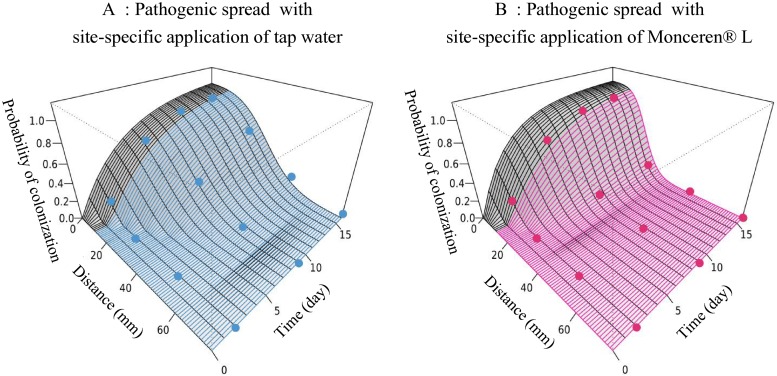
Saprozone dynamics for pathogenic spread of *Rhizoctonia solani*, describing the change in the probability of colonization for bait placed at a given distance from a mycelium disc and after a given time of exposure. Circles indicate data observed in the placement experiments. Data were fitted to the model.

Disregarding the discrepancies at the 25 mm distance (Fig 6 and S1 File), the pathogenic spread model integrating four parameters captured the general pattern of the data and fit the data quite well, relating the proportion of recipient baits colonized by mycelial pathogenic spread. This good fit allowed us to (i) better interpret our statistical analyses and (ii) analyse the effects of fungicide application on the parameters affecting saprozone behaviour (Table 2 and Fig 7).

**Table 2:** Values of the parameters of the model for pathogenic spread and estimated distributions.

Parameter	Symbol	Unit	Treatment	Mean	SD	q-2.5%	Median	q-97.5%
**Maximum rate of colonization**	**α2**	d^-1^	Water	0.220	0.016	0.191	0.220	0.257
			MoncerenL^®^	0.324	0.032	0.266	0.324	0.395
**Delay in onset of colonization**	**τ2**	d	Water	0.048	0.048	0.0008	0.032	0.173
			MoncerenL^®^	0.072	0.071	0.0024	0.049	0.265
**Rate of spatial decline**	**σ2**	mm^-2^	Water	0.0010	0.0001	0.0008	0.001	0.0012
			MoncerenL^®^	0.0036	0.0002	0.003	0.0036	0.0042
**Rate of delay**	**δ2**	d cm^-1^	Water	0.121	0.017	0.086	0.121	0.152
			MoncerenL^®^	0.100	0.020	0.061	0.100	0.138

Similar to model (1), the fungicide treatment mostly affected the spatial rate of decline (σ2). Although the estimated median was 0.001 with water, it was 0.003mm^-^² with the fungicide. Because only a slight change in the rate of delay (δ2) was detected, the effect of the fungicide may not be significant on this parameter. In conclusion, as illustrated in Fig 7 and Table 2, the maximum rate of colonization (α2) appeared to be greater in the presence of the fungicide. This result cannot be interpreted with respect to the biology and the epidemiology of the pathosystem, and may indicate a lack of information at critical points of the pathozone profiles (i.e. measurements between 0 and 10 mm).

## Discussion

In line with previous work that investigated the effects of biological or chemical treatments on epidemiological processes and the life-history traits of plant pathogens, we have confirmed that using epidemiological concepts and non-linear models may help understand how treatments impact the processes involved in the spatial spread of diseases. Such approaches are central to design innovative pest management strategies that would reduce the impact on the environment (e.g. based on a low pesticide use) while insuring the income of growers. By considering the model pathosystem *R*. *solani/R*. *sativus* and its main chemical control product Monceren^®^ L, we investigated the effects of chemical treatment on both saprotrophic spread (i.e. local dispersal from a mycelium disc) and pathogen infectivity. We then tested the potential localized “shield effect” of Monceren^®^ L on the pathogenic spread (i.e. local dispersal from an infected host) in a site-specific application context, with localized application confined to a strip of soil between the infected host and a recipient bait. Following previous studies we fitted simple mechanistic mathematical models to our experimental data to assess the effects of treatments on epidemiological parameters and support our statistical analyses. We used models initially developed to analyze the pathozone behavior and which performed well enough to assess the effects of the fungicide on the spread of the pathogen through the soil (saprozone).

Although the analyses of our experimental data through empirical statistical analysis and quasi-mechanistic modelling indicated that the fungicide appears to limit the spatial expansion of mycelia (Fig 1), which corroborates the observations made by Kleczkowski et al [33] that primary infections in this pathosystem do not occur beyond 20 mm, we found no significant effect of the chemical treatment on infections of host plants when they are in very close proximity or in direct contact with the inoculum (Fig 4). This unexpected pattern suggests that spraying the chemical product on the soil surface would be ineffective in preventing infections of crops by infectious propagules that lie a few millimetres below the surface and in contact with host root tissues. This lack of effect might be explained by a slow diffusion [57] or weak effect of the toxic molecules on the pathogen when it lies in the rhizosphere of its host [58,59] where the root exudates highly stimulate its development. However, further studies on different soil conditions, strains and cultivars would be needed to confirm (or infirm) these findings. The observed effect of the chemical treatment on mycelial growth showed that even localized fungicide applications can limit and quench the transmission of *R*. *solani* between neighboring plants. Regarding previous studies that have tested the percolation theory on the spread of *R*. *solani*, the shield effect between close hosts suggests that the local control of a certain (yet unknown) proportion of individuals with the fungicide has the potential to slow down epidemics and turn an initially invasive system into a non-invasive state (phase transition). Thus, it would be interesting to use recent technical advances in precision agriculture for testing the efficacy of management strategies based on local applications of chemical products in field conditions and for various crops susceptible to *R*. *solani*. The most simple preventive strategy would involve treating thin strips of soil between plants or seeds (e.g. between rows) to restrict the spread of infection. This strategy can be expanded to optimize efficient management strategies based on localized treatment to minimize the risk of disease within uncertain environmental-socio-economic contexts [60]. A curative disease management strategy based on localized chemical treatments during the growth period may be a less costly strategy, but relies heavily on detailed knowledge on the cryptic development of epidemics and on the ability to detect early infections or symptomatic individuals.

Once again, our study highlights the importance of the translocation process for the mycelial spread of *R*. *solani* when the pathogen has access to a large source of nutrients [38,39]. In fact, our results suggest that pathogenic fungal spread can extend further than saprotrophic spread. However, regardless of the source of the inoculum, chemical treatment strongly affected the saprozone, in particular by inducing an increase in the spatial rate of decline (Table 1). Therefore, Monceren^®^ L seems to affect the pathogen even when it has access to nutrients for supplying its growth.

In conclusion, our results give valuable insight into how the particular *R*. *solani*/radish pathosystem may be efficiently controlled with local applications of the commercial fungicide Monceren^®^ L, but further investigation is still needed to determine to what extent these results can be expanded to other pathosystems, cultivars and fungicide products. In fact, some studies have shown that the spatial spread and the infectivity of soil-borne pathogens as well as the fungicide effect can vary among pathogen strains, cultivars, the toxic molecules considered and the dose applied on the crop [57,61,62]. Thus, it would be relevant to run similar studies on main commercial crops for which soil-borne pathogens, which are still difficult to manage [63], cause substantial losses in order to assess the genericity or specificity of our findings and suggestions. However, as pathosystems involving *R*. *solani* are likely to behave in a similar way in field conditions than in microcosms [54], we expect that our results might be qualitatively expanded to such pathosystems when treated with pencycuron.

## Supporting Information

S1 File Saprozone uncertainty and observations. (DOCX)

S2 File Dataset related to experiment 1. This pdf file contains raw data used in our analysis to assess the effect of Monceren^®^ L on saprotrophic spread.
(PDF)

S3 File Dataset related to experiment 2. This pdf file contains raw data used in our analysis to assess the effect of Monceren^®^ L on pathogen infectivity.
(PDF)

S4 File Dataset related to experiment 3. This pdf file contains raw data used in our analysis to assess the effect of Monceren^®^ L on pathogenic spread.
(PDF)
