# Supplementary material for: Reducing the Use of Pesticides with Site-Specific Application: The Chemical Control of Rhizoctonia solani as a Case of Study for the Management of Soil-Borne Diseases
Source: PLoS One. 2016 Sep 26;11(9):e0163221. doi: 10.1371/journal.pone.0163221 (PMC5036793; doi:10.1371/journal.pone.0163221)
Supplement: S1 File — (DOCX) [file pone.0163221.s001.docx]

**Supporting information S1**

Supporting information S1. Saprozone uncertainty and observations

**Methodology**

The consistency of mechanistic models describing the spatio-temporal process of the rate of saprotrophic spread (1) and the rate of pathogenic spread (2) were assessed by checking that the experimental observations were contained within the posterior predictive distributions of the fitted pathozone models.

$P_{1}\left( \mathcal{x},\mathcal{t} \right)=1-exp\left[ -\alpha_{1}\mathcal{e}^{-\sigma_{1}\mathcal{x}^{2}}\frac{\mathcal{e}^{-d_{1}\delta_{1}x}-e^{-d_{1}\left( t-\tau_{1} \right)}}{d_{1}}\theta(t-\tau_{1}-\delta_{1}x) \right]$ (1)

$P_{2}\left( \mathcal{x},\mathcal{t} \right)=1-exp\left[ -\alpha_{2}\mathcal{e}^{-\sigma_{2}\mathcal{x}^{2}}\left( t-\tau_{2}-\delta_{2}x \right)\theta(t-\tau_{2}-\delta_{2}x) \right]$ (2)

*A posteriori* distributions of the probability of infection were obtained by simulating the model with parameters sampled from the posterior distributions of the model parameters. These parameter posterior distributions were previously obtained using Bayesian Markov chain-Monte Carlo sampling (Gibbs sampling) using a likelihood function based on (3) and the experimental data, and non-informative prior distributions of the model parameters.

Posterior distributions of the probability of infection were represented with box-and-whisker plots (boxplots) which gave a good overview of the simulated data distributions and provided a visual check of the experimental data and its potential outliers.

$\mathcal{n}_{col}\left( \mathcal{x},\mathcal{t} \right)\sim Binomial(n_{tot}, P\left( \mathcal{x},\mathcal{t} \right))$ (3)

**Results**

***Saprotrophic spread***


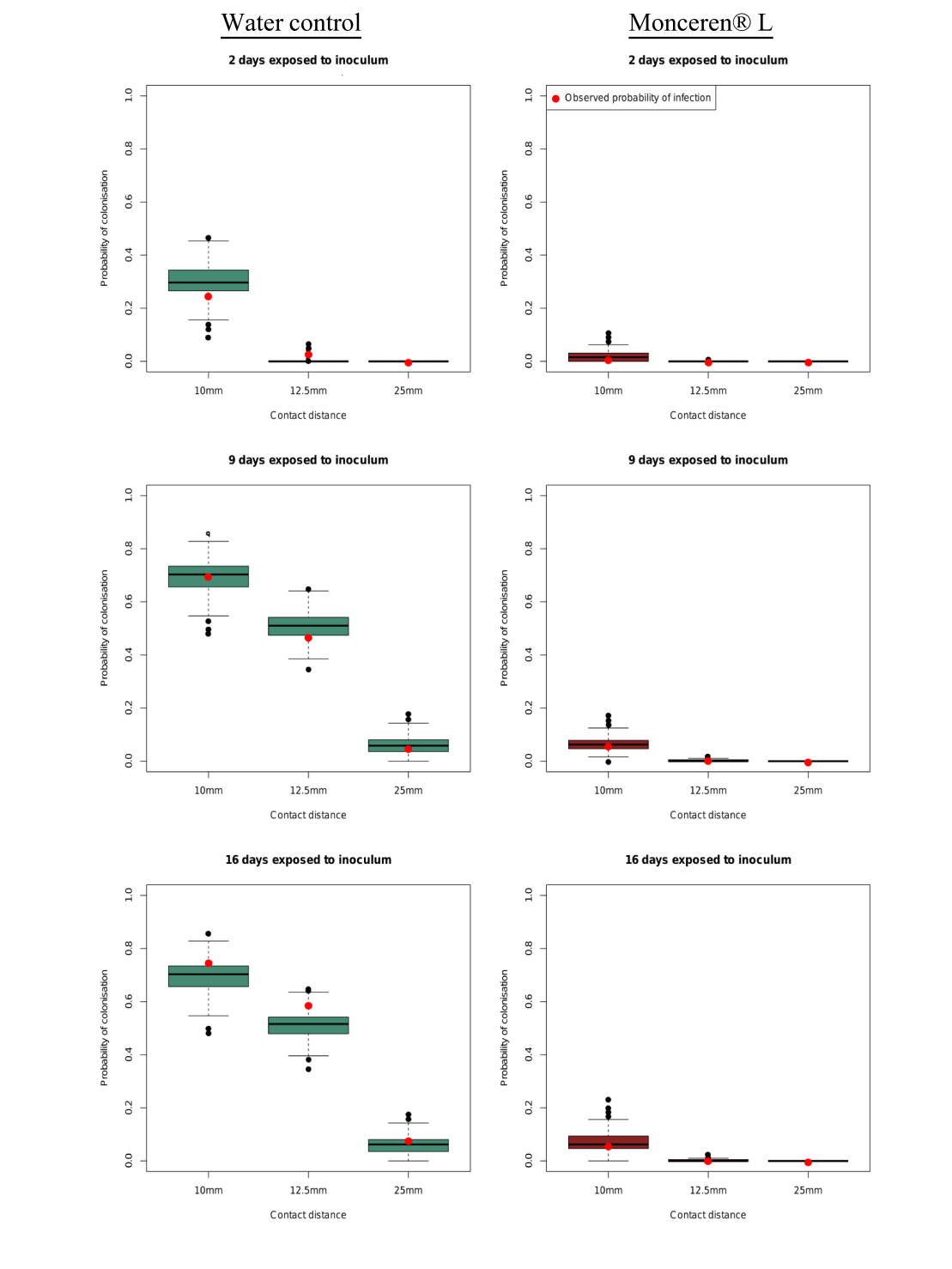


Figure S1.1 Posterior profile for saprotrophic spread. *A posteriori* distributions of probabilities of colonization P_1_(x,t) for a bait placed at a given contact distance (x=10, 12.5 and 25 mm) from an inoculum (i.e. mycelium disc) after a given time of exposure (t = 2, 9 and 16 days) after treatment with water (green) or Monceren® L (brown). Observed probabilities of colonization are represented by filled red circles.

***Pathogenic spread***


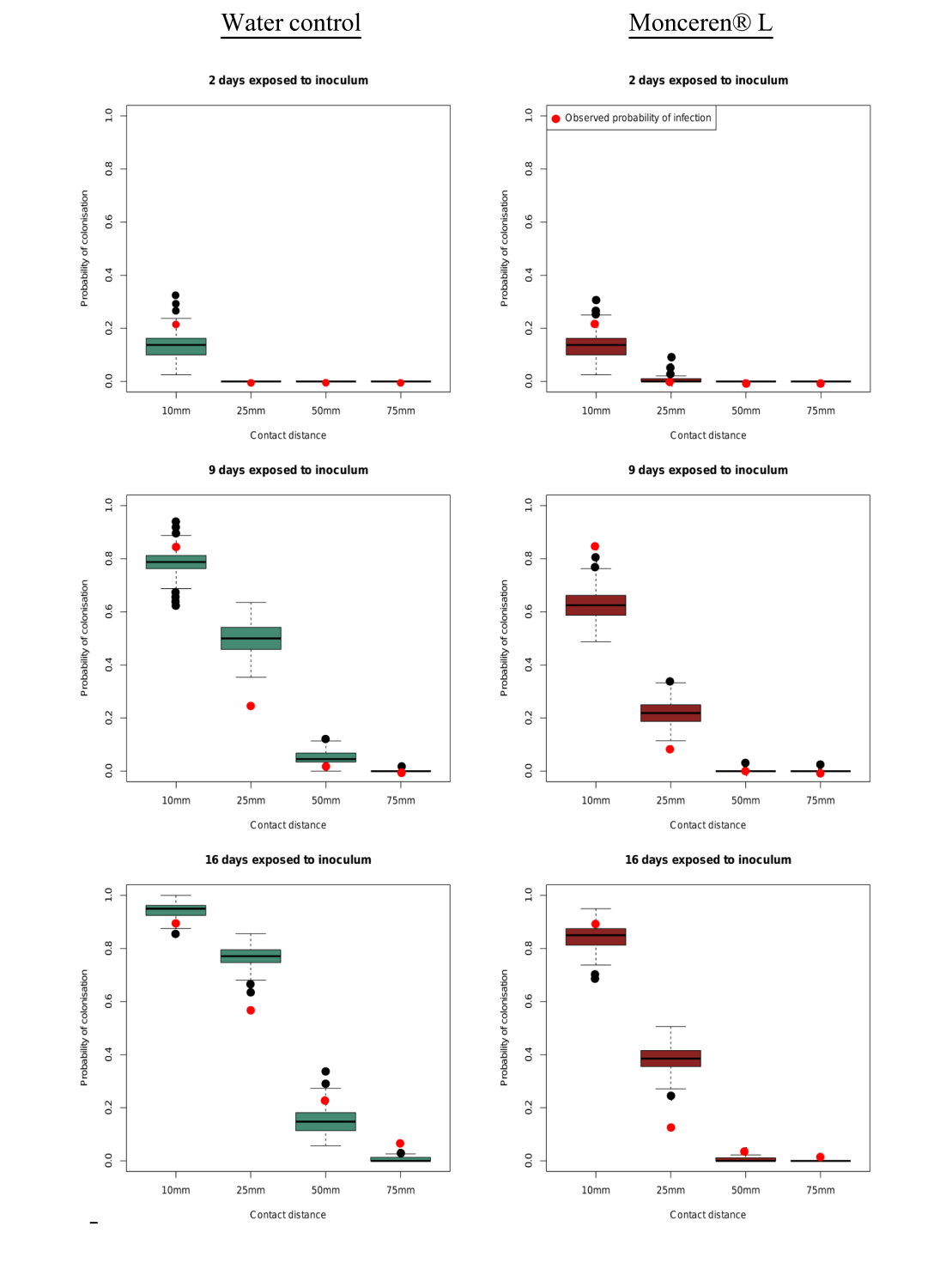


Figure S1.2 Posterior profile for pathogenic spread. *A posteriori* distributions of probabilities of colonization P_2_(x,t) for a bait placed at a given contact distance (x=10, 25, 50 and 75 mm) from an inoculum (i.e. mycelium disc) after a given time of exposure (t = 2, 9 and 16 days) after treatment with water (green) or Monceren® L (brown). Observed probabilities of colonization are represented by filled red circles.
