# Supplementary material for: Reducing the Use of Pesticides with Site-Specific Application: The Chemical Control of Rhizoctonia solani as a Case of Study for the Management of Soil-Borne Diseases
Source: PLoS One. 2016 Sep 26;11(9):e0163221. doi: 10.1371/journal.pone.0163221 (PMC5036793; doi:10.1371/journal.pone.0163221)
Supplement: S2 File — This pdf file contains raw data used in our analysis to assess the effect of Monceren® L on saprotrophic spread. (PDF) [file pone.0163221.s002.pdf]

<Metadata>

Manip = repetition

Mod = ML+ for Moncern®L spraying, ML- for water spraying

Dis = Distance

Pot = Pot

Bait = Bait

Time = number of days after inoculation when the baits are placed in the semi-selective medium

Col = 1 if bait is colonized, otherwise = 0

</Metadata>

<Data>

| Manip | Modalite | Dis | Pot | Bait | Time | Col |
|---|---|---|---|---|---|---|
| M1 | ML+ | D10 | P1 | 1 | 2 | 0 |
| M1 | ML+ | D10 | P1 | 2 | 2 | 0 |
| M1 | ML+ | D10 | P1 | 3 | 2 | 0 |
| M1 | ML+ | D10 | P1 | 4 | 2 | 0 |
| M1 | ML+ | D10 | P2 | 1 | 2 | 0 |
| M1 | ML+ | D10 | P2 | 2 | 2 | 0 |
| M1 | ML+ | D10 | P2 | 3 | 2 | 0 |
| M1 | ML+ | D10 | P2 | 4 | 2 | 0 |
| M1 | ML+ | D10 | P3 | 1 | 2 | 0 |
| M1 | ML+ | D10 | P3 | 2 | 2 | 0 |
| M1 | ML+ | D10 | P3 | 3 | 2 | 0 |
| M1 | ML+ | D10 | P3 | 4 | 2 | 0 |
| M1 | ML+ | D10 | P4 | 1 | 2 | 0 |
| M1 | ML+ | D10 | P4 | 2 | 2 | 0 |
| M1 | ML+ | D10 | P4 | 3 | 2 | 0 |
| M1 | ML+ | D10 | P4 | 4 | 2 | 0 |
| M1 | ML+ | D10 | P5 | 1 | 2 | 0 |
| M1 | ML+ | D10 | P5 | 2 | 2 | 0 |
| M1 | ML+ | D10 | P5 | 3 | 2 | 0 |
| M1 | ML+ | D10 | P5 | 4 | 2 | 0 |
| M1 | ML+ | D10 | P6 | 1 | 2 | 0 |
| M1 | ML+ | D10 | P6 | 2 | 2 | 0 |
| M1 | ML+ | D10 | P6 | 3 | 2 | 0 |
| M1 | ML+ | D10 | P6 | 4 | 2 | 0 |
| M1 | ML+ | D10 | P7 | 1 | 2 | 0 |
| M1 | ML+ | D10 | P7 | 2 | 2 | 0 |
| M1 | ML+ | D10 | P7 | 3 | 2 | 0 |
| M1 | ML+ | D10 | P7 | 4 | 2 | 0 |
| M1 | ML+ | D10 | P8 | 1 | 2 | 0 |
| M1 | ML+ | D10 | P8 | 2 | 2 | 0 |
| M1 | ML+ | D10 | P8 | 3 | 2 | 0 |
| M1 | ML+ | D10 | P8 | 4 | 2 | 0 |
| M1 | ML+ | D10 | P9 | 1 | 2 | 0 |
| M1 | ML+ | D10 | P9 | 2 | 2 | 0 |
| M1 | ML+ | D10 | P9 | 3 | 2 | 0 |
| M1 | ML+ | D10 | P9 | 4 | 2 | 0 |
| M1 | ML+ | D10 | P10 | 1 | 2 | 0 |
| M1 | ML+ | D10 | P10 | 2 | 2 | 0 |

| | | | | | | |
|---|---|---|---|---|---|---|
| M1 | ML+ | D10 | P10 | 3 | 2 | 0 |
| M1 | ML+ | D10 | P10 | 4 | 2 | 0 |
| M1 | ML+ | D10 | P11 | 1 | 2 | 0 |
| M1 | ML+ | D10 | P11 | 2 | 2 | 0 |
| M1 | ML+ | D10 | P11 | 3 | 2 | 0 |
| M1 | ML+ | D10 | P11 | 4 | 2 | 0 |
| M1 | ML+ | D10 | P12 | 1 | 2 | 0 |
| M1 | ML+ | D10 | P12 | 2 | 2 | 0 |
| M1 | ML+ | D10 | P12 | 3 | 2 | 0 |
| M1 | ML+ | D10 | P12 | 4 | 2 | 0 |
| M1 | ML+ | D10 | P13 | 1 | 2 | 0 |
| M1 | ML+ | D10 | P13 | 2 | 2 | 0 |
| M1 | ML+ | D10 | P13 | 3 | 2 | 0 |
| M1 | ML+ | D10 | P13 | 4 | 2 | 0 |
| M1 | ML+ | D10 | P14 | 1 | 2 | 0 |
| M1 | ML+ | D10 | P14 | 2 | 2 | 0 |
| M1 | ML+ | D10 | P14 | 3 | 2 | 0 |
| M1 | ML+ | D10 | P14 | 4 | 2 | 0 |
| M1 | ML+ | D10 | P15 | 1 | 2 | 0 |
| M1 | ML+ | D10 | P15 | 2 | 2 | 0 |
| M1 | ML+ | D10 | P15 | 3 | 2 | 0 |
| M1 | ML+ | D10 | P15 | 4 | 2 | 1 |
| M1 | ML+ | D10 | P16 | 1 | 2 | 0 |
| M1 | ML+ | D10 | P16 | 2 | 2 | 0 |
| M1 | ML+ | D10 | P16 | 3 | 2 | 0 |
| M1 | ML+ | D10 | P16 | 4 | 2 | 0 |
| M1 | ML- | D10 | P1 | 1 | 2 | 0 |
| M1 | ML- | D10 | P1 | 2 | 2 | 1 |
| M1 | ML- | D10 | P1 | 3 | 2 | 0 |
| M1 | ML- | D10 | P1 | 4 | 2 | 0 |
| M1 | ML- | D10 | P2 | 1 | 2 | 0 |
| M1 | ML- | D10 | P2 | 2 | 2 | 0 |
| M1 | ML- | D10 | P2 | 3 | 2 | 0 |
| M1 | ML- | D10 | P2 | 4 | 2 | 0 |
| M1 | ML- | D10 | P3 | 1 | 2 | 0 |
| M1 | ML- | D10 | P3 | 2 | 2 | 1 |
| M1 | ML- | D10 | P3 | 3 | 2 | 0 |
| M1 | ML- | D10 | P3 | 4 | 2 | 0 |
| M1 | ML- | D10 | P4 | 1 | 2 | 0 |
| M1 | ML- | D10 | P4 | 2 | 2 | 0 |
| M1 | ML- | D10 | P4 | 3 | 2 | 1 |
| M1 | ML- | D10 | P4 | 4 | 2 | 1 |
| M1 | ML- | D10 | P5 | 1 | 2 | 0 |
| M1 | ML- | D10 | P5 | 2 | 2 | 0 |
| M1 | ML- | D10 | P5 | 3 | 2 | 1 |
| M1 | ML- | D10 | P5 | 4 | 2 | 0 |
| M1 | ML- | D10 | P6 | 1 | 2 | 1 |
| M1 | ML- | D10 | P6 | 2 | 2 | 0 |
| M1 | ML- | D10 | P6 | 3 | 2 | 0 |
| M1 | ML- | D10 | P6 | 4 | 2 | 0 |

| | | | | | | |
|---|---|---|---|---|---|---|
| M1 | ML+ | D10 | P1 | 1 | 8 | 0 |
| M1 | ML+ | D10 | P1 | 2 | 8 | 0 |
| M1 | ML+ | D10 | P1 | 3 | 8 | 0 |
| M1 | ML+ | D10 | P1 | 4 | 8 | 0 |
| M1 | ML+ | D10 | P2 | 1 | 8 | 0 |
| M1 | ML+ | D10 | P2 | 2 | 8 | 0 |
| M1 | ML+ | D10 | P2 | 3 | 8 | 0 |
| M1 | ML+ | D10 | P2 | 4 | 8 | 0 |
| M1 | ML+ | D10 | P3 | 1 | 8 | 0 |
| M1 | ML+ | D10 | P3 | 2 | 8 | 0 |
| M1 | ML+ | D10 | P3 | 3 | 8 | 0 |
| M1 | ML+ | D10 | P3 | 4 | 8 | 0 |
| M1 | ML+ | D10 | P4 | 1 | 8 | 0 |
| M1 | ML+ | D10 | P4 | 2 | 8 | 0 |
| M1 | ML+ | D10 | P4 | 3 | 8 | 0 |
| M1 | ML+ | D10 | P4 | 4 | 8 | 0 |
| M1 | ML+ | D10 | P5 | 1 | 8 | 1 |
| M1 | ML+ | D10 | P5 | 2 | 8 | 0 |
| M1 | ML+ | D10 | P5 | 3 | 8 | 0 |
| M1 | ML+ | D10 | P5 | 4 | 8 | 0 |
| M1 | ML+ | D10 | P6 | 1 | 8 | 0 |
| M1 | ML+ | D10 | P6 | 2 | 8 | 0 |
| M1 | ML+ | D10 | P6 | 3 | 8 | 0 |
| M1 | ML+ | D10 | P6 | 4 | 8 | 0 |
| M1 | ML+ | D10 | P7 | 1 | 8 | 1 |
| M1 | ML+ | D10 | P7 | 2 | 8 | 0 |
| M1 | ML+ | D10 | P7 | 3 | 8 | 0 |
| M1 | ML+ | D10 | P7 | 4 | 8 | 0 |
| M1 | ML+ | D10 | P8 | 1 | 8 | 1 |
| M1 | ML+ | D10 | P8 | 2 | 8 | 0 |
| M1 | ML+ | D10 | P8 | 3 | 8 | 0 |
| M1 | ML+ | D10 | P8 | 4 | 8 | 0 |
| M1 | ML+ | D10 | P9 | 1 | 8 | 0 |
| M1 | ML+ | D10 | P9 | 2 | 8 | 0 |
| M1 | ML+ | D10 | P9 | 3 | 8 | 0 |
| M1 | ML+ | D10 | P9 | 4 | 8 | 0 |
| M1 | ML+ | D10 | P10 | 1 | 8 | 0 |
| M1 | ML+ | D10 | P10 | 2 | 8 | 0 |
| M1 | ML+ | D10 | P10 | 3 | 8 | 0 |
| M1 | ML+ | D10 | P10 | 4 | 8 | 0 |
| M1 | ML+ | D10 | P11 | 1 | 8 | 0 |
| M1 | ML+ | D10 | P11 | 2 | 8 | 0 |
| M1 | ML+ | D10 | P11 | 3 | 8 | 0 |
| M1 | ML+ | D10 | P11 | 4 | 8 | 0 |
| M1 | ML+ | D10 | P12 | 1 | 8 | 0 |
| M1 | ML+ | D10 | P12 | 2 | 8 | 0 |
| M1 | ML+ | D10 | P12 | 3 | 8 | 0 |
| M1 | ML+ | D10 | P12 | 4 | 8 | 0 |
| M1 | ML+ | D10 | P13 | 1 | 8 | 0 |
| M1 | ML+ | D10 | P13 | 2 | 8 | 0 |

| | | | | | | |
|---|---|---|---|---|---|---|
| M1 | ML+ | D10 | P13 | 3 | 8 | 0 |
| M1 | ML+ | D10 | P13 | 4 | 8 | 0 |
| M1 | ML+ | D10 | P14 | 1 | 8 | 0 |
| M1 | ML+ | D10 | P14 | 2 | 8 | 1 |
| M1 | ML+ | D10 | P14 | 3 | 8 | 0 |
| M1 | ML+ | D10 | P14 | 4 | 8 | 0 |
| M1 | ML+ | D10 | P15 | 1 | 8 | 0 |
| M1 | ML+ | D10 | P15 | 2 | 8 | 0 |
| M1 | ML+ | D10 | P15 | 3 | 8 | 0 |
| M1 | ML+ | D10 | P15 | 4 | 8 | 0 |
| M1 | ML+ | D10 | P16 | 1 | 8 | 0 |
| M1 | ML+ | D10 | P16 | 2 | 8 | 0 |
| M1 | ML+ | D10 | P16 | 3 | 8 | 0 |
| M1 | ML+ | D10 | P16 | 4 | 8 | 0 |
| M1 | ML- | D10 | P1 | 1 | 8 | 1 |
| M1 | ML- | D10 | P1 | 2 | 8 | 1 |
| M1 | ML- | D10 | P1 | 3 | 8 | 1 |
| M1 | ML- | D10 | P1 | 4 | 8 | 1 |
| M1 | ML- | D10 | P2 | 1 | 8 | 1 |
| M1 | ML- | D10 | P2 | 2 | 8 | 1 |
| M1 | ML- | D10 | P2 | 3 | 8 | 1 |
| M1 | ML- | D10 | P2 | 4 | 8 | 1 |
| M1 | ML- | D10 | P3 | 1 | 8 | 1 |
| M1 | ML- | D10 | P3 | 2 | 8 | 1 |
| M1 | ML- | D10 | P3 | 3 | 8 | 0 |
| M1 | ML- | D10 | P3 | 4 | 8 | 0 |
| M1 | ML- | D10 | P4 | 1 | 8 | 0 |
| M1 | ML- | D10 | P4 | 2 | 8 | 0 |
| M1 | ML- | D10 | P4 | 3 | 8 | 1 |
| M1 | ML- | D10 | P4 | 4 | 8 | 1 |
| M1 | ML- | D10 | P5 | 1 | 8 | 1 |
| M1 | ML- | D10 | P5 | 2 | 8 | 1 |
| M1 | ML- | D10 | P5 | 3 | 8 | 1 |
| M1 | ML- | D10 | P5 | 4 | 8 | 0 |
| M1 | ML- | D10 | P6 | 1 | 8 | 1 |
| M1 | ML- | D10 | P6 | 2 | 8 | 1 |
| M1 | ML- | D10 | P6 | 3 | 8 | 0 |
| M1 | ML- | D10 | P6 | 4 | 8 | 0 |
| M1 | ML+ | D10 | P1 | 1 | 16 | 0 |
| M1 | ML+ | D10 | P1 | 2 | 16 | 0 |
| M1 | ML+ | D10 | P1 | 3 | 16 | 0 |
| M1 | ML+ | D10 | P1 | 4 | 16 | 0 |
| M1 | ML+ | D10 | P2 | 1 | 16 | 0 |
| M1 | ML+ | D10 | P2 | 2 | 16 | 0 |
| M1 | ML+ | D10 | P2 | 3 | 16 | 0 |
| M1 | ML+ | D10 | P2 | 4 | 16 | 0 |
| M1 | ML+ | D10 | P3 | 1 | 16 | 0 |
| M1 | ML+ | D10 | P3 | 2 | 16 | 0 |
| M1 | ML+ | D10 | P3 | 3 | 16 | 0 |
| M1 | ML+ | D10 | P3 | 4 | 16 | 0 |

| | | | | | | |
|---|---|---|---|---|---|---|
| M1 | ML+ | D10 | P4 | 1 | 16 | 0 |
| M1 | ML+ | D10 | P4 | 2 | 16 | 0 |
| M1 | ML+ | D10 | P4 | 3 | 16 | 0 |
| M1 | ML+ | D10 | P4 | 4 | 16 | 0 |
| M1 | ML+ | D10 | P5 | 1 | 16 | 0 |
| M1 | ML+ | D10 | P5 | 2 | 16 | 0 |
| M1 | ML+ | D10 | P5 | 3 | 16 | 0 |
| M1 | ML+ | D10 | P5 | 4 | 16 | 0 |
| M1 | ML+ | D10 | P6 | 1 | 16 | 0 |
| M1 | ML+ | D10 | P6 | 2 | 16 | 0 |
| M1 | ML+ | D10 | P6 | 3 | 16 | 0 |
| M1 | ML+ | D10 | P6 | 4 | 16 | 0 |
| M1 | ML+ | D10 | P7 | 1 | 16 | 1 |
| M1 | ML+ | D10 | P7 | 2 | 16 | 0 |
| M1 | ML+ | D10 | P7 | 3 | 16 | 0 |
| M1 | ML+ | D10 | P7 | 4 | 16 | 0 |
| M1 | ML+ | D10 | P8 | 1 | 16 | 1 |
| M1 | ML+ | D10 | P8 | 2 | 16 | 0 |
| M1 | ML+ | D10 | P8 | 3 | 16 | 0 |
| M1 | ML+ | D10 | P8 | 4 | 16 | 0 |
| M1 | ML+ | D10 | P9 | 1 | 16 | 0 |
| M1 | ML+ | D10 | P9 | 2 | 16 | 0 |
| M1 | ML+ | D10 | P9 | 3 | 16 | 0 |
| M1 | ML+ | D10 | P9 | 4 | 16 | 0 |
| M1 | ML+ | D10 | P10 | 1 | 16 | 0 |
| M1 | ML+ | D10 | P10 | 2 | 16 | 0 |
| M1 | ML+ | D10 | P10 | 3 | 16 | 0 |
| M1 | ML+ | D10 | P10 | 4 | 16 | 0 |
| M1 | ML+ | D10 | P11 | 1 | 16 | 0 |
| M1 | ML+ | D10 | P11 | 2 | 16 | 0 |
| M1 | ML+ | D10 | P11 | 3 | 16 | 0 |
| M1 | ML+ | D10 | P11 | 4 | 16 | 0 |
| M1 | ML+ | D10 | P12 | 1 | 16 | 0 |
| M1 | ML+ | D10 | P12 | 2 | 16 | 0 |
| M1 | ML+ | D10 | P12 | 3 | 16 | 0 |
| M1 | ML+ | D10 | P12 | 4 | 16 | 0 |
| M1 | ML+ | D10 | P13 | 1 | 16 | 0 |
| M1 | ML+ | D10 | P13 | 2 | 16 | 1 |
| M1 | ML+ | D10 | P13 | 3 | 16 | 0 |
| M1 | ML+ | D10 | P13 | 4 | 16 | 0 |
| M1 | ML+ | D10 | P14 | 1 | 16 | 0 |
| M1 | ML+ | D10 | P14 | 2 | 16 | 0 |
| M1 | ML+ | D10 | P14 | 3 | 16 | 0 |
| M1 | ML+ | D10 | P14 | 4 | 16 | 0 |
| M1 | ML+ | D10 | P15 | 1 | 16 | 0 |
| M1 | ML+ | D10 | P15 | 2 | 16 | 0 |
| M1 | ML+ | D10 | P15 | 3 | 16 | 0 |
| M1 | ML+ | D10 | P15 | 4 | 16 | 0 |
| M1 | ML+ | D10 | P16 | 1 | 16 | 0 |
| M1 | ML+ | D10 | P16 | 2 | 16 | 0 |

| | | | | | | |
|---|---|---|---|---|---|---|
| M1 | ML+ | D10 | P16 | 3 | 16 | 0 |
| M1 | ML+ | D10 | P16 | 4 | 16 | 0 |
| M1 | ML- | D10 | P1 | 1 | 16 | 1 |
| M1 | ML- | D10 | P1 | 2 | 16 | 1 |
| M1 | ML- | D10 | P1 | 3 | 16 | 1 |
| M1 | ML- | D10 | P1 | 4 | 16 | 1 |
| M1 | ML- | D10 | P2 | 1 | 16 | 1 |
| M1 | ML- | D10 | P2 | 2 | 16 | 1 |
| M1 | ML- | D10 | P2 | 3 | 16 | 1 |
| M1 | ML- | D10 | P2 | 4 | 16 | 1 |
| M1 | ML- | D10 | P3 | 1 | 16 | 0 |
| M1 | ML- | D10 | P3 | 2 | 16 | 0 |
| M1 | ML- | D10 | P3 | 3 | 16 | 0 |
| M1 | ML- | D10 | P3 | 4 | 16 | 0 |
| M1 | ML- | D10 | P4 | 1 | 16 | 1 |
| M1 | ML- | D10 | P4 | 2 | 16 | 1 |
| M1 | ML- | D10 | P4 | 3 | 16 | 1 |
| M1 | ML- | D10 | P4 | 4 | 16 | 1 |
| M1 | ML- | D10 | P5 | 1 | 16 | 1 |
| M1 | ML- | D10 | P5 | 2 | 16 | 1 |
| M1 | ML- | D10 | P5 | 3 | 16 | 1 |
| M1 | ML- | D10 | P5 | 4 | 16 | 0 |
| M1 | ML- | D10 | P6 | 1 | 16 | 1 |
| M1 | ML- | D10 | P6 | 2 | 16 | 1 |
| M1 | ML- | D10 | P6 | 3 | 16 | 1 |
| M1 | ML- | D10 | P6 | 4 | 16 | 0 |
| M1 | ML- | D1 | P1 | 1 | 2 | 0 |
| M1 | ML- | D1 | P1 | 2 | 2 | 0 |
| M1 | ML- | D1 | P1 | 3 | 2 | 0 |
| M1 | ML- | D1 | P1 | 4 | 2 | 0 |
| M1 | ML- | D1 | P2 | 1 | 2 | 0 |
| M1 | ML- | D1 | P2 | 2 | 2 | 0 |
| M1 | ML- | D1 | P2 | 3 | 2 | 0 |
| M1 | ML- | D1 | P2 | 4 | 2 | 0 |
| M1 | ML- | D1 | P3 | 1 | 2 | 0 |
| M1 | ML- | D1 | P3 | 2 | 2 | 0 |
| M1 | ML- | D1 | P3 | 3 | 2 | 0 |
| M1 | ML- | D1 | P3 | 4 | 2 | 0 |
| M1 | ML- | D1 | P4 | 1 | 2 | 0 |
| M1 | ML- | D1 | P4 | 2 | 2 | 0 |
| M1 | ML- | D1 | P4 | 3 | 2 | 0 |
| M1 | ML- | D1 | P4 | 4 | 2 | 0 |
| M1 | ML- | D1 | P5 | 1 | 2 | 0 |
| M1 | ML- | D1 | P5 | 2 | 2 | 0 |
| M1 | ML- | D1 | P5 | 3 | 2 | 0 |
| M1 | ML- | D1 | P5 | 4 | 2 | 0 |
| M1 | ML- | D1 | P6 | 1 | 2 | 0 |
| M1 | ML- | D1 | P6 | 2 | 2 | 0 |
| M1 | ML- | D1 | P6 | 3 | 2 | 0 |
| M1 | ML- | D1 | P6 | 4 | 2 | 0 |

| | | | | | | |
|---|---|---|---|---|---|---|
| M1 | ML- | D1 | P7 | 1 | 2 | 0 |
| M1 | ML- | D1 | P7 | 2 | 2 | 0 |
| M1 | ML- | D1 | P7 | 3 | 2 | 0 |
| M1 | ML- | D1 | P7 | 4 | 2 | 0 |
| M1 | ML- | D1 | P8 | 1 | 2 | 0 |
| M1 | ML- | D1 | P8 | 2 | 2 | 0 |
| M1 | ML- | D1 | P8 | 3 | 2 | 0 |
| M1 | ML- | D1 | P8 | 4 | 2 | 0 |
| M1 | ML- | D1 | P9 | 1 | 2 | 0 |
| M1 | ML- | D1 | P9 | 2 | 2 | 0 |
| M1 | ML- | D1 | P9 | 3 | 2 | 0 |
| M1 | ML- | D1 | P9 | 4 | 2 | 0 |
| M1 | ML- | D1 | P10 | 1 | 2 | 0 |
| M1 | ML- | D1 | P10 | 2 | 2 | 1 |
| M1 | ML- | D1 | P10 | 3 | 2 | 0 |
| M1 | ML- | D1 | P10 | 4 | 2 | 0 |
| M1 | ML- | D1 | P11 | 1 | 2 | 0 |
| M1 | ML- | D1 | P11 | 2 | 2 | 0 |
| M1 | ML- | D1 | P11 | 3 | 2 | 0 |
| M1 | ML- | D1 | P11 | 4 | 2 | 0 |
| M1 | ML- | D1 | P12 | 1 | 2 | 0 |
| M1 | ML- | D1 | P12 | 2 | 2 | 1 |
| M1 | ML- | D1 | P12 | 3 | 2 | 0 |
| M1 | ML- | D1 | P12 | 4 | 2 | 0 |
| M1 | ML- | D1 | P13 | 1 | 2 | 0 |
| M1 | ML- | D1 | P13 | 2 | 2 | 0 |
| M1 | ML- | D1 | P13 | 3 | 2 | 0 |
| M1 | ML- | D1 | P13 | 4 | 2 | 0 |
| M1 | ML- | D1 | P14 | 1 | 2 | 0 |
| M1 | ML- | D1 | P14 | 2 | 2 | 0 |
| M1 | ML- | D1 | P14 | 3 | 2 | 0 |
| M1 | ML- | D1 | P14 | 4 | 2 | 0 |
| M1 | ML- | D1 | P15 | 1 | 2 | 0 |
| M1 | ML- | D1 | P15 | 2 | 2 | 0 |
| M1 | ML- | D1 | P15 | 3 | 2 | 0 |
| M1 | ML- | D1 | P15 | 4 | 2 | 0 |
| M1 | ML- | D1 | P16 | 1 | 2 | 0 |
| M1 | ML- | D1 | P16 | 2 | 2 | 0 |
| M1 | ML- | D1 | P16 | 3 | 2 | 0 |
| M1 | ML- | D1 | P16 | 4 | 2 | 0 |
| M1 | ML- | D1 | P17 | 1 | 2 | 0 |
| M1 | ML- | D1 | P17 | 2 | 2 | 0 |
| M1 | ML- | D1 | P17 | 3 | 2 | 0 |
| M1 | ML- | D1 | P17 | 4 | 2 | 0 |
| M1 | ML- | D1 | P18 | 1 | 2 | 0 |
| M1 | ML- | D1 | P18 | 2 | 2 | 0 |
| M1 | ML- | D1 | P18 | 3 | 2 | 0 |
| M1 | ML- | D1 | P18 | 4 | 2 | 0 |
| M1 | ML- | D1 | P19 | 1 | 2 | 0 |
| M1 | ML- | D1 | P19 | 2 | 2 | 0 |

| | | | | | | |
|---|---|---|---|---|---|---|
| M1 | ML- | D1 | P19 | 3 | 2 | 0 |
| M1 | ML- | D1 | P19 | 4 | 2 | 0 |
| M1 | ML- | D1 | P20 | 1 | 2 | 1 |
| M1 | ML- | D1 | P20 | 2 | 2 | 0 |
| M1 | ML- | D1 | P20 | 3 | 2 | 0 |
| M1 | ML- | D1 | P20 | 4 | 2 | 0 |
| M1 | ML- | D1 | P21 | 1 | 2 | 0 |
| M1 | ML- | D1 | P21 | 2 | 2 | 0 |
| M1 | ML- | D1 | P21 | 3 | 2 | 0 |
| M1 | ML- | D1 | P21 | 4 | 2 | 0 |
| M1 | ML- | D1 | P22 | 1 | 2 | 0 |
| M1 | ML- | D1 | P22 | 2 | 2 | 0 |
| M1 | ML- | D1 | P22 | 3 | 2 | 0 |
| M1 | ML- | D1 | P22 | 4 | 2 | 0 |
| M1 | ML- | D1 | P23 | 1 | 2 | 1 |
| M1 | ML- | D1 | P23 | 2 | 2 | 0 |
| M1 | ML- | D1 | P23 | 3 | 2 | 0 |
| M1 | ML- | D1 | P23 | 4 | 2 | 0 |
| M1 | ML- | D1 | P24 | 1 | 2 | 0 |
| M1 | ML- | D1 | P24 | 2 | 2 | 1 |
| M1 | ML- | D1 | P24 | 3 | 2 | 0 |
| M1 | ML- | D1 | P24 | 4 | 2 | 0 |
| M1 | ML+ | D1 | P1 | 1 | 2 | 0 |
| M1 | ML+ | D1 | P1 | 2 | 2 | 0 |
| M1 | ML+ | D1 | P1 | 3 | 2 | 0 |
| M1 | ML+ | D1 | P1 | 4 | 2 | 0 |
| M1 | ML+ | D1 | P2 | 1 | 2 | 0 |
| M1 | ML+ | D1 | P2 | 2 | 2 | 0 |
| M1 | ML+ | D1 | P2 | 3 | 2 | 0 |
| M1 | ML+ | D1 | P2 | 4 | 2 | 0 |
| M1 | ML+ | D1 | P3 | 1 | 2 | 0 |
| M1 | ML+ | D1 | P3 | 2 | 2 | 0 |
| M1 | ML+ | D1 | P3 | 3 | 2 | 0 |
| M1 | ML+ | D1 | P3 | 4 | 2 | 0 |
| M1 | ML+ | D1 | P4 | 1 | 2 | 0 |
| M1 | ML+ | D1 | P4 | 2 | 2 | 0 |
| M1 | ML+ | D1 | P4 | 3 | 2 | 0 |
| M1 | ML+ | D1 | P4 | 4 | 2 | 0 |
| M1 | ML+ | D1 | P5 | 1 | 2 | 0 |
| M1 | ML+ | D1 | P5 | 2 | 2 | 0 |
| M1 | ML+ | D1 | P5 | 3 | 2 | 0 |
| M1 | ML+ | D1 | P5 | 4 | 2 | 0 |
| M1 | ML+ | D1 | P6 | 1 | 2 | 0 |
| M1 | ML+ | D1 | P6 | 2 | 2 | 0 |
| M1 | ML+ | D1 | P6 | 3 | 2 | 0 |
| M1 | ML+ | D1 | P6 | 4 | 2 | 0 |
| M1 | ML+ | D1 | P7 | 1 | 2 | 0 |
| M1 | ML+ | D1 | P7 | 2 | 2 | 0 |
| M1 | ML+ | D1 | P7 | 3 | 2 | 0 |
| M1 | ML+ | D1 | P7 | 4 | 2 | 0 |

| | | | | | | |
|---|---|---|---|---|---|---|
| M1 | ML+ | D1 | P8 | 1 | 2 | 0 |
| M1 | ML+ | D1 | P8 | 2 | 2 | 0 |
| M1 | ML+ | D1 | P8 | 3 | 2 | 0 |
| M1 | ML+ | D1 | P8 | 4 | 2 | 0 |
| M1 | ML+ | D1 | P9 | 1 | 2 | 0 |
| M1 | ML+ | D1 | P9 | 2 | 2 | 0 |
| M1 | ML+ | D1 | P9 | 3 | 2 | 0 |
| M1 | ML+ | D1 | P9 | 4 | 2 | 0 |
| M1 | ML+ | D1 | P10 | 1 | 2 | 0 |
| M1 | ML+ | D1 | P10 | 2 | 2 | 0 |
| M1 | ML+ | D1 | P10 | 3 | 2 | 0 |
| M1 | ML+ | D1 | P10 | 4 | 2 | 0 |
| M1 | ML+ | D1 | P11 | 1 | 2 | 0 |
| M1 | ML+ | D1 | P11 | 2 | 2 | 0 |
| M1 | ML+ | D1 | P11 | 3 | 2 | 0 |
| M1 | ML+ | D1 | P11 | 4 | 2 | 0 |
| M1 | ML+ | D1 | P12 | 1 | 2 | 0 |
| M1 | ML+ | D1 | P12 | 2 | 2 | 0 |
| M1 | ML+ | D1 | P12 | 3 | 2 | 0 |
| M1 | ML+ | D1 | P12 | 4 | 2 | 0 |
| M1 | ML+ | D1 | P13 | 1 | 2 | 0 |
| M1 | ML+ | D1 | P13 | 2 | 2 | 0 |
| M1 | ML+ | D1 | P13 | 3 | 2 | 0 |
| M1 | ML+ | D1 | P13 | 4 | 2 | 0 |
| M1 | ML+ | D1 | P14 | 1 | 2 | 0 |
| M1 | ML+ | D1 | P14 | 2 | 2 | 0 |
| M1 | ML+ | D1 | P14 | 3 | 2 | 0 |
| M1 | ML+ | D1 | P14 | 4 | 2 | 0 |
| M1 | ML+ | D1 | P15 | 1 | 2 | 0 |
| M1 | ML+ | D1 | P15 | 2 | 2 | 0 |
| M1 | ML+ | D1 | P15 | 3 | 2 | 0 |
| M1 | ML+ | D1 | P15 | 4 | 2 | 0 |
| M1 | ML+ | D1 | P16 | 1 | 2 | 0 |
| M1 | ML+ | D1 | P16 | 2 | 2 | 0 |
| M1 | ML+ | D1 | P16 | 3 | 2 | 0 |
| M1 | ML+ | D1 | P16 | 4 | 2 | 0 |
| M1 | ML+ | D1 | P17 | 1 | 2 | 0 |
| M1 | ML+ | D1 | P17 | 2 | 2 | 0 |
| M1 | ML+ | D1 | P17 | 3 | 2 | 0 |
| M1 | ML+ | D1 | P17 | 4 | 2 | 0 |
| M1 | ML+ | D1 | P18 | 1 | 2 | 0 |
| M1 | ML+ | D1 | P18 | 2 | 2 | 0 |
| M1 | ML+ | D1 | P18 | 3 | 2 | 0 |
| M1 | ML+ | D1 | P18 | 4 | 2 | 0 |
| M1 | ML+ | D1 | P19 | 1 | 2 | 0 |
| M1 | ML+ | D1 | P19 | 2 | 2 | 0 |
| M1 | ML+ | D1 | P19 | 3 | 2 | 0 |
| M1 | ML+ | D1 | P19 | 4 | 2 | 0 |
| M1 | ML+ | D1 | P20 | 1 | 2 | 0 |
| M1 | ML+ | D1 | P20 | 2 | 2 | 0 |

| | | | | | | |
|---|---|---|---|---|---|---|
| M1 | ML+ | D1 | P20 | 3 | 2 | 0 |
| M1 | ML+ | D1 | P20 | 4 | 2 | 0 |
| M1 | ML+ | D1 | P21 | 1 | 2 | 0 |
| M1 | ML+ | D1 | P21 | 2 | 2 | 0 |
| M1 | ML+ | D1 | P21 | 3 | 2 | 0 |
| M1 | ML+ | D1 | P21 | 4 | 2 | 0 |
| M1 | ML+ | D1 | P22 | 1 | 2 | 0 |
| M1 | ML+ | D1 | P22 | 2 | 2 | 0 |
| M1 | ML+ | D1 | P22 | 3 | 2 | 0 |
| M1 | ML+ | D1 | P22 | 4 | 2 | 0 |
| M1 | ML+ | D1 | P23 | 1 | 2 | 0 |
| M1 | ML+ | D1 | P23 | 2 | 2 | 0 |
| M1 | ML+ | D1 | P23 | 3 | 2 | 0 |
| M1 | ML+ | D1 | P23 | 4 | 2 | 0 |
| M1 | ML+ | D1 | P24 | 1 | 2 | 0 |
| M1 | ML+ | D1 | P24 | 2 | 2 | 0 |
| M1 | ML+ | D1 | P24 | 3 | 2 | 0 |
| M1 | ML+ | D1 | P24 | 4 | 2 | 0 |
| M1 | ML- | D1 | P1 | 1 | 9 | 0 |
| M1 | ML- | D1 | P1 | 2 | 9 | 1 |
| M1 | ML- | D1 | P1 | 3 | 9 | 0 |
| M1 | ML- | D1 | P1 | 4 | 9 | 1 |
| M1 | ML- | D1 | P2 | 1 | 9 | 1 |
| M1 | ML- | D1 | P2 | 2 | 9 | 0 |
| M1 | ML- | D1 | P2 | 3 | 9 | 0 |
| M1 | ML- | D1 | P2 | 4 | 9 | 1 |
| M1 | ML- | D1 | P3 | 1 | 9 | 1 |
| M1 | ML- | D1 | P3 | 2 | 9 | 1 |
| M1 | ML- | D1 | P3 | 3 | 9 | 0 |
| M1 | ML- | D1 | P3 | 4 | 9 | 0 |
| M1 | ML- | D1 | P4 | 1 | 9 | 1 |
| M1 | ML- | D1 | P4 | 2 | 9 | 1 |
| M1 | ML- | D1 | P4 | 3 | 9 | 0 |
| M1 | ML- | D1 | P4 | 4 | 9 | 0 |
| M1 | ML- | D1 | P5 | 1 | 9 | 1 |
| M1 | ML- | D1 | P5 | 2 | 9 | 0 |
| M1 | ML- | D1 | P5 | 3 | 9 | 1 |
| M1 | ML- | D1 | P5 | 4 | 9 | 1 |
| M1 | ML- | D1 | P6 | 1 | 9 | 1 |
| M1 | ML- | D1 | P6 | 2 | 9 | 1 |
| M1 | ML- | D1 | P6 | 3 | 9 | 0 |
| M1 | ML- | D1 | P6 | 4 | 9 | 0 |
| M1 | ML- | D1 | P7 | 1 | 9 | 1 |
| M1 | ML- | D1 | P7 | 2 | 9 | 0 |
| M1 | ML- | D1 | P7 | 3 | 9 | 0 |
| M1 | ML- | D1 | P7 | 4 | 9 | 0 |
| M1 | ML- | D1 | P8 | 1 | 9 | 0 |
| M1 | ML- | D1 | P8 | 2 | 9 | 1 |
| M1 | ML- | D1 | P8 | 3 | 9 | 1 |
| M1 | ML- | D1 | P8 | 4 | 9 | 0 |

| | | | | | | |
|---|---|---|---|---|---|---|
| M1 | ML- | D1 | P9 | 1 | 9 | 1 |
| M1 | ML- | D1 | P9 | 2 | 9 | 1 |
| M1 | ML- | D1 | P9 | 3 | 9 | 1 |
| M1 | ML- | D1 | P9 | 4 | 9 | 0 |
| M1 | ML- | D1 | P10 | 1 | 9 | 0 |
| M1 | ML- | D1 | P10 | 2 | 9 | 1 |
| M1 | ML- | D1 | P10 | 3 | 9 | 1 |
| M1 | ML- | D1 | P10 | 4 | 9 | 0 |
| M1 | ML- | D1 | P11 | 1 | 9 | 1 |
| M1 | ML- | D1 | P11 | 2 | 9 | 0 |
| M1 | ML- | D1 | P11 | 3 | 9 | 1 |
| M1 | ML- | D1 | P11 | 4 | 9 | 1 |
| M1 | ML- | D1 | P12 | 1 | 9 | 1 |
| M1 | ML- | D1 | P12 | 2 | 9 | 1 |
| M1 | ML- | D1 | P12 | 3 | 9 | 1 |
| M1 | ML- | D1 | P12 | 4 | 9 | 0 |
| M1 | ML- | D1 | P13 | 1 | 9 | 1 |
| M1 | ML- | D1 | P13 | 2 | 9 | 0 |
| M1 | ML- | D1 | P13 | 3 | 9 | 1 |
| M1 | ML- | D1 | P13 | 4 | 9 | 1 |
| M1 | ML- | D1 | P14 | 1 | 9 | 0 |
| M1 | ML- | D1 | P14 | 2 | 9 | 0 |
| M1 | ML- | D1 | P14 | 3 | 9 | 0 |
| M1 | ML- | D1 | P14 | 4 | 9 | 0 |
| M1 | ML- | D1 | P15 | 1 | 9 | 1 |
| M1 | ML- | D1 | P15 | 2 | 9 | 0 |
| M1 | ML- | D1 | P15 | 3 | 9 | 1 |
| M1 | ML- | D1 | P15 | 4 | 9 | 0 |
| M1 | ML- | D1 | P16 | 1 | 9 | 0 |
| M1 | ML- | D1 | P16 | 2 | 9 | 0 |
| M1 | ML- | D1 | P16 | 3 | 9 | 1 |
| M1 | ML- | D1 | P16 | 4 | 9 | 0 |
| M1 | ML- | D1 | P17 | 1 | 9 | 1 |
| M1 | ML- | D1 | P17 | 2 | 9 | 0 |
| M1 | ML- | D1 | P17 | 3 | 9 | 0 |
| M1 | ML- | D1 | P17 | 4 | 9 | 1 |
| M1 | ML- | D1 | P18 | 1 | 9 | 0 |
| M1 | ML- | D1 | P18 | 2 | 9 | 1 |
| M1 | ML- | D1 | P18 | 3 | 9 | 0 |
| M1 | ML- | D1 | P18 | 4 | 9 | 0 |
| M1 | ML- | D1 | P19 | 1 | 9 | 0 |
| M1 | ML- | D1 | P19 | 2 | 9 | 0 |
| M1 | ML- | D1 | P19 | 3 | 9 | 0 |
| M1 | ML- | D1 | P19 | 4 | 9 | 0 |
| M1 | ML- | D1 | P20 | 1 | 9 | 1 |
| M1 | ML- | D1 | P20 | 2 | 9 | 0 |
| M1 | ML- | D1 | P20 | 3 | 9 | 0 |
| M1 | ML- | D1 | P20 | 4 | 9 | 0 |
| M1 | ML- | D1 | P21 | 1 | 9 | 1 |
| M1 | ML- | D1 | P21 | 2 | 9 | 1 |

| | | | | | | |
|---|---|---|---|---|---|---|
| M1 | ML- | D1 | P21 | 3 | 9 | 0 |
| M1 | ML- | D1 | P21 | 4 | 9 | 0 |
| M1 | ML- | D1 | P22 | 1 | 9 | 0 |
| M1 | ML- | D1 | P22 | 2 | 9 | 0 |
| M1 | ML- | D1 | P22 | 3 | 9 | 0 |
| M1 | ML- | D1 | P22 | 4 | 9 | 0 |
| M1 | ML- | D1 | P23 | 1 | 9 | 1 |
| M1 | ML- | D1 | P23 | 2 | 9 | 0 |
| M1 | ML- | D1 | P23 | 3 | 9 | 0 |
| M1 | ML- | D1 | P23 | 4 | 9 | 0 |
| M1 | ML- | D1 | P24 | 1 | 9 | 1 |
| M1 | ML- | D1 | P24 | 2 | 9 | 1 |
| M1 | ML- | D1 | P24 | 3 | 9 | 0 |
| M1 | ML- | D1 | P24 | 4 | 9 | 1 |
| M1 | ML+ | D1 | P1 | 1 | 9 | 0 |
| M1 | ML+ | D1 | P1 | 2 | 9 | 0 |
| M1 | ML+ | D1 | P1 | 3 | 9 | 0 |
| M1 | ML+ | D1 | P1 | 4 | 9 | 0 |
| M1 | ML+ | D1 | P2 | 1 | 9 | 0 |
| M1 | ML+ | D1 | P2 | 2 | 9 | 0 |
| M1 | ML+ | D1 | P2 | 3 | 9 | 0 |
| M1 | ML+ | D1 | P2 | 4 | 9 | 0 |
| M1 | ML+ | D1 | P3 | 1 | 9 | 0 |
| M1 | ML+ | D1 | P3 | 2 | 9 | 0 |
| M1 | ML+ | D1 | P3 | 3 | 9 | 0 |
| M1 | ML+ | D1 | P3 | 4 | 9 | 0 |
| M1 | ML+ | D1 | P4 | 1 | 9 | 0 |
| M1 | ML+ | D1 | P4 | 2 | 9 | 0 |
| M1 | ML+ | D1 | P4 | 3 | 9 | 0 |
| M1 | ML+ | D1 | P4 | 4 | 9 | 0 |
| M1 | ML+ | D1 | P5 | 1 | 9 | 0 |
| M1 | ML+ | D1 | P5 | 2 | 9 | 0 |
| M1 | ML+ | D1 | P5 | 3 | 9 | 0 |
| M1 | ML+ | D1 | P5 | 4 | 9 | 0 |
| M1 | ML+ | D1 | P6 | 1 | 9 | 0 |
| M1 | ML+ | D1 | P6 | 2 | 9 | 0 |
| M1 | ML+ | D1 | P6 | 3 | 9 | 0 |
| M1 | ML+ | D1 | P6 | 4 | 9 | 0 |
| M1 | ML+ | D1 | P7 | 1 | 9 | 0 |
| M1 | ML+ | D1 | P7 | 2 | 9 | 0 |
| M1 | ML+ | D1 | P7 | 3 | 9 | 0 |
| M1 | ML+ | D1 | P7 | 4 | 9 | 0 |
| M1 | ML+ | D1 | P8 | 1 | 9 | 0 |
| M1 | ML+ | D1 | P8 | 2 | 9 | 0 |
| M1 | ML+ | D1 | P8 | 3 | 9 | 0 |
| M1 | ML+ | D1 | P8 | 4 | 9 | 0 |
| M1 | ML+ | D1 | P9 | 1 | 9 | 0 |
| M1 | ML+ | D1 | P9 | 2 | 9 | 0 |
| M1 | ML+ | D1 | P9 | 3 | 9 | 0 |
| M1 | ML+ | D1 | P9 | 4 | 9 | 0 |

| | | | | | | |
|---|---|---|---|---|---|---|
| M1 | ML+ | D1 | P10 | 1 | 9 | 0 |
| M1 | ML+ | D1 | P10 | 2 | 9 | 0 |
| M1 | ML+ | D1 | P10 | 3 | 9 | 0 |
| M1 | ML+ | D1 | P10 | 4 | 9 | 0 |
| M1 | ML+ | D1 | P11 | 1 | 9 | 0 |
| M1 | ML+ | D1 | P11 | 2 | 9 | 0 |
| M1 | ML+ | D1 | P11 | 3 | 9 | 0 |
| M1 | ML+ | D1 | P11 | 4 | 9 | 0 |
| M1 | ML+ | D1 | P12 | 1 | 9 | 0 |
| M1 | ML+ | D1 | P12 | 2 | 9 | 0 |
| M1 | ML+ | D1 | P12 | 3 | 9 | 0 |
| M1 | ML+ | D1 | P12 | 4 | 9 | 0 |
| M1 | ML+ | D1 | P13 | 1 | 9 | 0 |
| M1 | ML+ | D1 | P13 | 2 | 9 | 0 |
| M1 | ML+ | D1 | P13 | 3 | 9 | 0 |
| M1 | ML+ | D1 | P13 | 4 | 9 | 0 |
| M1 | ML+ | D1 | P14 | 1 | 9 | 0 |
| M1 | ML+ | D1 | P14 | 2 | 9 | 0 |
| M1 | ML+ | D1 | P14 | 3 | 9 | 0 |
| M1 | ML+ | D1 | P14 | 4 | 9 | 0 |
| M1 | ML+ | D1 | P15 | 1 | 9 | 0 |
| M1 | ML+ | D1 | P15 | 2 | 9 | 0 |
| M1 | ML+ | D1 | P15 | 3 | 9 | 1 |
| M1 | ML+ | D1 | P15 | 4 | 9 | 0 |
| M1 | ML+ | D1 | P16 | 1 | 9 | 0 |
| M1 | ML+ | D1 | P16 | 2 | 9 | 0 |
| M1 | ML+ | D1 | P16 | 3 | 9 | 0 |
| M1 | ML+ | D1 | P16 | 4 | 9 | 0 |
| M1 | ML+ | D1 | P17 | 1 | 9 | 0 |
| M1 | ML+ | D1 | P17 | 2 | 9 | 0 |
| M1 | ML+ | D1 | P17 | 3 | 9 | 0 |
| M1 | ML+ | D1 | P17 | 4 | 9 | 0 |
| M1 | ML+ | D1 | P18 | 1 | 9 | 0 |
| M1 | ML+ | D1 | P18 | 2 | 9 | 0 |
| M1 | ML+ | D1 | P18 | 3 | 9 | 0 |
| M1 | ML+ | D1 | P18 | 4 | 9 | 0 |
| M1 | ML+ | D1 | P19 | 1 | 9 | 0 |
| M1 | ML+ | D1 | P19 | 2 | 9 | 0 |
| M1 | ML+ | D1 | P19 | 3 | 9 | 0 |
| M1 | ML+ | D1 | P19 | 4 | 9 | 0 |
| M1 | ML+ | D1 | P20 | 1 | 9 | 0 |
| M1 | ML+ | D1 | P20 | 2 | 9 | 0 |
| M1 | ML+ | D1 | P20 | 3 | 9 | 0 |
| M1 | ML+ | D1 | P20 | 4 | 9 | 0 |
| M1 | ML+ | D1 | P21 | 1 | 9 | 0 |
| M1 | ML+ | D1 | P21 | 2 | 9 | 0 |
| M1 | ML+ | D1 | P21 | 3 | 9 | 0 |
| M1 | ML+ | D1 | P21 | 4 | 9 | 0 |
| M1 | ML+ | D1 | P22 | 1 | 9 | 0 |
| M1 | ML+ | D1 | P22 | 2 | 9 | 0 |

| | | | | | | |
|---|---|---|---|---|---|---|
| M1 | ML+ | D1 | P22 | 3 | 9 | 0 |
| M1 | ML+ | D1 | P22 | 4 | 9 | 0 |
| M1 | ML+ | D1 | P23 | 1 | 9 | 0 |
| M1 | ML+ | D1 | P23 | 2 | 9 | 0 |
| M1 | ML+ | D1 | P23 | 3 | 9 | 0 |
| M1 | ML+ | D1 | P23 | 4 | 9 | 0 |
| M1 | ML+ | D1 | P24 | 1 | 9 | 0 |
| M1 | ML+ | D1 | P24 | 2 | 9 | 0 |
| M1 | ML+ | D1 | P24 | 3 | 9 | 0 |
| M1 | ML+ | D1 | P24 | 4 | 9 | 0 |
| M1 | ML- | D1 | P1 | 1 | 16 | 0 |
| M1 | ML- | D1 | P1 | 2 | 16 | 0 |
| M1 | ML- | D1 | P1 | 3 | 16 | 0 |
| M1 | ML- | D1 | P1 | 4 | 16 | 1 |
| M1 | ML- | D1 | P2 | 1 | 16 | 1 |
| M1 | ML- | D1 | P2 | 2 | 16 | 0 |
| M1 | ML- | D1 | P2 | 3 | 16 | 0 |
| M1 | ML- | D1 | P2 | 4 | 16 | 1 |
| M1 | ML- | D1 | P3 | 1 | 16 | 1 |
| M1 | ML- | D1 | P3 | 2 | 16 | 1 |
| M1 | ML- | D1 | P3 | 3 | 16 | 0 |
| M1 | ML- | D1 | P3 | 4 | 16 | 0 |
| M1 | ML- | D1 | P4 | 1 | 16 | 1 |
| M1 | ML- | D1 | P4 | 2 | 16 | 1 |
| M1 | ML- | D1 | P4 | 3 | 16 | 1 |
| M1 | ML- | D1 | P4 | 4 | 16 | 1 |
| M1 | ML- | D1 | P5 | 1 | 16 | 1 |
| M1 | ML- | D1 | P5 | 2 | 16 | 0 |
| M1 | ML- | D1 | P5 | 3 | 16 | 1 |
| M1 | ML- | D1 | P5 | 4 | 16 | 1 |
| M1 | ML- | D1 | P6 | 1 | 16 | 0 |
| M1 | ML- | D1 | P6 | 2 | 16 | 1 |
| M1 | ML- | D1 | P6 | 3 | 16 | 0 |
| M1 | ML- | D1 | P6 | 4 | 16 | 0 |
| M1 | ML- | D1 | P7 | 1 | 16 | 1 |
| M1 | ML- | D1 | P7 | 2 | 16 | 0 |
| M1 | ML- | D1 | P7 | 3 | 16 | 0 |
| M1 | ML- | D1 | P7 | 4 | 16 | 0 |
| M1 | ML- | D1 | P8 | 1 | 16 | 0 |
| M1 | ML- | D1 | P8 | 2 | 16 | 0 |
| M1 | ML- | D1 | P8 | 3 | 16 | 1 |
| M1 | ML- | D1 | P8 | 4 | 16 | 0 |
| M1 | ML- | D1 | P9 | 1 | 16 | 1 |
| M1 | ML- | D1 | P9 | 2 | 16 | 1 |
| M1 | ML- | D1 | P9 | 3 | 16 | 1 |
| M1 | ML- | D1 | P9 | 4 | 16 | 0 |
| M1 | ML- | D1 | P10 | 1 | 16 | 0 |
| M1 | ML- | D1 | P10 | 2 | 16 | 1 |
| M1 | ML- | D1 | P10 | 3 | 16 | 0 |
| M1 | ML- | D1 | P10 | 4 | 16 | 0 |

| | | | | | | |
|---|---|---|---|---|---|---|
| M1 | ML- | D1 | P11 | 1 | 16 | 1 |
| M1 | ML- | D1 | P11 | 2 | 16 | 0 |
| M1 | ML- | D1 | P11 | 3 | 16 | 1 |
| M1 | ML- | D1 | P11 | 4 | 16 | 1 |
| M1 | ML- | D1 | P12 | 1 | 16 | 1 |
| M1 | ML- | D1 | P12 | 2 | 16 | 1 |
| M1 | ML- | D1 | P12 | 3 | 16 | 1 |
| M1 | ML- | D1 | P12 | 4 | 16 | 0 |
| M1 | ML- | D1 | P13 | 1 | 16 | 1 |
| M1 | ML- | D1 | P13 | 2 | 16 | 0 |
| M1 | ML- | D1 | P13 | 3 | 16 | 1 |
| M1 | ML- | D1 | P13 | 4 | 16 | 1 |
| M1 | ML- | D1 | P14 | 1 | 16 | 1 |
| M1 | ML- | D1 | P14 | 2 | 16 | 0 |
| M1 | ML- | D1 | P14 | 3 | 16 | 0 |
| M1 | ML- | D1 | P14 | 4 | 16 | 0 |
| M1 | ML- | D1 | P15 | 1 | 16 | 1 |
| M1 | ML- | D1 | P15 | 2 | 16 | 0 |
| M1 | ML- | D1 | P15 | 3 | 16 | 0 |
| M1 | ML- | D1 | P15 | 4 | 16 | 0 |
| M1 | ML- | D1 | P16 | 1 | 16 | 0 |
| M1 | ML- | D1 | P16 | 2 | 16 | 0 |
| M1 | ML- | D1 | P16 | 3 | 16 | 1 |
| M1 | ML- | D1 | P16 | 4 | 16 | 0 |
| M1 | ML- | D1 | P17 | 1 | 16 | 1 |
| M1 | ML- | D1 | P17 | 2 | 16 | 0 |
| M1 | ML- | D1 | P17 | 3 | 16 | 0 |
| M1 | ML- | D1 | P17 | 4 | 16 | 0 |
| M1 | ML- | D1 | P18 | 1 | 16 | 0 |
| M1 | ML- | D1 | P18 | 2 | 16 | 1 |
| M1 | ML- | D1 | P18 | 3 | 16 | 0 |
| M1 | ML- | D1 | P18 | 4 | 16 | 0 |
| M1 | ML- | D1 | P19 | 1 | 16 | 0 |
| M1 | ML- | D1 | P19 | 2 | 16 | 0 |
| M1 | ML- | D1 | P19 | 3 | 16 | 0 |
| M1 | ML- | D1 | P19 | 4 | 16 | 0 |
| M1 | ML- | D1 | P20 | 1 | 16 | 1 |
| M1 | ML- | D1 | P20 | 2 | 16 | 0 |
| M1 | ML- | D1 | P20 | 3 | 16 | 0 |
| M1 | ML- | D1 | P20 | 4 | 16 | 0 |
| M1 | ML- | D1 | P21 | 1 | 16 | 1 |
| M1 | ML- | D1 | P21 | 2 | 16 | 1 |
| M1 | ML- | D1 | P21 | 3 | 16 | 1 |
| M1 | ML- | D1 | P21 | 4 | 16 | 1 |
| M1 | ML- | D1 | P22 | 1 | 16 | 0 |
| M1 | ML- | D1 | P22 | 2 | 16 | 0 |
| M1 | ML- | D1 | P22 | 3 | 16 | 0 |
| M1 | ML- | D1 | P22 | 4 | 16 | 0 |
| M1 | ML- | D1 | P23 | 1 | 16 | 1 |
| M1 | ML- | D1 | P23 | 2 | 16 | 0 |

| | | | | | | |
|---|---|---|---|---|---|---|
| M1 | ML- | D1 | P23 | 3 | 16 | 0 |
| M1 | ML- | D1 | P23 | 4 | 16 | 0 |
| M1 | ML- | D1 | P24 | 1 | 16 | 1 |
| M1 | ML- | D1 | P24 | 2 | 16 | 1 |
| M1 | ML- | D1 | P24 | 3 | 16 | 0 |
| M1 | ML- | D1 | P24 | 4 | 16 | 1 |
| M1 | ML+ | D1 | P1 | 1 | 16 | 0 |
| M1 | ML+ | D1 | P1 | 2 | 16 | 0 |
| M1 | ML+ | D1 | P1 | 3 | 16 | 0 |
| M1 | ML+ | D1 | P1 | 4 | 16 | 0 |
| M1 | ML+ | D1 | P2 | 1 | 16 | 0 |
| M1 | ML+ | D1 | P2 | 2 | 16 | 0 |
| M1 | ML+ | D1 | P2 | 3 | 16 | 0 |
| M1 | ML+ | D1 | P2 | 4 | 16 | 0 |
| M1 | ML+ | D1 | P3 | 1 | 16 | 0 |
| M1 | ML+ | D1 | P3 | 2 | 16 | 0 |
| M1 | ML+ | D1 | P3 | 3 | 16 | 0 |
| M1 | ML+ | D1 | P3 | 4 | 16 | 0 |
| M1 | ML+ | D1 | P4 | 1 | 16 | 0 |
| M1 | ML+ | D1 | P4 | 2 | 16 | 0 |
| M1 | ML+ | D1 | P4 | 3 | 16 | 0 |
| M1 | ML+ | D1 | P4 | 4 | 16 | 0 |
| M1 | ML+ | D1 | P5 | 1 | 16 | 0 |
| M1 | ML+ | D1 | P5 | 2 | 16 | 0 |
| M1 | ML+ | D1 | P5 | 3 | 16 | 0 |
| M1 | ML+ | D1 | P5 | 4 | 16 | 0 |
| M1 | ML+ | D1 | P6 | 1 | 16 | 0 |
| M1 | ML+ | D1 | P6 | 2 | 16 | 0 |
| M1 | ML+ | D1 | P6 | 3 | 16 | 0 |
| M1 | ML+ | D1 | P6 | 4 | 16 | 0 |
| M1 | ML+ | D1 | P7 | 1 | 16 | 0 |
| M1 | ML+ | D1 | P7 | 2 | 16 | 0 |
| M1 | ML+ | D1 | P7 | 3 | 16 | 0 |
| M1 | ML+ | D1 | P7 | 4 | 16 | 0 |
| M1 | ML+ | D1 | P8 | 1 | 16 | 0 |
| M1 | ML+ | D1 | P8 | 2 | 16 | 0 |
| M1 | ML+ | D1 | P8 | 3 | 16 | 0 |
| M1 | ML+ | D1 | P8 | 4 | 16 | 0 |
| M1 | ML+ | D1 | P9 | 1 | 16 | 0 |
| M1 | ML+ | D1 | P9 | 2 | 16 | 0 |
| M1 | ML+ | D1 | P9 | 3 | 16 | 0 |
| M1 | ML+ | D1 | P9 | 4 | 16 | 0 |
| M1 | ML+ | D1 | P10 | 1 | 16 | 0 |
| M1 | ML+ | D1 | P10 | 2 | 16 | 0 |
| M1 | ML+ | D1 | P10 | 3 | 16 | 0 |
| M1 | ML+ | D1 | P10 | 4 | 16 | 0 |
| M1 | ML+ | D1 | P11 | 1 | 16 | 0 |
| M1 | ML+ | D1 | P11 | 2 | 16 | 0 |
| M1 | ML+ | D1 | P11 | 3 | 16 | 0 |
| M1 | ML+ | D1 | P11 | 4 | 16 | 0 |

| | | | | | | |
|---|---|---|---|---|---|---|
| M1 | ML+ | D1 | P12 | 1 | 16 | 0 |
| M1 | ML+ | D1 | P12 | 2 | 16 | 0 |
| M1 | ML+ | D1 | P12 | 3 | 16 | 0 |
| M1 | ML+ | D1 | P12 | 4 | 16 | 0 |
| M1 | ML+ | D1 | P13 | 1 | 16 | 0 |
| M1 | ML+ | D1 | P13 | 2 | 16 | 0 |
| M1 | ML+ | D1 | P13 | 3 | 16 | 0 |
| M1 | ML+ | D1 | P13 | 4 | 16 | 0 |
| M1 | ML+ | D1 | P14 | 1 | 16 | 0 |
| M1 | ML+ | D1 | P14 | 2 | 16 | 0 |
| M1 | ML+ | D1 | P14 | 3 | 16 | 0 |
| M1 | ML+ | D1 | P14 | 4 | 16 | 0 |
| M1 | ML+ | D1 | P15 | 1 | 16 | 0 |
| M1 | ML+ | D1 | P15 | 2 | 16 | 0 |
| M1 | ML+ | D1 | P15 | 3 | 16 | 1 |
| M1 | ML+ | D1 | P15 | 4 | 16 | 0 |
| M1 | ML+ | D1 | P16 | 1 | 16 | 0 |
| M1 | ML+ | D1 | P16 | 2 | 16 | 0 |
| M1 | ML+ | D1 | P16 | 3 | 16 | 0 |
| M1 | ML+ | D1 | P16 | 4 | 16 | 0 |
| M1 | ML+ | D1 | P17 | 1 | 16 | 0 |
| M1 | ML+ | D1 | P17 | 2 | 16 | 0 |
| M1 | ML+ | D1 | P17 | 3 | 16 | 0 |
| M1 | ML+ | D1 | P17 | 4 | 16 | 0 |
| M1 | ML+ | D1 | P18 | 1 | 16 | 0 |
| M1 | ML+ | D1 | P18 | 2 | 16 | 0 |
| M1 | ML+ | D1 | P18 | 3 | 16 | 0 |
| M1 | ML+ | D1 | P18 | 4 | 16 | 0 |
| M1 | ML+ | D1 | P19 | 1 | 16 | 0 |
| M1 | ML+ | D1 | P19 | 2 | 16 | 0 |
| M1 | ML+ | D1 | P19 | 3 | 16 | 0 |
| M1 | ML+ | D1 | P19 | 4 | 16 | 0 |
| M1 | ML+ | D1 | P20 | 1 | 16 | 0 |
| M1 | ML+ | D1 | P20 | 2 | 16 | 0 |
| M1 | ML+ | D1 | P20 | 3 | 16 | 0 |
| M1 | ML+ | D1 | P20 | 4 | 16 | 0 |
| M1 | ML+ | D1 | P21 | 1 | 16 | 0 |
| M1 | ML+ | D1 | P21 | 2 | 16 | 0 |
| M1 | ML+ | D1 | P21 | 3 | 16 | 0 |
| M1 | ML+ | D1 | P21 | 4 | 16 | 0 |
| M1 | ML+ | D1 | P22 | 1 | 16 | 0 |
| M1 | ML+ | D1 | P22 | 2 | 16 | 0 |
| M1 | ML+ | D1 | P22 | 3 | 16 | 0 |
| M1 | ML+ | D1 | P22 | 4 | 16 | 0 |
| M1 | ML+ | D1 | P23 | 1 | 16 | 0 |
| M1 | ML+ | D1 | P23 | 2 | 16 | 0 |
| M1 | ML+ | D1 | P23 | 3 | 16 | 0 |
| M1 | ML+ | D1 | P23 | 4 | 16 | 0 |
| M1 | ML+ | D1 | P24 | 1 | 16 | 0 |
| M1 | ML+ | D1 | P24 | 2 | 16 | 0 |

| | | | | | | |
|---|---|---|---|---|---|---|
| M1 | ML+ | D1 | P24 | 3 | 16 | 0 |
| M1 | ML+ | D1 | P24 | 4 | 16 | 0 |
| M2 | ML- | D1 | P1 | 1 | 2 | 0 |
| M2 | ML- | D1 | P1 | 2 | 2 | 0 |
| M2 | ML- | D1 | P1 | 3 | 2 | 0 |
| M2 | ML- | D1 | P1 | 4 | 2 | 0 |
| M2 | ML- | D1 | P2 | 1 | 2 | 0 |
| M2 | ML- | D1 | P2 | 2 | 2 | 0 |
| M2 | ML- | D1 | P2 | 3 | 2 | 0 |
| M2 | ML- | D1 | P2 | 4 | 2 | 0 |
| M2 | ML- | D1 | P3 | 1 | 2 | 0 |
| M2 | ML- | D1 | P3 | 2 | 2 | 0 |
| M2 | ML- | D1 | P3 | 3 | 2 | 0 |
| M2 | ML- | D1 | P3 | 4 | 2 | 0 |
| M2 | ML- | D1 | P4 | 1 | 2 | 0 |
| M2 | ML- | D1 | P4 | 2 | 2 | 0 |
| M2 | ML- | D1 | P4 | 3 | 2 | 0 |
| M2 | ML- | D1 | P4 | 4 | 2 | 0 |
| M2 | ML- | D1 | P5 | 1 | 2 | 0 |
| M2 | ML- | D1 | P5 | 2 | 2 | 0 |
| M2 | ML- | D1 | P5 | 3 | 2 | 0 |
| M2 | ML- | D1 | P5 | 4 | 2 | 0 |
| M2 | ML- | D1 | P6 | 1 | 2 | 0 |
| M2 | ML- | D1 | P6 | 2 | 2 | 0 |
| M2 | ML- | D1 | P6 | 3 | 2 | 0 |
| M2 | ML- | D1 | P6 | 4 | 2 | 0 |
| M2 | ML- | D1 | P7 | 1 | 2 | 0 |
| M2 | ML- | D1 | P7 | 2 | 2 | 0 |
| M2 | ML- | D1 | P7 | 3 | 2 | 0 |
| M2 | ML- | D1 | P7 | 4 | 2 | 0 |
| M2 | ML- | D1 | P8 | 1 | 2 | 0 |
| M2 | ML- | D1 | P8 | 2 | 2 | 0 |
| M2 | ML- | D1 | P8 | 3 | 2 | 0 |
| M2 | ML- | D1 | P8 | 4 | 2 | 0 |
| M2 | ML- | D1 | P9 | 1 | 2 | 0 |
| M2 | ML- | D1 | P9 | 2 | 2 | 0 |
| M2 | ML- | D1 | P9 | 3 | 2 | 0 |
| M2 | ML- | D1 | P9 | 4 | 2 | 0 |
| M2 | ML- | D1 | P10 | 1 | 2 | 0 |
| M2 | ML- | D1 | P10 | 2 | 2 | 0 |
| M2 | ML- | D1 | P10 | 3 | 2 | 0 |
| M2 | ML- | D1 | P10 | 4 | 2 | 0 |
| M2 | ML- | D1 | P11 | 1 | 2 | 0 |
| M2 | ML- | D1 | P11 | 2 | 2 | 0 |
| M2 | ML- | D1 | P11 | 3 | 2 | 0 |
| M2 | ML- | D1 | P11 | 4 | 2 | 0 |
| M2 | ML- | D1 | P12 | 1 | 2 | 0 |
| M2 | ML- | D1 | P12 | 2 | 2 | 0 |
| M2 | ML- | D1 | P12 | 3 | 2 | 0 |
| M2 | ML- | D1 | P12 | 4 | 2 | 0 |

| | | | | | | |
|---|---|---|---|---|---|---|
| M2 | ML- | D1 | P13 | 1 | 2 | 0 |
| M2 | ML- | D1 | P13 | 2 | 2 | 0 |
| M2 | ML- | D1 | P13 | 3 | 2 | 0 |
| M2 | ML- | D1 | P13 | 4 | 2 | 0 |
| M2 | ML- | D1 | P14 | 1 | 2 | 0 |
| M2 | ML- | D1 | P14 | 2 | 2 | 0 |
| M2 | ML- | D1 | P14 | 3 | 2 | 0 |
| M2 | ML- | D1 | P14 | 4 | 2 | 0 |
| M2 | ML- | D1 | P15 | 1 | 2 | 0 |
| M2 | ML- | D1 | P15 | 2 | 2 | 0 |
| M2 | ML- | D1 | P15 | 3 | 2 | 0 |
| M2 | ML- | D1 | P15 | 4 | 2 | 0 |
| M2 | ML- | D1 | P16 | 1 | 2 | 0 |
| M2 | ML- | D1 | P16 | 2 | 2 | 0 |
| M2 | ML- | D1 | P16 | 3 | 2 na | |
| M2 | ML- | D1 | P16 | 4 | 2 | 0 |
| M2 | ML- | D1 | P17 | 1 | 2 | 0 |
| M2 | ML- | D1 | P17 | 2 | 2 | 0 |
| M2 | ML- | D1 | P17 | 3 | 2 | 0 |
| M2 | ML- | D1 | P17 | 4 | 2 | 0 |
| M2 | ML- | D1 | P18 | 1 | 2 | 0 |
| M2 | ML- | D1 | P18 | 2 | 2 | 0 |
| M2 | ML- | D1 | P18 | 3 | 2 | 0 |
| M2 | ML- | D1 | P18 | 4 | 2 | 0 |
| M2 | ML- | D1 | P19 | 1 | 2 | 0 |
| M2 | ML- | D1 | P19 | 2 | 2 | 0 |
| M2 | ML- | D1 | P19 | 3 | 2 | 0 |
| M2 | ML- | D1 | P19 | 4 | 2 | 0 |
| M2 | ML- | D1 | P20 | 1 | 2 | 0 |
| M2 | ML- | D1 | P20 | 2 | 2 | 0 |
| M2 | ML- | D1 | P20 | 3 | 2 | 0 |
| M2 | ML- | D1 | P20 | 4 | 2 | 0 |
| M2 | ML- | D1 | P21 | 1 | 2 | 0 |
| M2 | ML- | D1 | P21 | 2 | 2 | 0 |
| M2 | ML- | D1 | P21 | 3 | 2 | 0 |
| M2 | ML- | D1 | P21 | 4 | 2 | 0 |
| M2 | ML- | D1 | P22 | 1 | 2 | 0 |
| M2 | ML- | D1 | P22 | 2 | 2 | 0 |
| M2 | ML- | D1 | P22 | 3 | 2 | 0 |
| M2 | ML- | D1 | P22 | 4 | 2 | 0 |
| M2 | ML- | D1 | P23 | 1 | 2 | 0 |
| M2 | ML- | D1 | P23 | 2 | 2 | 0 |
| M2 | ML- | D1 | P23 | 3 | 2 | 0 |
| M2 | ML- | D1 | P23 | 4 | 2 | 0 |
| M2 | ML- | D1 | P24 | 1 | 2 | 0 |
| M2 | ML- | D1 | P24 | 2 | 2 | 0 |
| M2 | ML- | D1 | P24 | 3 | 2 | 0 |
| M2 | ML- | D1 | P24 | 4 | 2 | 0 |
| M2 | ML+ | D1 | P1 | 1 | 2 | 0 |
| M2 | ML+ | D1 | P1 | 2 | 2 | 0 |

| | | | | | | |
|---|---|---|---|---|---|---|
| M2 | ML+ | D1 | P1 | 3 | 2 | 0 |
| M2 | ML+ | D1 | P1 | 4 | 2 | 0 |
| M2 | ML+ | D1 | P2 | 1 | 2 | 0 |
| M2 | ML+ | D1 | P2 | 2 | 2 | 0 |
| M2 | ML+ | D1 | P2 | 3 | 2 | 0 |
| M2 | ML+ | D1 | P2 | 4 | 2 | 0 |
| M2 | ML+ | D1 | P3 | 1 | 2 | 0 |
| M2 | ML+ | D1 | P3 | 2 | 2 | 0 |
| M2 | ML+ | D1 | P3 | 3 | 2 | 0 |
| M2 | ML+ | D1 | P3 | 4 | 2 | 0 |
| M2 | ML+ | D1 | P4 | 1 | 2 | 0 |
| M2 | ML+ | D1 | P4 | 2 | 2 | 0 |
| M2 | ML+ | D1 | P4 | 3 | 2 | 0 |
| M2 | ML+ | D1 | P4 | 4 | 2 | 0 |
| M2 | ML+ | D1 | P5 | 1 | 2 | 0 |
| M2 | ML+ | D1 | P5 | 2 | 2 | 0 |
| M2 | ML+ | D1 | P5 | 3 | 2 | 0 |
| M2 | ML+ | D1 | P5 | 4 | 2 | 0 |
| M2 | ML+ | D1 | P6 | 1 | 2 | 0 |
| M2 | ML+ | D1 | P6 | 2 | 2 | 0 |
| M2 | ML+ | D1 | P6 | 3 | 2 | 0 |
| M2 | ML+ | D1 | P6 | 4 | 2 | 0 |
| M2 | ML+ | D1 | P7 | 1 | 2 | 0 |
| M2 | ML+ | D1 | P7 | 2 | 2 | 0 |
| M2 | ML+ | D1 | P7 | 3 | 2 | 0 |
| M2 | ML+ | D1 | P7 | 4 | 2 | 0 |
| M2 | ML+ | D1 | P8 | 1 | 2 | 0 |
| M2 | ML+ | D1 | P8 | 2 | 2 | 0 |
| M2 | ML+ | D1 | P8 | 3 | 2 | 0 |
| M2 | ML+ | D1 | P8 | 4 | 2 | 0 |
| M2 | ML+ | D1 | P9 | 1 | 2 | 0 |
| M2 | ML+ | D1 | P9 | 2 | 2 | 0 |
| M2 | ML+ | D1 | P9 | 3 | 2 | 0 |
| M2 | ML+ | D1 | P9 | 4 | 2 | 0 |
| M2 | ML+ | D1 | P10 | 1 | 2 | 0 |
| M2 | ML+ | D1 | P10 | 2 | 2 | 0 |
| M2 | ML+ | D1 | P10 | 3 | 2 | 0 |
| M2 | ML+ | D1 | P10 | 4 | 2 | 0 |
| M2 | ML+ | D1 | P11 | 1 | 2 | 0 |
| M2 | ML+ | D1 | P11 | 2 | 2 | 0 |
| M2 | ML+ | D1 | P11 | 3 | 2 | 0 |
| M2 | ML+ | D1 | P11 | 4 | 2 | 0 |
| M2 | ML+ | D1 | P12 | 1 | 2 | 0 |
| M2 | ML+ | D1 | P12 | 2 | 2 | 0 |
| M2 | ML+ | D1 | P12 | 3 | 2 | 0 |
| M2 | ML+ | D1 | P12 | 4 | 2 | 0 |
| M2 | ML+ | D1 | P13 | 1 | 2 | 0 |
| M2 | ML+ | D1 | P13 | 2 | 2 | 0 |
| M2 | ML+ | D1 | P13 | 3 | 2 | 0 |
| M2 | ML+ | D1 | P13 | 4 | 2 | 0 |

| | | | | | | |
|---|---|---|---|---|---|---|
| M2 | ML+ | D1 | P14 | 1 | 2 | 0 |
| M2 | ML+ | D1 | P14 | 2 | 2 | 0 |
| M2 | ML+ | D1 | P14 | 3 | 2 | 0 |
| M2 | ML+ | D1 | P14 | 4 | 2 | 0 |
| M2 | ML+ | D1 | P15 | 1 | 2 | 0 |
| M2 | ML+ | D1 | P15 | 2 | 2 | 0 |
| M2 | ML+ | D1 | P15 | 3 | 2 | 0 |
| M2 | ML+ | D1 | P15 | 4 | 2 | 0 |
| M2 | ML+ | D1 | P16 | 1 | 2 | 0 |
| M2 | ML+ | D1 | P16 | 2 | 2 | 0 |
| M2 | ML+ | D1 | P16 | 3 | 2 | 0 |
| M2 | ML+ | D1 | P16 | 4 | 2 | 0 |
| M2 | ML+ | D1 | P17 | 1 | 2 | 0 |
| M2 | ML+ | D1 | P17 | 2 | 2 | 0 |
| M2 | ML+ | D1 | P17 | 3 | 2 | 0 |
| M2 | ML+ | D1 | P17 | 4 | 2 | 0 |
| M2 | ML+ | D1 | P18 | 1 | 2 | 0 |
| M2 | ML+ | D1 | P18 | 2 | 2 | 0 |
| M2 | ML+ | D1 | P18 | 3 | 2 | 0 |
| M2 | ML+ | D1 | P18 | 4 | 2 | 0 |
| M2 | ML+ | D1 | P19 | 1 | 2 | 0 |
| M2 | ML+ | D1 | P19 | 2 | 2 | 0 |
| M2 | ML+ | D1 | P19 | 3 | 2 | 0 |
| M2 | ML+ | D1 | P19 | 4 | 2 | 0 |
| M2 | ML+ | D1 | P20 | 1 | 2 | 0 |
| M2 | ML+ | D1 | P20 | 2 | 2 | 0 |
| M2 | ML+ | D1 | P20 | 3 | 2 | 0 |
| M2 | ML+ | D1 | P20 | 4 | 2 | 0 |
| M2 | ML+ | D1 | P21 | 1 | 2 | 0 |
| M2 | ML+ | D1 | P21 | 2 | 2 | 0 |
| M2 | ML+ | D1 | P21 | 3 | 2 | 0 |
| M2 | ML+ | D1 | P21 | 4 | 2 | 0 |
| M2 | ML+ | D1 | P22 | 1 | 2 | 0 |
| M2 | ML+ | D1 | P22 | 2 | 2 | 0 |
| M2 | ML+ | D1 | P22 | 3 | 2 | 0 |
| M2 | ML+ | D1 | P22 | 4 | 2 | 0 |
| M2 | ML+ | D1 | P23 | 1 | 2 | 0 |
| M2 | ML+ | D1 | P23 | 2 | 2 | 0 |
| M2 | ML+ | D1 | P23 | 3 | 2 | 0 |
| M2 | ML+ | D1 | P23 | 4 | 2 | 0 |
| M2 | ML+ | D1 | P24 | 1 | 2 | 0 |
| M2 | ML+ | D1 | P24 | 2 | 2 | 0 |
| M2 | ML+ | D1 | P24 | 3 | 2 | 0 |
| M2 | ML+ | D1 | P24 | 4 | 2 | 0 |
| M2 | ML- | D1 | P1 | 1 | 9 | 0 |
| M2 | ML- | D1 | P1 | 2 | 9 | 1 |
| M2 | ML- | D1 | P1 | 3 | 9 | 1 |
| M2 | ML- | D1 | P1 | 4 | 9 | 0 |
| M2 | ML- | D1 | P2 | 1 | 9 | 0 |
| M2 | ML- | D1 | P2 | 2 | 9 | 0 |

| | | | | | | |
|---|---|---|---|---|---|---|
| M2 | ML- | D1 | P2 | 3 | 9 | 1 |
| M2 | ML- | D1 | P2 | 4 | 9 | 0 |
| M2 | ML- | D1 | P3 | 1 | 9 | 1 |
| M2 | ML- | D1 | P3 | 2 | 9 | 1 |
| M2 | ML- | D1 | P3 | 3 | 9 | 0 |
| M2 | ML- | D1 | P3 | 4 | 9 | 1 |
| M2 | ML- | D1 | P4 | 1 | 9 | 1 |
| M2 | ML- | D1 | P4 | 2 | 9 | 1 |
| M2 | ML- | D1 | P4 | 3 | 9 | 0 |
| M2 | ML- | D1 | P4 | 4 | 9 | 0 |
| M2 | ML- | D1 | P5 | 1 | 9 | 0 |
| M2 | ML- | D1 | P5 | 2 | 9 | 0 |
| M2 | ML- | D1 | P5 | 3 | 9 | 0 |
| M2 | ML- | D1 | P5 | 4 | 9 | 1 |
| M2 | ML- | D1 | P6 | 1 | 9 | 0 |
| M2 | ML- | D1 | P6 | 2 | 9 | 0 |
| M2 | ML- | D1 | P6 | 3 | 9 | 0 |
| M2 | ML- | D1 | P6 | 4 | 9 | 1 |
| M2 | ML- | D1 | P7 | 1 | 9 | 0 |
| M2 | ML- | D1 | P7 | 2 | 9 | 1 |
| M2 | ML- | D1 | P7 | 3 | 9 | 0 |
| M2 | ML- | D1 | P7 | 4 | 9 | 1 |
| M2 | ML- | D1 | P8 | 1 | 9 | 1 |
| M2 | ML- | D1 | P8 | 2 | 9 | 1 |
| M2 | ML- | D1 | P8 | 3 | 9 | 1 |
| M2 | ML- | D1 | P8 | 4 | 9 | 1 |
| M2 | ML- | D1 | P9 | 1 | 9 | 0 |
| M2 | ML- | D1 | P9 | 2 | 9 | 0 |
| M2 | ML- | D1 | P9 | 3 | 9 | 1 |
| M2 | ML- | D1 | P9 | 4 | 9 | 1 |
| M2 | ML- | D1 | P10 | 1 | 9 | 1 |
| M2 | ML- | D1 | P10 | 2 | 9 | 1 |
| M2 | ML- | D1 | P10 | 3 | 9 | 1 |
| M2 | ML- | D1 | P10 | 4 | 9 | 1 |
| M2 | ML- | D1 | P11 | 1 | 9 | 0 |
| M2 | ML- | D1 | P11 | 2 | 9 | 1 |
| M2 | ML- | D1 | P11 | 3 | 9 | 1 |
| M2 | ML- | D1 | P11 | 4 | 9 | 0 |
| M2 | ML- | D1 | P12 | 1 | 9 | 0 |
| M2 | ML- | D1 | P12 | 2 | 9 | 0 |
| M2 | ML- | D1 | P12 | 3 | 9 | 0 |
| M2 | ML- | D1 | P12 | 4 | 9 | 0 |
| M2 | ML- | D1 | P13 | 1 | 9 | 0 |
| M2 | ML- | D1 | P13 | 2 | 9 | 1 |
| M2 | ML- | D1 | P13 | 3 | 9 | 1 |
| M2 | ML- | D1 | P13 | 4 | 9 | 1 |
| M2 | ML- | D1 | P14 | 1 | 9 | 0 |
| M2 | ML- | D1 | P14 | 2 | 9 | 0 |
| M2 | ML- | D1 | P14 | 3 | 9 | 0 |
| M2 | ML- | D1 | P14 | 4 | 9 | 0 |

| | | | | | | |
|---|---|---|---|---|---|---|
| M2 | ML- | D1 | P15 | 1 | 9 | 1 |
| M2 | ML- | D1 | P15 | 2 | 9 | 0 |
| M2 | ML- | D1 | P15 | 3 | 9 | 0 |
| M2 | ML- | D1 | P15 | 4 | 9 | 1 |
| M2 | ML- | D1 | P16 | 1 | 9 | 1 |
| M2 | ML- | D1 | P16 | 2 | 9 | 0 |
| M2 | ML- | D1 | P16 | 3 | 9 | 0 |
| M2 | ML- | D1 | P16 | 4 | 9 | 0 |
| M2 | ML- | D1 | P17 | 1 | 9 | 0 |
| M2 | ML- | D1 | P17 | 2 | 9 | 0 |
| M2 | ML- | D1 | P17 | 3 | 9 | 1 |
| M2 | ML- | D1 | P17 | 4 | 9 | 1 |
| M2 | ML- | D1 | P18 | 1 | 9 | 1 |
| M2 | ML- | D1 | P18 | 2 | 9 | 1 |
| M2 | ML- | D1 | P18 | 3 | 9 | 1 |
| M2 | ML- | D1 | P18 | 4 | 9 | 0 |
| M2 | ML- | D1 | P19 | 1 | 9 | 0 |
| M2 | ML- | D1 | P19 | 2 | 9 | 0 |
| M2 | ML- | D1 | P19 | 3 | 9 | 1 |
| M2 | ML- | D1 | P19 | 4 | 9 | 0 |
| M2 | ML- | D1 | P20 | 1 | 9 | 0 |
| M2 | ML- | D1 | P20 | 2 | 9 | 1 |
| M2 | ML- | D1 | P20 | 3 | 9 | 1 |
| M2 | ML- | D1 | P20 | 4 | 9 | 1 |
| M2 | ML- | D1 | P21 | 1 | 9 | 1 |
| M2 | ML- | D1 | P21 | 2 | 9 | 1 |
| M2 | ML- | D1 | P21 | 3 | 9 | 1 |
| M2 | ML- | D1 | P21 | 4 | 9 | 0 |
| M2 | ML- | D1 | P22 | 1 | 9 | 0 |
| M2 | ML- | D1 | P22 | 2 | 9 | 0 |
| M2 | ML- | D1 | P22 | 3 | 9 | 1 |
| M2 | ML- | D1 | P22 | 4 | 9 | 1 |
| M2 | ML- | D1 | P23 | 1 | 9 | 0 |
| M2 | ML- | D1 | P23 | 2 | 9 | 1 |
| M2 | ML- | D1 | P23 | 3 | 9 | 1 |
| M2 | ML- | D1 | P23 | 4 | 9 | 0 |
| M2 | ML- | D1 | P24 | 1 | 9 | 0 |
| M2 | ML- | D1 | P24 | 2 | 9 | 1 |
| M2 | ML- | D1 | P24 | 3 | 9 | 1 |
| M2 | ML- | D1 | P24 | 4 | 9 | 0 |
| M2 | ML+ | D1 | P1 | 1 | 9 | 0 |
| M2 | ML+ | D1 | P1 | 2 | 9 | 0 |
| M2 | ML+ | D1 | P1 | 3 | 9 | 0 |
| M2 | ML+ | D1 | P1 | 4 | 9 | 0 |
| M2 | ML+ | D1 | P2 | 1 | 9 | 0 |
| M2 | ML+ | D1 | P2 | 2 | 9 | 0 |
| M2 | ML+ | D1 | P2 | 3 | 9 | 0 |
| M2 | ML+ | D1 | P2 | 4 | 9 | 0 |
| M2 | ML+ | D1 | P3 | 1 | 9 | 0 |
| M2 | ML+ | D1 | P3 | 2 | 9 | 0 |

| | | | | | | |
|---|---|---|---|---|---|---|
| M2 | ML+ | D1 | P3 | 3 | 9 | 0 |
| M2 | ML+ | D1 | P3 | 4 | 9 | 0 |
| M2 | ML+ | D1 | P4 | 1 | 9 | 0 |
| M2 | ML+ | D1 | P4 | 2 | 9 | 0 |
| M2 | ML+ | D1 | P4 | 3 | 9 | 0 |
| M2 | ML+ | D1 | P4 | 4 | 9 | 0 |
| M2 | ML+ | D1 | P5 | 1 | 9 | 0 |
| M2 | ML+ | D1 | P5 | 2 | 9 | 0 |
| M2 | ML+ | D1 | P5 | 3 | 9 | 0 |
| M2 | ML+ | D1 | P5 | 4 | 9 | 0 |
| M2 | ML+ | D1 | P6 | 1 | 9 | 0 |
| M2 | ML+ | D1 | P6 | 2 | 9 | 0 |
| M2 | ML+ | D1 | P6 | 3 | 9 | 0 |
| M2 | ML+ | D1 | P6 | 4 | 9 | 0 |
| M2 | ML+ | D1 | P7 | 1 | 9 | 0 |
| M2 | ML+ | D1 | P7 | 2 | 9 | 0 |
| M2 | ML+ | D1 | P7 | 3 | 9 | 0 |
| M2 | ML+ | D1 | P7 | 4 | 9 | 0 |
| M2 | ML+ | D1 | P8 | 1 | 9 | 0 |
| M2 | ML+ | D1 | P8 | 2 | 9 | 0 |
| M2 | ML+ | D1 | P8 | 3 | 9 | 0 |
| M2 | ML+ | D1 | P8 | 4 | 9 | 0 |
| M2 | ML+ | D1 | P9 | 1 | 9 | 0 |
| M2 | ML+ | D1 | P9 | 2 | 9 | 0 |
| M2 | ML+ | D1 | P9 | 3 | 9 | 0 |
| M2 | ML+ | D1 | P9 | 4 | 9 | 0 |
| M2 | ML+ | D1 | P10 | 1 | 9 | 0 |
| M2 | ML+ | D1 | P10 | 2 | 9 | 0 |
| M2 | ML+ | D1 | P10 | 3 | 9 | 0 |
| M2 | ML+ | D1 | P10 | 4 | 9 | 0 |
| M2 | ML+ | D1 | P11 | 1 | 9 | 0 |
| M2 | ML+ | D1 | P11 | 2 | 9 | 0 |
| M2 | ML+ | D1 | P11 | 3 | 9 | 0 |
| M2 | ML+ | D1 | P11 | 4 | 9 | 0 |
| M2 | ML+ | D1 | P12 | 1 | 9 | 0 |
| M2 | ML+ | D1 | P12 | 2 | 9 | 0 |
| M2 | ML+ | D1 | P12 | 3 | 9 | 0 |
| M2 | ML+ | D1 | P12 | 4 | 9 | 0 |
| M2 | ML+ | D1 | P13 | 1 | 9 | 0 |
| M2 | ML+ | D1 | P13 | 2 | 9 | 0 |
| M2 | ML+ | D1 | P13 | 3 | 9 | 0 |
| M2 | ML+ | D1 | P13 | 4 | 9 | 0 |
| M2 | ML+ | D1 | P14 | 1 | 9 | 0 |
| M2 | ML+ | D1 | P14 | 2 | 9 | 0 |
| M2 | ML+ | D1 | P14 | 3 | 9 | 0 |
| M2 | ML+ | D1 | P14 | 4 | 9 | 0 |
| M2 | ML+ | D1 | P15 | 1 | 9 | 0 |
| M2 | ML+ | D1 | P15 | 2 | 9 | 0 |
| M2 | ML+ | D1 | P15 | 3 | 9 | 0 |
| M2 | ML+ | D1 | P15 | 4 | 9 | 0 |

| | | | | | | |
|---|---|---|---|---|---|---|
| M2 | ML+ | D1 | P16 | 1 | 9 | 0 |
| M2 | ML+ | D1 | P16 | 2 | 9 | 0 |
| M2 | ML+ | D1 | P16 | 3 | 9 | 0 |
| M2 | ML+ | D1 | P16 | 4 | 9 | 0 |
| M2 | ML+ | D1 | P17 | 1 | 9 | 0 |
| M2 | ML+ | D1 | P17 | 2 | 9 | 0 |
| M2 | ML+ | D1 | P17 | 3 | 9 | 0 |
| M2 | ML+ | D1 | P17 | 4 | 9 | 0 |
| M2 | ML+ | D1 | P18 | 1 | 9 | 0 |
| M2 | ML+ | D1 | P18 | 2 | 9 | 0 |
| M2 | ML+ | D1 | P18 | 3 | 9 | 0 |
| M2 | ML+ | D1 | P18 | 4 | 9 | 0 |
| M2 | ML+ | D1 | P19 | 1 | 9 | 0 |
| M2 | ML+ | D1 | P19 | 2 | 9 | 0 |
| M2 | ML+ | D1 | P19 | 3 | 9 | 0 |
| M2 | ML+ | D1 | P19 | 4 | 9 | 0 |
| M2 | ML+ | D1 | P20 | 1 | 9 | 0 |
| M2 | ML+ | D1 | P20 | 2 | 9 | 0 |
| M2 | ML+ | D1 | P20 | 3 | 9 | 0 |
| M2 | ML+ | D1 | P20 | 4 | 9 | 0 |
| M2 | ML+ | D1 | P21 | 1 | 9 | 0 |
| M2 | ML+ | D1 | P21 | 2 | 9 | 0 |
| M2 | ML+ | D1 | P21 | 3 | 9 | 0 |
| M2 | ML+ | D1 | P21 | 4 | 9 | 0 |
| M2 | ML+ | D1 | P22 | 1 | 9 | 0 |
| M2 | ML+ | D1 | P22 | 2 | 9 | 0 |
| M2 | ML+ | D1 | P22 | 3 | 9 | 0 |
| M2 | ML+ | D1 | P22 | 4 | 9 | 0 |
| M2 | ML+ | D1 | P23 | 1 | 9 | 0 |
| M2 | ML+ | D1 | P23 | 2 | 9 | 0 |
| M2 | ML+ | D1 | P23 | 3 | 9 | 0 |
| M2 | ML+ | D1 | P23 | 4 | 9 | 0 |
| M2 | ML+ | D1 | P24 | 1 | 9 | 0 |
| M2 | ML+ | D1 | P24 | 2 | 9 | 0 |
| M2 | ML+ | D1 | P24 | 3 | 9 | 0 |
| M2 | ML+ | D1 | P24 | 4 | 9 | 0 |
| M2 | ML- | D1 | P1 | 1 | 16 | 0 |
| M2 | ML- | D1 | P1 | 2 | 16 | 1 |
| M2 | ML- | D1 | P1 | 3 | 16 | 1 |
| M2 | ML- | D1 | P1 | 4 | 16 | 0 |
| M2 | ML- | D1 | P2 | 1 | 16 | 0 |
| M2 | ML- | D1 | P2 | 2 | 16 | 0 |
| M2 | ML- | D1 | P2 | 3 | 16 | 1 |
| M2 | ML- | D1 | P2 | 4 | 16 | 0 |
| M2 | ML- | D1 | P3 | 1 | 16 | 1 |
| M2 | ML- | D1 | P3 | 2 | 16 | 1 |
| M2 | ML- | D1 | P3 | 3 | 16 | 1 |
| M2 | ML- | D1 | P3 | 4 | 16 | 1 |
| M2 | ML- | D1 | P4 | 1 | 16 | 1 |
| M2 | ML- | D1 | P4 | 2 | 16 | 1 |

| | | | | | | |
|---|---|---|---|---|---|---|
| M2 | ML- | D1 | P4 | 3 | 16 | 1 |
| M2 | ML- | D1 | P4 | 4 | 16 | 0 |
| M2 | ML- | D1 | P5 | 1 | 16 | 1 |
| M2 | ML- | D1 | P5 | 2 | 16 | 0 |
| M2 | ML- | D1 | P5 | 3 | 16 | 0 |
| M2 | ML- | D1 | P5 | 4 | 16 | 1 |
| M2 | ML- | D1 | P6 | 1 | 16 | 0 |
| M2 | ML- | D1 | P6 | 2 | 16 | 0 |
| M2 | ML- | D1 | P6 | 3 | 16 | 1 |
| M2 | ML- | D1 | P6 | 4 | 16 | 1 |
| M2 | ML- | D1 | P7 | 1 | 16 | 0 |
| M2 | ML- | D1 | P7 | 2 | 16 | 1 |
| M2 | ML- | D1 | P7 | 3 | 16 | 0 |
| M2 | ML- | D1 | P7 | 4 | 16 | 1 |
| M2 | ML- | D1 | P8 | 1 | 16 | 1 |
| M2 | ML- | D1 | P8 | 2 | 16 | 1 |
| M2 | ML- | D1 | P8 | 3 | 16 | 0 |
| M2 | ML- | D1 | P8 | 4 | 16 | 1 |
| M2 | ML- | D1 | P9 | 1 | 16 | 1 |
| M2 | ML- | D1 | P9 | 2 | 16 | 0 |
| M2 | ML- | D1 | P9 | 3 | 16 | 1 |
| M2 | ML- | D1 | P9 | 4 | 16 | 1 |
| M2 | ML- | D1 | P10 | 1 | 16 | 1 |
| M2 | ML- | D1 | P10 | 2 | 16 | 1 |
| M2 | ML- | D1 | P10 | 3 | 16 | 1 |
| M2 | ML- | D1 | P10 | 4 | 16 | 1 |
| M2 | ML- | D1 | P11 | 1 | 16 | 0 |
| M2 | ML- | D1 | P11 | 2 | 16 | 1 |
| M2 | ML- | D1 | P11 | 3 | 16 | 0 |
| M2 | ML- | D1 | P11 | 4 | 16 | 0 |
| M2 | ML- | D1 | P12 | 1 | 16 | 1 |
| M2 | ML- | D1 | P12 | 2 | 16 | 0 |
| M2 | ML- | D1 | P12 | 3 | 16 | 0 |
| M2 | ML- | D1 | P12 | 4 | 16 | 0 |
| M2 | ML- | D1 | P13 | 1 | 16 | 0 |
| M2 | ML- | D1 | P13 | 2 | 16 | 1 |
| M2 | ML- | D1 | P13 | 3 | 16 | 1 |
| M2 | ML- | D1 | P13 | 4 | 16 | 1 |
| M2 | ML- | D1 | P14 | 1 | 16 | 0 |
| M2 | ML- | D1 | P14 | 2 | 16 | 0 |
| M2 | ML- | D1 | P14 | 3 | 16 | 1 |
| M2 | ML- | D1 | P14 | 4 | 16 | 0 |
| M2 | ML- | D1 | P15 | 1 | 16 | 1 |
| M2 | ML- | D1 | P15 | 2 | 16 | 1 |
| M2 | ML- | D1 | P15 | 3 | 16 | 1 |
| M2 | ML- | D1 | P15 | 4 | 16 | 1 |
| M2 | ML- | D1 | P16 | 1 | 16 na | |
| M2 | ML- | D1 | P16 | 2 | 16 | 0 |
| M2 | ML- | D1 | P16 | 3 | 16 | 0 |
| M2 | ML- | D1 | P16 | 4 | 16 | 0 |

| | | | | | | |
|---|---|---|---|---|---|---|
| M2 | ML- | D1 | P17 | 1 | 16 | 1 |
| M2 | ML- | D1 | P17 | 2 | 16 | 0 |
| M2 | ML- | D1 | P17 | 3 | 16 | 1 |
| M2 | ML- | D1 | P17 | 4 | 16 | 1 |
| M2 | ML- | D1 | P18 | 1 | 16 | 1 |
| M2 | ML- | D1 | P18 | 2 | 16 | 1 |
| M2 | ML- | D1 | P18 | 3 | 16 | 1 |
| M2 | ML- | D1 | P18 | 4 | 16 | 0 |
| M2 | ML- | D1 | P19 | 1 | 16 | 1 |
| M2 | ML- | D1 | P19 | 2 | 16 | 1 |
| M2 | ML- | D1 | P19 | 3 | 16 | 1 |
| M2 | ML- | D1 | P19 | 4 | 16 | 1 |
| M2 | ML- | D1 | P20 | 1 | 16 | 1 |
| M2 | ML- | D1 | P20 | 2 | 16 | 1 |
| M2 | ML- | D1 | P20 | 3 | 16 | 1 |
| M2 | ML- | D1 | P20 | 4 | 16 | 1 |
| M2 | ML- | D1 | P21 | 1 | 16 | 1 |
| M2 | ML- | D1 | P21 | 2 | 16 | 1 |
| M2 | ML- | D1 | P21 | 3 | 16 | 1 |
| M2 | ML- | D1 | P21 | 4 | 16 | 1 |
| M2 | ML- | D1 | P22 | 1 | 16 | 0 |
| M2 | ML- | D1 | P22 | 2 | 16 | 1 |
| M2 | ML- | D1 | P22 | 3 | 16 | 1 |
| M2 | ML- | D1 | P22 | 4 | 16 | 1 |
| M2 | ML- | D1 | P23 | 1 | 16 | 0 |
| M2 | ML- | D1 | P23 | 2 | 16 | 1 |
| M2 | ML- | D1 | P23 | 3 | 16 | 1 |
| M2 | ML- | D1 | P23 | 4 | 16 | 0 |
| M2 | ML- | D1 | P24 | 1 | 16 | 1 |
| M2 | ML- | D1 | P24 | 2 | 16 | 1 |
| M2 | ML- | D1 | P24 | 3 | 16 | 1 |
| M2 | ML- | D1 | P24 | 4 | 16 | 0 |
| M2 | ML+ | D1 | P1 | 1 | 16 | 0 |
| M2 | ML+ | D1 | P1 | 2 | 16 | 0 |
| M2 | ML+ | D1 | P1 | 3 | 16 | 0 |
| M2 | ML+ | D1 | P1 | 4 | 16 | 0 |
| M2 | ML+ | D1 | P2 | 1 | 16 | 0 |
| M2 | ML+ | D1 | P2 | 2 | 16 | 0 |
| M2 | ML+ | D1 | P2 | 3 | 16 | 0 |
| M2 | ML+ | D1 | P2 | 4 | 16 | 0 |
| M2 | ML+ | D1 | P3 | 1 | 16 | 0 |
| M2 | ML+ | D1 | P3 | 2 | 16 | 0 |
| M2 | ML+ | D1 | P3 | 3 | 16 | 0 |
| M2 | ML+ | D1 | P3 | 4 | 16 | 0 |
| M2 | ML+ | D1 | P4 | 1 | 16 | 0 |
| M2 | ML+ | D1 | P4 | 2 | 16 | 0 |
| M2 | ML+ | D1 | P4 | 3 | 16 | 0 |
| M2 | ML+ | D1 | P4 | 4 | 16 | 0 |
| M2 | ML+ | D1 | P5 | 1 | 16 | 0 |
| M2 | ML+ | D1 | P5 | 2 | 16 | 0 |

| | | | | | | |
|---|---|---|---|---|---|---|
| M2 | ML+ | D1 | P5 | 3 | 16 | 0 |
| M2 | ML+ | D1 | P5 | 4 | 16 | 0 |
| M2 | ML+ | D1 | P6 | 1 | 16 | 0 |
| M2 | ML+ | D1 | P6 | 2 | 16 | 0 |
| M2 | ML+ | D1 | P6 | 3 | 16 | 0 |
| M2 | ML+ | D1 | P6 | 4 | 16 | 0 |
| M2 | ML+ | D1 | P7 | 1 | 16 | 0 |
| M2 | ML+ | D1 | P7 | 2 | 16 | 0 |
| M2 | ML+ | D1 | P7 | 3 | 16 | 0 |
| M2 | ML+ | D1 | P7 | 4 | 16 | 0 |
| M2 | ML+ | D1 | P8 | 1 | 16 | 0 |
| M2 | ML+ | D1 | P8 | 2 | 16 | 0 |
| M2 | ML+ | D1 | P8 | 3 | 16 | 0 |
| M2 | ML+ | D1 | P8 | 4 | 16 | 0 |
| M2 | ML+ | D1 | P9 | 1 | 16 | 0 |
| M2 | ML+ | D1 | P9 | 2 | 16 | 0 |
| M2 | ML+ | D1 | P9 | 3 | 16 | 0 |
| M2 | ML+ | D1 | P9 | 4 | 16 | 0 |
| M2 | ML+ | D1 | P10 | 1 | 16 | 0 |
| M2 | ML+ | D1 | P10 | 2 | 16 | 0 |
| M2 | ML+ | D1 | P10 | 3 | 16 | 0 |
| M2 | ML+ | D1 | P10 | 4 | 16 | 0 |
| M2 | ML+ | D1 | P11 | 1 | 16 | 0 |
| M2 | ML+ | D1 | P11 | 2 | 16 | 0 |
| M2 | ML+ | D1 | P11 | 3 | 16 | 0 |
| M2 | ML+ | D1 | P11 | 4 | 16 | 0 |
| M2 | ML+ | D1 | P12 | 1 | 16 | 0 |
| M2 | ML+ | D1 | P12 | 2 | 16 | 0 |
| M2 | ML+ | D1 | P12 | 3 | 16 | 0 |
| M2 | ML+ | D1 | P12 | 4 | 16 | 0 |
| M2 | ML+ | D1 | P13 | 1 | 16 | 0 |
| M2 | ML+ | D1 | P13 | 2 | 16 | 0 |
| M2 | ML+ | D1 | P13 | 3 | 16 | 0 |
| M2 | ML+ | D1 | P13 | 4 | 16 | 0 |
| M2 | ML+ | D1 | P14 | 1 | 16 | 0 |
| M2 | ML+ | D1 | P14 | 2 | 16 | 0 |
| M2 | ML+ | D1 | P14 | 3 | 16 | 0 |
| M2 | ML+ | D1 | P14 | 4 | 16 | 0 |
| M2 | ML+ | D1 | P15 | 1 | 16 | 0 |
| M2 | ML+ | D1 | P15 | 2 | 16 | 0 |
| M2 | ML+ | D1 | P15 | 3 | 16 | 0 |
| M2 | ML+ | D1 | P15 | 4 | 16 | 0 |
| M2 | ML+ | D1 | P16 | 1 | 16 | 0 |
| M2 | ML+ | D1 | P16 | 2 | 16 | 0 |
| M2 | ML+ | D1 | P16 | 3 | 16 | 0 |
| M2 | ML+ | D1 | P16 | 4 | 16 | 0 |
| M2 | ML+ | D1 | P17 | 1 | 16 | 0 |
| M2 | ML+ | D1 | P17 | 2 | 16 | 0 |
| M2 | ML+ | D1 | P17 | 3 | 16 | 0 |
| M2 | ML+ | D1 | P17 | 4 | 16 | 0 |

| | | | | | | |
|---|---|---|---|---|---|---|
| M2 | ML+ | D1 | P18 | 1 | 16 | 0 |
| M2 | ML+ | D1 | P18 | 2 | 16 | 0 |
| M2 | ML+ | D1 | P18 | 3 | 16 | 0 |
| M2 | ML+ | D1 | P18 | 4 | 16 | 0 |
| M2 | ML+ | D1 | P19 | 1 | 16 | 0 |
| M2 | ML+ | D1 | P19 | 2 | 16 | 0 |
| M2 | ML+ | D1 | P19 | 3 | 16 | 0 |
| M2 | ML+ | D1 | P19 | 4 | 16 | 0 |
| M2 | ML+ | D1 | P20 | 1 | 16 | 0 |
| M2 | ML+ | D1 | P20 | 2 | 16 | 0 |
| M2 | ML+ | D1 | P20 | 3 | 16 | 0 |
| M2 | ML+ | D1 | P20 | 4 | 16 | 0 |
| M2 | ML+ | D1 | P21 | 1 | 16 | 0 |
| M2 | ML+ | D1 | P21 | 2 | 16 | 0 |
| M2 | ML+ | D1 | P21 | 3 | 16 | 0 |
| M2 | ML+ | D1 | P21 | 4 | 16 | 0 |
| M2 | ML+ | D1 | P22 | 1 | 16 | 0 |
| M2 | ML+ | D1 | P22 | 2 | 16 | 0 |
| M2 | ML+ | D1 | P22 | 3 | 16 | 0 |
| M2 | ML+ | D1 | P22 | 4 | 16 | 0 |
| M2 | ML+ | D1 | P23 | 1 | 16 | 0 |
| M2 | ML+ | D1 | P23 | 2 | 16 | 0 |
| M2 | ML+ | D1 | P23 | 3 | 16 | 0 |
| M2 | ML+ | D1 | P23 | 4 | 16 | 0 |
| M2 | ML+ | D1 | P24 | 1 | 16 | 0 |
| M2 | ML+ | D1 | P24 | 2 | 16 | 0 |
| M2 | ML+ | D1 | P24 | 3 | 16 | 0 |
| M2 | ML+ | D1 | P24 | 4 | 16 | 0 |
| M1 | ML- | D2 | P1 | 1 | 2 | 0 |
| M1 | ML- | D2 | P1 | 2 | 2 | 0 |
| M1 | ML- | D2 | P1 | 3 | 2 | 0 |
| M1 | ML- | D2 | P1 | 4 | 2 | 0 |
| M1 | ML- | D2 | P2 | 1 | 2 | 0 |
| M1 | ML- | D2 | P2 | 2 | 2 | 0 |
| M1 | ML- | D2 | P2 | 3 | 2 | 0 |
| M1 | ML- | D2 | P2 | 4 | 2 | 0 |
| M1 | ML- | D2 | P3 | 1 | 2 | 0 |
| M1 | ML- | D2 | P3 | 2 | 2 | 0 |
| M1 | ML- | D2 | P3 | 3 | 2 | 0 |
| M1 | ML- | D2 | P3 | 4 | 2 | 0 |
| M1 | ML- | D2 | P4 | 1 | 2 | 0 |
| M1 | ML- | D2 | P4 | 2 | 2 | 0 |
| M1 | ML- | D2 | P4 | 3 | 2 | 0 |
| M1 | ML- | D2 | P4 | 4 | 2 | 0 |
| M1 | ML- | D2 | P5 | 1 | 2 | 0 |
| M1 | ML- | D2 | P5 | 2 | 2 | 0 |
| M1 | ML- | D2 | P5 | 3 | 2 | 0 |
| M1 | ML- | D2 | P5 | 4 | 2 | 0 |
| M1 | ML- | D2 | P6 | 1 | 2 | 0 |
| M1 | ML- | D2 | P6 | 2 | 2 | 0 |

| | | | | | | |
|---|---|---|---|---|---|---|
| M1 | ML- | D2 | P6 | 3 | 2 | 0 |
| M1 | ML- | D2 | P6 | 4 | 2 | 0 |
| M1 | ML- | D2 | P7 | 1 | 2 | 0 |
| M1 | ML- | D2 | P7 | 2 | 2 | 0 |
| M1 | ML- | D2 | P7 | 3 | 2 | 0 |
| M1 | ML- | D2 | P7 | 4 | 2 | 0 |
| M1 | ML- | D2 | P8 | 1 | 2 | 0 |
| M1 | ML- | D2 | P8 | 2 | 2 | 0 |
| M1 | ML- | D2 | P8 | 3 | 2 | 0 |
| M1 | ML- | D2 | P8 | 4 | 2 | 0 |
| M1 | ML- | D2 | P9 | 1 | 2 | 0 |
| M1 | ML- | D2 | P9 | 2 | 2 | 0 |
| M1 | ML- | D2 | P9 | 3 | 2 | 0 |
| M1 | ML- | D2 | P9 | 4 | 2 | 0 |
| M1 | ML- | D2 | P10 | 1 | 2 | 0 |
| M1 | ML- | D2 | P10 | 2 | 2 | 0 |
| M1 | ML- | D2 | P10 | 3 | 2 | 0 |
| M1 | ML- | D2 | P10 | 4 | 2 | 0 |
| M1 | ML- | D2 | P11 | 1 | 2 | 0 |
| M1 | ML- | D2 | P11 | 2 | 2 | 0 |
| M1 | ML- | D2 | P11 | 3 | 2 | 0 |
| M1 | ML- | D2 | P11 | 4 | 2 | 0 |
| M1 | ML- | D2 | P12 | 1 | 2 | 0 |
| M1 | ML- | D2 | P12 | 2 | 2 | 0 |
| M1 | ML- | D2 | P12 | 3 | 2 | 0 |
| M1 | ML- | D2 | P12 | 4 | 2 | 0 |
| M1 | ML- | D2 | P13 | 1 | 2 | 0 |
| M1 | ML- | D2 | P13 | 2 | 2 | 0 |
| M1 | ML- | D2 | P13 | 3 | 2 | 0 |
| M1 | ML- | D2 | P13 | 4 | 2 | 0 |
| M1 | ML- | D2 | P14 | 1 | 2 | 0 |
| M1 | ML- | D2 | P14 | 2 | 2 | 0 |
| M1 | ML- | D2 | P14 | 3 | 2 | 0 |
| M1 | ML- | D2 | P14 | 4 | 2 | 0 |
| M1 | ML+ | D2 | P1 | 1 | 2 | 0 |
| M1 | ML+ | D2 | P1 | 2 | 2 | 0 |
| M1 | ML+ | D2 | P1 | 3 | 2 | 0 |
| M1 | ML+ | D2 | P1 | 4 | 2 | 0 |
| M1 | ML+ | D2 | P2 | 1 | 2 | 0 |
| M1 | ML+ | D2 | P2 | 2 | 2 | 0 |
| M1 | ML+ | D2 | P2 | 3 | 2 | 0 |
| M1 | ML+ | D2 | P2 | 4 | 2 | 0 |
| M1 | ML+ | D2 | P3 | 1 | 2 | 0 |
| M1 | ML+ | D2 | P3 | 2 | 2 | 0 |
| M1 | ML+ | D2 | P3 | 3 | 2 | 0 |
| M1 | ML+ | D2 | P3 | 4 | 2 | 0 |
| M1 | ML+ | D2 | P4 | 1 | 2 | 0 |
| M1 | ML+ | D2 | P4 | 2 | 2 | 0 |
| M1 | ML+ | D2 | P4 | 3 | 2 | 0 |
| M1 | ML+ | D2 | P4 | 4 | 2 | 0 |

| | | | | | | |
|---|---|---|---|---|---|---|
| M1 | ML+ | D2 | P5 | 1 | 2 | 0 |
| M1 | ML+ | D2 | P5 | 2 | 2 | 0 |
| M1 | ML+ | D2 | P5 | 3 | 2 | 0 |
| M1 | ML+ | D2 | P5 | 4 | 2 | 0 |
| M1 | ML+ | D2 | P6 | 1 | 2 | 0 |
| M1 | ML+ | D2 | P6 | 2 | 2 | 0 |
| M1 | ML+ | D2 | P6 | 3 | 2 | 0 |
| M1 | ML+ | D2 | P6 | 4 | 2 | 0 |
| M1 | ML+ | D2 | P7 | 1 | 2 | 0 |
| M1 | ML+ | D2 | P7 | 2 | 2 | 0 |
| M1 | ML+ | D2 | P7 | 3 | 2 | 0 |
| M1 | ML+ | D2 | P7 | 4 | 2 | 0 |
| M1 | ML+ | D2 | P8 | 1 | 2 | 0 |
| M1 | ML+ | D2 | P8 | 2 | 2 | 0 |
| M1 | ML+ | D2 | P8 | 3 | 2 | 0 |
| M1 | ML+ | D2 | P8 | 4 | 2 | 0 |
| M1 | ML+ | D2 | P9 | 1 | 2 | 0 |
| M1 | ML+ | D2 | P9 | 2 | 2 | 0 |
| M1 | ML+ | D2 | P9 | 3 | 2 | 0 |
| M1 | ML+ | D2 | P9 | 4 | 2 | 0 |
| M1 | ML+ | D2 | P10 | 1 | 2 | 0 |
| M1 | ML+ | D2 | P10 | 2 | 2 | 0 |
| M1 | ML+ | D2 | P10 | 3 | 2 | 0 |
| M1 | ML+ | D2 | P10 | 4 | 2 | 0 |
| M1 | ML+ | D2 | P11 | 1 | 2 | 0 |
| M1 | ML+ | D2 | P11 | 2 | 2 | 0 |
| M1 | ML+ | D2 | P11 | 3 | 2 | 0 |
| M1 | ML+ | D2 | P11 | 4 | 2 | 0 |
| M1 | ML+ | D2 | P12 | 1 | 2 | 0 |
| M1 | ML+ | D2 | P12 | 2 | 2 | 0 |
| M1 | ML+ | D2 | P12 | 3 | 2 | 0 |
| M1 | ML+ | D2 | P12 | 4 | 2 | 0 |
| M1 | ML+ | D2 | P13 | 1 | 2 | 0 |
| M1 | ML+ | D2 | P13 | 2 | 2 | 0 |
| M1 | ML+ | D2 | P13 | 3 | 2 | 0 |
| M1 | ML+ | D2 | P13 | 4 | 2 | 0 |
| M1 | ML+ | D2 | P14 | 1 | 2 | 0 |
| M1 | ML+ | D2 | P14 | 2 | 2 | 0 |
| M1 | ML+ | D2 | P14 | 3 | 2 | 0 |
| M1 | ML+ | D2 | P14 | 4 | 2 | 0 |
| M1 | ML- | D2 | P1 | 1 | 9 | 0 |
| M1 | ML- | D2 | P1 | 2 | 9 | 0 |
| M1 | ML- | D2 | P1 | 3 | 9 | 0 |
| M1 | ML- | D2 | P1 | 4 | 9 | 0 |
| M1 | ML- | D2 | P2 | 1 | 9 | 0 |
| M1 | ML- | D2 | P2 | 2 | 9 | 0 |
| M1 | ML- | D2 | P2 | 3 | 9 | 0 |
| M1 | ML- | D2 | P2 | 4 | 9 | 0 |
| M1 | ML- | D2 | P3 | 1 | 9 | 0 |
| M1 | ML- | D2 | P3 | 2 | 9 | 0 |

| | | | | | | |
|---|---|---|---|---|---|---|
| M1 | ML- | D2 | P3 | 3 | 9 | 0 |
| M1 | ML- | D2 | P3 | 4 | 9 | 1 |
| M1 | ML- | D2 | P4 | 1 | 9 | 0 |
| M1 | ML- | D2 | P4 | 2 | 9 | 0 |
| M1 | ML- | D2 | P4 | 3 | 9 | 1 |
| M1 | ML- | D2 | P4 | 4 | 9 | 0 |
| M1 | ML- | D2 | P5 | 1 | 9 | 0 |
| M1 | ML- | D2 | P5 | 2 | 9 | 0 |
| M1 | ML- | D2 | P5 | 3 | 9 | 0 |
| M1 | ML- | D2 | P5 | 4 | 9 | 0 |
| M1 | ML- | D2 | P6 | 1 | 9 | 0 |
| M1 | ML- | D2 | P6 | 2 | 9 | 0 |
| M1 | ML- | D2 | P6 | 3 | 9 | 1 |
| M1 | ML- | D2 | P6 | 4 | 9 | 0 |
| M1 | ML- | D2 | P7 | 1 | 9 | 0 |
| M1 | ML- | D2 | P7 | 2 | 9 | 0 |
| M1 | ML- | D2 | P7 | 3 | 9 | 0 |
| M1 | ML- | D2 | P7 | 4 | 9 | 0 |
| M1 | ML- | D2 | P8 | 1 | 9 | 0 |
| M1 | ML- | D2 | P8 | 2 | 9 | 1 |
| M1 | ML- | D2 | P8 | 3 | 9 | 0 |
| M1 | ML- | D2 | P8 | 4 | 9 | 0 |
| M1 | ML- | D2 | P9 | 1 | 9 | 0 |
| M1 | ML- | D2 | P9 | 2 | 9 | 0 |
| M1 | ML- | D2 | P9 | 3 | 9 | 0 |
| M1 | ML- | D2 | P9 | 4 | 9 | 1 |
| M1 | ML- | D2 | P10 | 1 | 9 | 0 |
| M1 | ML- | D2 | P10 | 2 | 9 | 0 |
| M1 | ML- | D2 | P10 | 3 | 9 | 0 |
| M1 | ML- | D2 | P10 | 4 | 9 | 0 |
| M1 | ML- | D2 | P11 | 1 | 9 | 0 |
| M1 | ML- | D2 | P11 | 2 | 9 | 0 |
| M1 | ML- | D2 | P11 | 3 | 9 | 0 |
| M1 | ML- | D2 | P11 | 4 | 9 | 0 |
| M1 | ML- | D2 | P12 | 1 | 9 | 0 |
| M1 | ML- | D2 | P12 | 2 | 9 | 0 |
| M1 | ML- | D2 | P12 | 3 | 9 | 0 |
| M1 | ML- | D2 | P12 | 4 | 9 | 0 |
| M1 | ML- | D2 | P13 | 1 | 9 | 0 |
| M1 | ML- | D2 | P13 | 2 | 9 | 0 |
| M1 | ML- | D2 | P13 | 3 | 9 | 0 |
| M1 | ML- | D2 | P13 | 4 | 9 | 0 |
| M1 | ML- | D2 | P14 | 1 | 9 | 0 |
| M1 | ML- | D2 | P14 | 2 | 9 | 0 |
| M1 | ML- | D2 | P14 | 3 | 9 | 0 |
| M1 | ML- | D2 | P14 | 4 | 9 | 1 |
| M1 | ML+ | D2 | P1 | 1 | 9 | 0 |
| M1 | ML+ | D2 | P1 | 2 | 9 | 0 |
| M1 | ML+ | D2 | P1 | 3 | 9 | 0 |
| M1 | ML+ | D2 | P1 | 4 | 9 | 0 |

| | | | | | | |
|---|---|---|---|---|---|---|
| M1 | ML+ | D2 | P2 | 1 | 9 | 0 |
| M1 | ML+ | D2 | P2 | 2 | 9 | 0 |
| M1 | ML+ | D2 | P2 | 3 | 9 | 0 |
| M1 | ML+ | D2 | P2 | 4 | 9 | 0 |
| M1 | ML+ | D2 | P3 | 1 | 9 | 0 |
| M1 | ML+ | D2 | P3 | 2 | 9 | 0 |
| M1 | ML+ | D2 | P3 | 3 | 9 | 0 |
| M1 | ML+ | D2 | P3 | 4 | 9 | 0 |
| M1 | ML+ | D2 | P4 | 1 | 9 | 0 |
| M1 | ML+ | D2 | P4 | 2 | 9 | 0 |
| M1 | ML+ | D2 | P4 | 3 | 9 | 0 |
| M1 | ML+ | D2 | P4 | 4 | 9 | 0 |
| M1 | ML+ | D2 | P5 | 1 | 9 | 0 |
| M1 | ML+ | D2 | P5 | 2 | 9 | 0 |
| M1 | ML+ | D2 | P5 | 3 | 9 | 0 |
| M1 | ML+ | D2 | P5 | 4 | 9 | 0 |
| M1 | ML+ | D2 | P6 | 1 | 9 | 0 |
| M1 | ML+ | D2 | P6 | 2 | 9 | 0 |
| M1 | ML+ | D2 | P6 | 3 | 9 | 0 |
| M1 | ML+ | D2 | P6 | 4 | 9 | 0 |
| M1 | ML+ | D2 | P7 | 1 | 9 | 0 |
| M1 | ML+ | D2 | P7 | 2 | 9 | 0 |
| M1 | ML+ | D2 | P7 | 3 | 9 | 0 |
| M1 | ML+ | D2 | P7 | 4 | 9 | 0 |
| M1 | ML+ | D2 | P8 | 1 | 9 | 0 |
| M1 | ML+ | D2 | P8 | 2 | 9 | 0 |
| M1 | ML+ | D2 | P8 | 3 | 9 | 0 |
| M1 | ML+ | D2 | P8 | 4 | 9 | 0 |
| M1 | ML+ | D2 | P9 | 1 | 9 | 0 |
| M1 | ML+ | D2 | P9 | 2 | 9 | 0 |
| M1 | ML+ | D2 | P9 | 3 | 9 | 0 |
| M1 | ML+ | D2 | P9 | 4 | 9 | 0 |
| M1 | ML+ | D2 | P10 | 1 | 9 | 0 |
| M1 | ML+ | D2 | P10 | 2 | 9 | 0 |
| M1 | ML+ | D2 | P10 | 3 | 9 | 0 |
| M1 | ML+ | D2 | P10 | 4 | 9 | 0 |
| M1 | ML+ | D2 | P11 | 1 | 9 | 0 |
| M1 | ML+ | D2 | P11 | 2 | 9 | 0 |
| M1 | ML+ | D2 | P11 | 3 | 9 | 0 |
| M1 | ML+ | D2 | P11 | 4 | 9 | 0 |
| M1 | ML+ | D2 | P12 | 1 | 9 | 0 |
| M1 | ML+ | D2 | P12 | 2 | 9 | 0 |
| M1 | ML+ | D2 | P12 | 3 | 9 | 0 |
| M1 | ML+ | D2 | P12 | 4 | 9 | 0 |
| M1 | ML+ | D2 | P13 | 1 | 9 | 0 |
| M1 | ML+ | D2 | P13 | 2 | 9 | 0 |
| M1 | ML+ | D2 | P13 | 3 | 9 | 0 |
| M1 | ML+ | D2 | P13 | 4 | 9 | 0 |
| M1 | ML+ | D2 | P14 | 1 | 9 | 0 |
| M1 | ML+ | D2 | P14 | 2 | 9 | 0 |

| | | | | | | |
|---|---|---|---|---|---|---|
| M1 | ML+ | D2 | P14 | 3 | 9 | 0 |
| M1 | ML+ | D2 | P14 | 4 | 9 | 0 |
| M1 | ML- | D2 | P1 | 1 | 16 | 0 |
| M1 | ML- | D2 | P1 | 2 | 16 | 0 |
| M1 | ML- | D2 | P1 | 3 | 16 | 0 |
| M1 | ML- | D2 | P1 | 4 | 16 | 0 |
| M1 | ML- | D2 | P2 | 1 | 16 | 0 |
| M1 | ML- | D2 | P2 | 2 | 16 | 0 |
| M1 | ML- | D2 | P2 | 3 | 16 | 0 |
| M1 | ML- | D2 | P2 | 4 | 16 | 0 |
| M1 | ML- | D2 | P3 | 1 | 16 | 0 |
| M1 | ML- | D2 | P3 | 2 | 16 | 0 |
| M1 | ML- | D2 | P3 | 3 | 16 | 0 |
| M1 | ML- | D2 | P3 | 4 | 16 | 1 |
| M1 | ML- | D2 | P4 | 1 | 16 | 0 |
| M1 | ML- | D2 | P4 | 2 | 16 | 0 |
| M1 | ML- | D2 | P4 | 3 | 16 | 1 |
| M1 | ML- | D2 | P4 | 4 | 16 | 0 |
| M1 | ML- | D2 | P5 | 1 | 16 | 0 |
| M1 | ML- | D2 | P5 | 2 | 16 | 0 |
| M1 | ML- | D2 | P5 | 3 | 16 | 0 |
| M1 | ML- | D2 | P5 | 4 | 16 | 0 |
| M1 | ML- | D2 | P6 | 1 | 16 | 0 |
| M1 | ML- | D2 | P6 | 2 | 16 | 0 |
| M1 | ML- | D2 | P6 | 3 | 16 | 0 |
| M1 | ML- | D2 | P6 | 4 | 16 | 0 |
| M1 | ML- | D2 | P7 | 1 | 16 | 0 |
| M1 | ML- | D2 | P7 | 2 | 16 | 0 |
| M1 | ML- | D2 | P7 | 3 | 16 | 0 |
| M1 | ML- | D2 | P7 | 4 | 16 | 0 |
| M1 | ML- | D2 | P8 | 1 | 16 | 0 |
| M1 | ML- | D2 | P8 | 2 | 16 | 1 |
| M1 | ML- | D2 | P8 | 3 | 16 | 0 |
| M1 | ML- | D2 | P8 | 4 | 16 | 0 |
| M1 | ML- | D2 | P9 | 1 | 16 | 0 |
| M1 | ML- | D2 | P9 | 2 | 16 | 0 |
| M1 | ML- | D2 | P9 | 3 | 16 | 0 |
| M1 | ML- | D2 | P9 | 4 | 16 | 1 |
| M1 | ML- | D2 | P10 | 1 | 16 | 0 |
| M1 | ML- | D2 | P10 | 2 | 16 | 0 |
| M1 | ML- | D2 | P10 | 3 | 16 | 0 |
| M1 | ML- | D2 | P10 | 4 | 16 | 0 |
| M1 | ML- | D2 | P11 | 1 | 16 | 0 |
| M1 | ML- | D2 | P11 | 2 | 16 | 0 |
| M1 | ML- | D2 | P11 | 3 | 16 | 0 |
| M1 | ML- | D2 | P11 | 4 | 16 | 0 |
| M1 | ML- | D2 | P12 | 1 | 16 | 0 |
| M1 | ML- | D2 | P12 | 2 | 16 | 0 |
| M1 | ML- | D2 | P12 | 3 | 16 | 0 |
| M1 | ML- | D2 | P12 | 4 | 16 | 0 |

| | | | | | | |
|---|---|---|---|---|---|---|
| M1 | ML- | D2 | P13 | 1 | 16 | 0 |
| M1 | ML- | D2 | P13 | 2 | 16 | 1 |
| M1 | ML- | D2 | P13 | 3 | 16 | 0 |
| M1 | ML- | D2 | P13 | 4 | 16 | 0 |
| M1 | ML- | D2 | P14 | 1 | 16 | 1 |
| M1 | ML- | D2 | P14 | 2 | 16 | 0 |
| M1 | ML- | D2 | P14 | 3 | 16 | 0 |
| M1 | ML- | D2 | P14 | 4 | 16 | 1 |
| M1 | ML+ | D2 | P1 | 1 | 16 | 0 |
| M1 | ML+ | D2 | P1 | 2 | 16 | 0 |
| M1 | ML+ | D2 | P1 | 3 | 16 | 0 |
| M1 | ML+ | D2 | P1 | 4 | 16 | 0 |
| M1 | ML+ | D2 | P2 | 1 | 16 | 0 |
| M1 | ML+ | D2 | P2 | 2 | 16 | 0 |
| M1 | ML+ | D2 | P2 | 3 | 16 | 0 |
| M1 | ML+ | D2 | P2 | 4 | 16 | 0 |
| M1 | ML+ | D2 | P3 | 1 | 16 | 0 |
| M1 | ML+ | D2 | P3 | 2 | 16 | 0 |
| M1 | ML+ | D2 | P3 | 3 | 16 | 0 |
| M1 | ML+ | D2 | P3 | 4 | 16 | 0 |
| M1 | ML+ | D2 | P4 | 1 | 16 | 0 |
| M1 | ML+ | D2 | P4 | 2 | 16 | 0 |
| M1 | ML+ | D2 | P4 | 3 | 16 | 0 |
| M1 | ML+ | D2 | P4 | 4 | 16 | 0 |
| M1 | ML+ | D2 | P5 | 1 | 16 | 0 |
| M1 | ML+ | D2 | P5 | 2 | 16 | 0 |
| M1 | ML+ | D2 | P5 | 3 | 16 | 0 |
| M1 | ML+ | D2 | P5 | 4 | 16 | 0 |
| M1 | ML+ | D2 | P6 | 1 | 16 | 0 |
| M1 | ML+ | D2 | P6 | 2 | 16 | 0 |
| M1 | ML+ | D2 | P6 | 3 | 16 | 0 |
| M1 | ML+ | D2 | P6 | 4 | 16 | 0 |
| M1 | ML+ | D2 | P7 | 1 | 16 | 0 |
| M1 | ML+ | D2 | P7 | 2 | 16 | 0 |
| M1 | ML+ | D2 | P7 | 3 | 16 | 0 |
| M1 | ML+ | D2 | P7 | 4 | 16 | 0 |
| M1 | ML+ | D2 | P8 | 1 | 16 | 0 |
| M1 | ML+ | D2 | P8 | 2 | 16 | 0 |
| M1 | ML+ | D2 | P8 | 3 | 16 | 0 |
| M1 | ML+ | D2 | P8 | 4 | 16 | 0 |
| M1 | ML+ | D2 | P9 | 1 | 16 | 0 |
| M1 | ML+ | D2 | P9 | 2 | 16 | 0 |
| M1 | ML+ | D2 | P9 | 3 | 16 | 0 |
| M1 | ML+ | D2 | P9 | 4 | 16 | 0 |
| M1 | ML+ | D2 | P10 | 1 | 16 | 0 |
| M1 | ML+ | D2 | P10 | 2 | 16 | 0 |
| M1 | ML+ | D2 | P10 | 3 | 16 | 0 |
| M1 | ML+ | D2 | P10 | 4 | 16 | 0 |
| M1 | ML+ | D2 | P11 | 1 | 16 | 0 |
| M1 | ML+ | D2 | P11 | 2 | 16 | 0 |

| | | | | | | |
|---|---|---|---|---|---|---|
| M1 | ML+ | D2 | P11 | 3 | 16 | 0 |
| M1 | ML+ | D2 | P11 | 4 | 16 | 0 |
| M1 | ML+ | D2 | P12 | 1 | 16 | 0 |
| M1 | ML+ | D2 | P12 | 2 | 16 | 0 |
| M1 | ML+ | D2 | P12 | 3 | 16 | 0 |
| M1 | ML+ | D2 | P12 | 4 | 16 | 0 |
| M1 | ML+ | D2 | P13 | 1 | 16 | 0 |
| M1 | ML+ | D2 | P13 | 2 | 16 | 0 |
| M1 | ML+ | D2 | P13 | 3 | 16 | 0 |
| M1 | ML+ | D2 | P13 | 4 | 16 | 0 |
| M1 | ML+ | D2 | P14 | 1 | 16 | 0 |
| M1 | ML+ | D2 | P14 | 2 | 16 | 0 |
| M1 | ML+ | D2 | P14 | 3 | 16 | 0 |
| M1 | ML+ | D2 | P14 | 4 | 16 | 0 |
| M2 | ML- | D2 | P1 | 1 | 2 | 0 |
| M2 | ML- | D2 | P1 | 2 | 2 | 0 |
| M2 | ML- | D2 | P1 | 3 | 2 | 0 |
| M2 | ML- | D2 | P1 | 4 | 2 | 0 |
| M2 | ML- | D2 | P2 | 1 | 2 | 0 |
| M2 | ML- | D2 | P2 | 2 | 2 | 0 |
| M2 | ML- | D2 | P2 | 3 | 2 | 0 |
| M2 | ML- | D2 | P2 | 4 | 2 | 0 |
| M2 | ML- | D2 | P3 | 1 | 2 | 0 |
| M2 | ML- | D2 | P3 | 2 | 2 | 0 |
| M2 | ML- | D2 | P3 | 3 | 2 | 0 |
| M2 | ML- | D2 | P3 | 4 | 2 | 0 |
| M2 | ML- | D2 | P4 | 1 | 2 | 0 |
| M2 | ML- | D2 | P4 | 2 | 2 | 0 |
| M2 | ML- | D2 | P4 | 3 | 2 | 0 |
| M2 | ML- | D2 | P4 | 4 | 2 | 0 |
| M2 | ML- | D2 | P5 | 1 | 2 | 0 |
| M2 | ML- | D2 | P5 | 2 | 2 | 0 |
| M2 | ML- | D2 | P5 | 3 | 2 | 0 |
| M2 | ML- | D2 | P5 | 4 | 2 | 0 |
| M2 | ML- | D2 | P6 | 1 | 2 | 0 |
| M2 | ML- | D2 | P6 | 2 | 2 | 0 |
| M2 | ML- | D2 | P6 | 3 | 2 | 0 |
| M2 | ML- | D2 | P6 | 4 | 2 | 0 |
| M2 | ML- | D2 | P7 | 1 | 2 | 0 |
| M2 | ML- | D2 | P7 | 2 | 2 | 0 |
| M2 | ML- | D2 | P7 | 3 | 2 | 0 |
| M2 | ML- | D2 | P7 | 4 | 2 | 0 |
| M2 | ML- | D2 | P8 | 1 | 2 | 0 |
| M2 | ML- | D2 | P8 | 2 | 2 | 0 |
| M2 | ML- | D2 | P8 | 3 | 2 | 0 |
| M2 | ML- | D2 | P8 | 4 | 2 | 0 |
| M2 | ML- | D2 | P9 | 1 | 2 | 0 |
| M2 | ML- | D2 | P9 | 2 | 2 | 0 |
| M2 | ML- | D2 | P9 | 3 | 2 | 0 |
| M2 | ML- | D2 | P9 | 4 | 2 | 0 |

| | | | | | | |
|---|---|---|---|---|---|---|
| M2 | ML- | D2 | P10 | 1 | 2 | 0 |
| M2 | ML- | D2 | P10 | 2 | 2 | 0 |
| M2 | ML- | D2 | P10 | 3 | 2 | 0 |
| M2 | ML- | D2 | P10 | 4 | 2 | 0 |
| M2 | ML- | D2 | P11 | 1 | 2 | 0 |
| M2 | ML- | D2 | P11 | 2 | 2 | 0 |
| M2 | ML- | D2 | P11 | 3 | 2 | 0 |
| M2 | ML- | D2 | P11 | 4 | 2 | 0 |
| M2 | ML- | D2 | P12 | 1 | 2 | 0 |
| M2 | ML- | D2 | P12 | 2 | 2 | 0 |
| M2 | ML- | D2 | P12 | 3 | 2 | 0 |
| M2 | ML- | D2 | P12 | 4 | 2 | 0 |
| M2 | ML- | D2 | P13 | 1 | 2 | 0 |
| M2 | ML- | D2 | P13 | 2 | 2 | 0 |
| M2 | ML- | D2 | P13 | 3 | 2 | 0 |
| M2 | ML- | D2 | P13 | 4 | 2 | 0 |
| M2 | ML- | D2 | P14 | 1 | 2 | 0 |
| M2 | ML- | D2 | P14 | 2 | 2 | 0 |
| M2 | ML- | D2 | P14 | 3 | 2 | 0 |
| M2 | ML- | D2 | P14 | 4 | 2 | 0 |
| M2 | ML+ | D2 | P1 | 1 | 2 | 0 |
| M2 | ML+ | D2 | P1 | 2 | 2 | 0 |
| M2 | ML+ | D2 | P1 | 3 | 2 | 0 |
| M2 | ML+ | D2 | P1 | 4 | 2 | 0 |
| M2 | ML+ | D2 | P2 | 1 | 2 | 0 |
| M2 | ML+ | D2 | P2 | 2 | 2 | 0 |
| M2 | ML+ | D2 | P2 | 3 | 2 | 0 |
| M2 | ML+ | D2 | P2 | 4 | 2 | 0 |
| M2 | ML+ | D2 | P3 | 1 | 2 | 0 |
| M2 | ML+ | D2 | P3 | 2 | 2 | 0 |
| M2 | ML+ | D2 | P3 | 3 | 2 | 0 |
| M2 | ML+ | D2 | P3 | 4 | 2 | 0 |
| M2 | ML+ | D2 | P4 | 1 | 2 | 0 |
| M2 | ML+ | D2 | P4 | 2 | 2 | 0 |
| M2 | ML+ | D2 | P4 | 3 | 2 | 0 |
| M2 | ML+ | D2 | P4 | 4 | 2 | 0 |
| M2 | ML+ | D2 | P5 | 1 | 2 | 0 |
| M2 | ML+ | D2 | P5 | 2 | 2 | 0 |
| M2 | ML+ | D2 | P5 | 3 | 2 | 0 |
| M2 | ML+ | D2 | P5 | 4 | 2 | 0 |
| M2 | ML+ | D2 | P6 | 1 | 2 | 0 |
| M2 | ML+ | D2 | P6 | 2 | 2 | 0 |
| M2 | ML+ | D2 | P6 | 3 | 2 | 0 |
| M2 | ML+ | D2 | P6 | 4 | 2 | 0 |
| M2 | ML+ | D2 | P7 | 1 | 2 | 0 |
| M2 | ML+ | D2 | P7 | 2 | 2 | 0 |
| M2 | ML+ | D2 | P7 | 3 | 2 | 0 |
| M2 | ML+ | D2 | P7 | 4 | 2 | 0 |
| M2 | ML+ | D2 | P8 | 1 | 2 | 0 |
| M2 | ML+ | D2 | P8 | 2 | 2 | 0 |

| | | | | | | |
|---|---|---|---|---|---|---|
| M2 | ML+ | D2 | P8 | 3 | 2 | 0 |
| M2 | ML+ | D2 | P8 | 4 | 2 | 0 |
| M2 | ML+ | D2 | P9 | 1 | 2 | 0 |
| M2 | ML+ | D2 | P9 | 2 | 2 | 0 |
| M2 | ML+ | D2 | P9 | 3 | 2 | 0 |
| M2 | ML+ | D2 | P9 | 4 | 2 | 0 |
| M2 | ML+ | D2 | P10 | 1 | 2 | 0 |
| M2 | ML+ | D2 | P10 | 2 | 2 | 0 |
| M2 | ML+ | D2 | P10 | 3 | 2 | 0 |
| M2 | ML+ | D2 | P10 | 4 | 2 | 0 |
| M2 | ML+ | D2 | P11 | 1 | 2 | 0 |
| M2 | ML+ | D2 | P11 | 2 | 2 | 0 |
| M2 | ML+ | D2 | P11 | 3 | 2 | 0 |
| M2 | ML+ | D2 | P11 | 4 | 2 | 0 |
| M2 | ML+ | D2 | P12 | 1 | 2 | 0 |
| M2 | ML+ | D2 | P12 | 2 | 2 | 0 |
| M2 | ML+ | D2 | P12 | 3 | 2 | 0 |
| M2 | ML+ | D2 | P12 | 4 | 2 | 0 |
| M2 | ML+ | D2 | P13 | 1 | 2 | 0 |
| M2 | ML+ | D2 | P13 | 2 | 2 | 0 |
| M2 | ML+ | D2 | P13 | 3 | 2 | 0 |
| M2 | ML+ | D2 | P13 | 4 | 2 | 0 |
| M2 | ML+ | D2 | P14 | 1 | 2 | 0 |
| M2 | ML+ | D2 | P14 | 2 | 2 | 0 |
| M2 | ML+ | D2 | P14 | 3 | 2 | 0 |
| M2 | ML+ | D2 | P14 | 4 | 2 | 0 |
| M2 | ML- | D2 | P1 | 1 | 9 | 0 |
| M2 | ML- | D2 | P1 | 2 | 9 | 0 |
| M2 | ML- | D2 | P1 | 3 | 9 | 0 |
| M2 | ML- | D2 | P1 | 4 | 9 | 0 |
| M2 | ML- | D2 | P2 | 1 | 9 | 0 |
| M2 | ML- | D2 | P2 | 2 | 9 | 0 |
| M2 | ML- | D2 | P2 | 3 | 9 | 0 |
| M2 | ML- | D2 | P2 | 4 | 9 | 0 |
| M2 | ML- | D2 | P3 | 1 | 9 | 0 |
| M2 | ML- | D2 | P3 | 2 | 9 | 0 |
| M2 | ML- | D2 | P3 | 3 | 9 | 0 |
| M2 | ML- | D2 | P3 | 4 | 9 | 0 |
| M2 | ML- | D2 | P4 | 1 | 9 | 0 |
| M2 | ML- | D2 | P4 | 2 | 9 | 0 |
| M2 | ML- | D2 | P4 | 3 | 9 | 0 |
| M2 | ML- | D2 | P4 | 4 | 9 | 0 |
| M2 | ML- | D2 | P5 | 1 | 9 | 0 |
| M2 | ML- | D2 | P5 | 2 | 9 | 0 |
| M2 | ML- | D2 | P5 | 3 | 9 | 0 |
| M2 | ML- | D2 | P5 | 4 | 9 | 0 |
| M2 | ML- | D2 | P6 | 1 | 9 | 0 |
| M2 | ML- | D2 | P6 | 2 | 9 | 0 |
| M2 | ML- | D2 | P6 | 3 | 9 | 0 |
| M2 | ML- | D2 | P6 | 4 | 9 | 0 |

| | | | | | | |
|---|---|---|---|---|---|---|
| M2 | ML- | D2 | P7 | 1 | 9 | 0 |
| M2 | ML- | D2 | P7 | 2 | 9 | 0 |
| M2 | ML- | D2 | P7 | 3 | 9 | 0 |
| M2 | ML- | D2 | P7 | 4 | 9 | 0 |
| M2 | ML- | D2 | P8 | 1 | 9 | 0 |
| M2 | ML- | D2 | P8 | 2 | 9 | 0 |
| M2 | ML- | D2 | P8 | 3 | 9 | 0 |
| M2 | ML- | D2 | P8 | 4 | 9 | 0 |
| M2 | ML- | D2 | P9 | 1 | 9 | 0 |
| M2 | ML- | D2 | P9 | 2 | 9 | 0 |
| M2 | ML- | D2 | P9 | 3 | 9 | 0 |
| M2 | ML- | D2 | P9 | 4 | 9 | 0 |
| M2 | ML- | D2 | P10 | 1 | 9 | 0 |
| M2 | ML- | D2 | P10 | 2 | 9 | 0 |
| M2 | ML- | D2 | P10 | 3 | 9 | 0 |
| M2 | ML- | D2 | P10 | 4 | 9 | 0 |
| M2 | ML- | D2 | P11 | 1 | 9 | 0 |
| M2 | ML- | D2 | P11 | 2 | 9 | 0 |
| M2 | ML- | D2 | P11 | 3 | 9 | 0 |
| M2 | ML- | D2 | P11 | 4 | 9 | 0 |
| M2 | ML- | D2 | P12 | 1 | 9 | 0 |
| M2 | ML- | D2 | P12 | 2 | 9 | 0 |
| M2 | ML- | D2 | P12 | 3 | 9 | 0 |
| M2 | ML- | D2 | P12 | 4 | 9 | 0 |
| M2 | ML- | D2 | P13 | 1 | 9 | 0 |
| M2 | ML- | D2 | P13 | 2 | 9 | 0 |
| M2 | ML- | D2 | P13 | 3 | 9 | 0 |
| M2 | ML- | D2 | P13 | 4 | 9 | 0 |
| M2 | ML- | D2 | P14 | 1 | 9 | 0 |
| M2 | ML- | D2 | P14 | 2 | 9 | 0 |
| M2 | ML- | D2 | P14 | 3 | 9 | 0 |
| M2 | ML- | D2 | P14 | 4 | 9 | 0 |
| M2 | ML+ | D2 | P1 | 1 | 9 | 0 |
| M2 | ML+ | D2 | P1 | 2 | 9 | 0 |
| M2 | ML+ | D2 | P1 | 3 | 9 | 0 |
| M2 | ML+ | D2 | P1 | 4 | 9 | 0 |
| M2 | ML+ | D2 | P2 | 1 | 9 | 0 |
| M2 | ML+ | D2 | P2 | 2 | 9 | 0 |
| M2 | ML+ | D2 | P2 | 3 | 9 | 0 |
| M2 | ML+ | D2 | P2 | 4 | 9 | 0 |
| M2 | ML+ | D2 | P3 | 1 | 9 | 0 |
| M2 | ML+ | D2 | P3 | 2 | 9 | 0 |
| M2 | ML+ | D2 | P3 | 3 | 9 | 0 |
| M2 | ML+ | D2 | P3 | 4 | 9 | 0 |
| M2 | ML+ | D2 | P4 | 1 | 9 | 0 |
| M2 | ML+ | D2 | P4 | 2 | 9 | 0 |
| M2 | ML+ | D2 | P4 | 3 | 9 | 0 |
| M2 | ML+ | D2 | P4 | 4 | 9 | 0 |
| M2 | ML+ | D2 | P5 | 1 | 9 | 0 |
| M2 | ML+ | D2 | P5 | 2 | 9 | 0 |

| | | | | | | |
|---|---|---|---|---|---|---|
| M2 | ML+ | D2 | P5 | 3 | 9 | 0 |
| M2 | ML+ | D2 | P5 | 4 | 9 | 0 |
| M2 | ML+ | D2 | P6 | 1 | 9 | 0 |
| M2 | ML+ | D2 | P6 | 2 | 9 | 0 |
| M2 | ML+ | D2 | P6 | 3 | 9 | 0 |
| M2 | ML+ | D2 | P6 | 4 | 9 | 0 |
| M2 | ML+ | D2 | P7 | 1 | 9 | 0 |
| M2 | ML+ | D2 | P7 | 2 | 9 | 0 |
| M2 | ML+ | D2 | P7 | 3 | 9 | 0 |
| M2 | ML+ | D2 | P7 | 4 | 9 | 0 |
| M2 | ML+ | D2 | P8 | 1 | 9 | 0 |
| M2 | ML+ | D2 | P8 | 2 | 9 | 0 |
| M2 | ML+ | D2 | P8 | 3 | 9 | 0 |
| M2 | ML+ | D2 | P8 | 4 | 9 | 0 |
| M2 | ML+ | D2 | P9 | 1 | 9 | 0 |
| M2 | ML+ | D2 | P9 | 2 | 9 | 0 |
| M2 | ML+ | D2 | P9 | 3 | 9 | 0 |
| M2 | ML+ | D2 | P9 | 4 | 9 | 0 |
| M2 | ML+ | D2 | P10 | 1 | 9 | 0 |
| M2 | ML+ | D2 | P10 | 2 | 9 | 0 |
| M2 | ML+ | D2 | P10 | 3 | 9 | 0 |
| M2 | ML+ | D2 | P10 | 4 | 9 | 0 |
| M2 | ML+ | D2 | P11 | 1 | 9 | 0 |
| M2 | ML+ | D2 | P11 | 2 | 9 | 0 |
| M2 | ML+ | D2 | P11 | 3 | 9 | 0 |
| M2 | ML+ | D2 | P11 | 4 | 9 | 0 |
| M2 | ML+ | D2 | P12 | 1 | 9 | 0 |
| M2 | ML+ | D2 | P12 | 2 | 9 | 0 |
| M2 | ML+ | D2 | P12 | 3 | 9 | 0 |
| M2 | ML+ | D2 | P12 | 4 | 9 | 0 |
| M2 | ML+ | D2 | P13 | 1 | 9 | 0 |
| M2 | ML+ | D2 | P13 | 2 | 9 | 0 |
| M2 | ML+ | D2 | P13 | 3 | 9 | 0 |
| M2 | ML+ | D2 | P13 | 4 | 9 | 0 |
| M2 | ML+ | D2 | P14 | 1 | 9 | 0 |
| M2 | ML+ | D2 | P14 | 2 | 9 | 0 |
| M2 | ML+ | D2 | P14 | 3 | 9 | 0 |
| M2 | ML+ | D2 | P14 | 4 | 9 | 0 |
| M2 | ML- | D2 | P1 | 1 | 16 | 0 |
| M2 | ML- | D2 | P1 | 2 | 16 | 0 |
| M2 | ML- | D2 | P1 | 3 | 16 | 0 |
| M2 | ML- | D2 | P1 | 4 | 16 | 0 |
| M2 | ML- | D2 | P2 | 1 | 16 | 0 |
| M2 | ML- | D2 | P2 | 2 | 16 | 0 |
| M2 | ML- | D2 | P2 | 3 | 16 | 0 |
| M2 | ML- | D2 | P2 | 4 | 16 | 0 |
| M2 | ML- | D2 | P3 | 1 | 16 | 0 |
| M2 | ML- | D2 | P3 | 2 | 16 | 0 |
| M2 | ML- | D2 | P3 | 3 | 16 | 0 |
| M2 | ML- | D2 | P3 | 4 | 16 | 0 |

| | | | | | | |
|---|---|---|---|---|---|---|
| M2 | ML- | D2 | P4 | 1 | 16 | 0 |
| M2 | ML- | D2 | P4 | 2 | 16 | 0 |
| M2 | ML- | D2 | P4 | 3 | 16 | 0 |
| M2 | ML- | D2 | P4 | 4 | 16 | 0 |
| M2 | ML- | D2 | P5 | 1 | 16 | 0 |
| M2 | ML- | D2 | P5 | 2 | 16 | 0 |
| M2 | ML- | D2 | P5 | 3 | 16 | 0 |
| M2 | ML- | D2 | P5 | 4 | 16 | 0 |
| M2 | ML- | D2 | P6 | 1 | 16 | 0 |
| M2 | ML- | D2 | P6 | 2 | 16 | 0 |
| M2 | ML- | D2 | P6 | 3 | 16 | 0 |
| M2 | ML- | D2 | P6 | 4 | 16 | 0 |
| M2 | ML- | D2 | P7 | 1 | 16 | 0 |
| M2 | ML- | D2 | P7 | 2 | 16 | 0 |
| M2 | ML- | D2 | P7 | 3 | 16 | 0 |
| M2 | ML- | D2 | P7 | 4 | 16 | 0 |
| M2 | ML- | D2 | P8 | 1 | 16 | 0 |
| M2 | ML- | D2 | P8 | 2 | 16 | 0 |
| M2 | ML- | D2 | P8 | 3 | 16 | 0 |
| M2 | ML- | D2 | P8 | 4 | 16 | 0 |
| M2 | ML- | D2 | P9 | 1 | 16 | 0 |
| M2 | ML- | D2 | P9 | 2 | 16 | 0 |
| M2 | ML- | D2 | P9 | 3 | 16 | 1 |
| M2 | ML- | D2 | P9 | 4 | 16 | 0 |
| M2 | ML- | D2 | P10 | 1 | 16 | 0 |
| M2 | ML- | D2 | P10 | 2 | 16 | 0 |
| M2 | ML- | D2 | P10 | 3 | 16 | 0 |
| M2 | ML- | D2 | P10 | 4 | 16 | 0 |
| M2 | ML- | D2 | P11 | 1 | 16 | 0 |
| M2 | ML- | D2 | P11 | 2 | 16 | 0 |
| M2 | ML- | D2 | P11 | 3 | 16 | 0 |
| M2 | ML- | D2 | P11 | 4 | 16 | 0 |
| M2 | ML- | D2 | P12 | 1 | 16 | 0 |
| M2 | ML- | D2 | P12 | 2 | 16 | 0 |
| M2 | ML- | D2 | P12 | 3 | 16 | 0 |
| M2 | ML- | D2 | P12 | 4 | 16 | 0 |
| M2 | ML- | D2 | P13 | 1 | 16 | 0 |
| M2 | ML- | D2 | P13 | 2 | 16 | 0 |
| M2 | ML- | D2 | P13 | 3 | 16 | 0 |
| M2 | ML- | D2 | P13 | 4 | 16 | 0 |
| M2 | ML- | D2 | P14 | 1 | 16 | 0 |
| M2 | ML- | D2 | P14 | 2 | 16 | 0 |
| M2 | ML- | D2 | P14 | 3 | 16 | 0 |
| M2 | ML- | D2 | P14 | 4 | 16 | 0 |
| M2 | ML+ | D2 | P1 | 1 | 16 | 0 |
| M2 | ML+ | D2 | P1 | 2 | 16 | 0 |
| M2 | ML+ | D2 | P1 | 3 | 16 | 0 |
| M2 | ML+ | D2 | P1 | 4 | 16 | 0 |
| M2 | ML+ | D2 | P2 | 1 | 16 | 0 |
| M2 | ML+ | D2 | P2 | 2 | 16 | 0 |

| | | | | | | |
|---|---|---|---|---|---|---|
| M2 | ML+ | D2 | P2 | 3 | 16 | 0 |
| M2 | ML+ | D2 | P2 | 4 | 16 | 0 |
| M2 | ML+ | D2 | P3 | 1 | 16 | 0 |
| M2 | ML+ | D2 | P3 | 2 | 16 | 0 |
| M2 | ML+ | D2 | P3 | 3 | 16 | 0 |
| M2 | ML+ | D2 | P3 | 4 | 16 | 0 |
| M2 | ML+ | D2 | P4 | 1 | 16 | 0 |
| M2 | ML+ | D2 | P4 | 2 | 16 | 0 |
| M2 | ML+ | D2 | P4 | 3 | 16 | 0 |
| M2 | ML+ | D2 | P4 | 4 | 16 | 0 |
| M2 | ML+ | D2 | P5 | 1 | 16 | 0 |
| M2 | ML+ | D2 | P5 | 2 | 16 | 0 |
| M2 | ML+ | D2 | P5 | 3 | 16 | 0 |
| M2 | ML+ | D2 | P5 | 4 | 16 | 0 |
| M2 | ML+ | D2 | P6 | 1 | 16 | 0 |
| M2 | ML+ | D2 | P6 | 2 | 16 | 0 |
| M2 | ML+ | D2 | P6 | 3 | 16 | 0 |
| M2 | ML+ | D2 | P6 | 4 | 16 | 0 |
| M2 | ML+ | D2 | P7 | 1 | 16 | 0 |
| M2 | ML+ | D2 | P7 | 2 | 16 | 0 |
| M2 | ML+ | D2 | P7 | 3 | 16 | 0 |
| M2 | ML+ | D2 | P7 | 4 | 16 | 0 |
| M2 | ML+ | D2 | P8 | 1 | 16 | 0 |
| M2 | ML+ | D2 | P8 | 2 | 16 | 0 |
| M2 | ML+ | D2 | P8 | 3 | 16 | 0 |
| M2 | ML+ | D2 | P8 | 4 | 16 | 0 |
| M2 | ML+ | D2 | P9 | 1 | 16 | 0 |
| M2 | ML+ | D2 | P9 | 2 | 16 | 0 |
| M2 | ML+ | D2 | P9 | 3 | 16 | 0 |
| M2 | ML+ | D2 | P9 | 4 | 16 | 0 |
| M2 | ML+ | D2 | P10 | 1 | 16 | 0 |
| M2 | ML+ | D2 | P10 | 2 | 16 | 0 |
| M2 | ML+ | D2 | P10 | 3 | 16 | 0 |
| M2 | ML+ | D2 | P10 | 4 | 16 | 0 |
| M2 | ML+ | D2 | P11 | 1 | 16 | 0 |
| M2 | ML+ | D2 | P11 | 2 | 16 | 0 |
| M2 | ML+ | D2 | P11 | 3 | 16 | 0 |
| M2 | ML+ | D2 | P11 | 4 | 16 | 0 |
| M2 | ML+ | D2 | P12 | 1 | 16 | 0 |
| M2 | ML+ | D2 | P12 | 2 | 16 | 0 |
| M2 | ML+ | D2 | P12 | 3 | 16 | 0 |
| M2 | ML+ | D2 | P12 | 4 | 16 | 0 |
| M2 | ML+ | D2 | P13 | 1 | 16 | 0 |
| M2 | ML+ | D2 | P13 | 2 | 16 | 0 |
| M2 | ML+ | D2 | P13 | 3 | 16 | 0 |
| M2 | ML+ | D2 | P13 | 4 | 16 | 0 |
| M2 | ML+ | D2 | P14 | 1 | 16 | 0 |
| M2 | ML+ | D2 | P14 | 2 | 16 | 0 |
| M2 | ML+ | D2 | P14 | 3 | 16 | 0 |
| M2 | ML+ | D2 | P14 | 4 | 16 | 0 |

</Data>
