# Supplementary material for: Reducing the Use of Pesticides with Site-Specific Application: The Chemical Control of Rhizoctonia solani as a Case of Study for the Management of Soil-Borne Diseases
Source: PLoS One. 2016 Sep 26;11(9):e0163221. doi: 10.1371/journal.pone.0163221 (PMC5036793; doi:10.1371/journal.pone.0163221)
Supplement: S3 File — This pdf file contains raw data used in our analysis to assess the effect of Monceren® L on pathogen infectivity. (PDF) [file pone.0163221.s003.pdf]

<Metadata>
Manip = repetition
Mod = ML+ for Moncern®L sprayed, ML- for water spraying
Pot = Pot
Health = 1 if plant is healthy, otherwise 0
D.Off = 1 if plant is with damping-off, otherwise 0
Nec = 1 if plant is with necrosis, otherwise 0
</Metadata>
<Data>

Manip	Mod	Pot	Health	Nec	DO
1	ML+	1	1	0	1
1	ML+	2	1	0	1
1	ML+	3	1	0	1
1	ML+	4	1	0	1
1	ML+	5	1	0	1
1	ML+	6	1	0	1
1	ML+	7	1	0	1
1	ML+	8	1	0	1
1	ML+	9	1	0	1
1	ML+	10	1	0	1
1	ML+	11	1	0	1
1	ML+	12	1	0	1
1	ML+	13	1	0	1
1	ML+	14	1	0	1
1	ML+	15	1	0	1
1	ML+	16	1	0	1
1	ML+	17	1	0	1
1	ML+	18	1	0	1
1	ML+	19	1	0	1
1	ML+	20	1	0	1
1	ML-	1	1	0	1
1	ML-	2	1	0	1
1	ML-	3	1	0	1
1	ML-	4	1	0	1
1	ML-	5	1	0	1
1	ML-	6	1	0	1
1	ML-	7	0	0	0
1	ML-	8	1	0	1
1	ML-	9	1	1	0
1	ML-	10	1	0	1
1	ML-	11	1	0	1
1	ML-	12	0	0	0
1	ML-	13	1	0	1
1	ML-	14	1	0	1
1	ML-	15	1	0	1
1	ML-	16	1	0	1
1	ML-	17	1	0	1
1	ML-	18	1	0	1
1	ML-	19	1	0	1
1	ML-	20	1	0	1
2	ML+	1	1	0	1
2	ML+	2	1	0	1
2	ML+	3	1	0	1
2	ML+	4	1	0	1
2	ML+	5	1	1	0
2	ML+	6	1	0	1
2	ML+	7	0	0	0
2	ML+	8	1	0	1
2	ML+	9	1	0	1

2	ML+	10	1	0	1
2	ML+	11	1	0	1
2	ML+	12	1	0	1
2	ML+	13	0	0	0
2	ML+	14	1	0	1
2	ML+	15	1	0	1
2	ML+	16	1	0	1
2	ML+	17	0	0	0
2	ML+	18	1	0	1
2	ML+	19	1	0	1
2	ML+	20	1	0	1
2	ML+	21	1	0	1
2	ML-	1	1	0	1
2	ML-	2	1	0	1
2	ML-	3	0	0	0
2	ML-	4	1	1	0
2	ML-	5	1	0	1
2	ML-	6	0	0	0
2	ML-	7	1	0	1
2	ML-	8	1	1	0
2	ML-	9	1	0	1
2	ML-	10	0	0	0
2	ML-	11	1	0	1
2	ML-	12	1	0	1
2	ML-	13	1	0	1
2	ML-	14	0	0	0
2	ML-	15	1	0	1
2	ML-	16	1	0	1
2	ML-	17	1	0	1
2	ML-	18	1	0	1
2	ML-	19	1	0	1
2	ML-	20	1	0	1

</Data>
