# Supplementary material for: Reducing the Use of Pesticides with Site-Specific Application: The Chemical Control of Rhizoctonia solani as a Case of Study for the Management of Soil-Borne Diseases
Source: PLoS One. 2016 Sep 26;11(9):e0163221. doi: 10.1371/journal.pone.0163221 (PMC5036793; doi:10.1371/journal.pone.0163221)
Supplement: S4 File — This pdf file contains raw data used in our analysis to assess the effect of Monceren® L on pathogenic spread. (PDF) [file pone.0163221.s004.pdf]

<Metadata>
Manip = repetition
Mod = ML+ for Moncern®L spraying, ML- for water spraying
Dis = Distance
Pot = Pot
Zone = Zone
Bait = Bait
Time = number of days after inoculation when the baits are placed in the semi-selective medium
Col = 1 if bait is colonized, otherwise = 0
</Metadata>
<Data>

Manip	Mod	Dis	Pot	Bait	Temps	Col
M1	ML-	D2	P1	A	2	0
M1	ML-	D2	P1	B	2	0
M1	ML-	D2	P1	C	2	0
M1	ML-	D2	P1	D	2	0
M1	ML-	D2	P2	A	2	0
M1	ML-	D2	P2	B	2	0
M1	ML-	D2	P2	C	2	0
M1	ML-	D2	P2	D	2	0
M1	ML-	D2	P4	A	2	0
M1	ML-	D2	P4	B	2	0
M1	ML-	D2	P4	C	2	0
M1	ML-	D2	P4	D	2	0
M1	ML-	D2	P5	A	2	0
M1	ML-	D2	P5	B	2	0
M1	ML-	D2	P5	C	2	0
M1	ML-	D2	P5	D	2	0
M1	ML-	D2	P6	A	2	0
M1	ML-	D2	P6	B	2	0
M1	ML-	D2	P6	C	2	0
M1	ML-	D2	P6	D	2	0
M1	ML-	D2	P7	A	2	0
M1	ML-	D2	P7	B	2	0
M1	ML-	D2	P7	C	2	0
M1	ML-	D2	P7	D	2	0
M1	ML-	D2	P8	A	2	0
M1	ML-	D2	P8	B	2	0
M1	ML-	D2	P8	C	2	0
M1	ML-	D2	P8	D	2	0
M1	ML-	D2	P9	A	2	0
M1	ML-	D2	P9	B	2	0
M1	ML-	D2	P9	C	2	0
M1	ML-	D2	P9	D	2	0
M1	ML-	D2	P11	A	2	0
M1	ML-	D2	P11	B	2	0
M1	ML-	D2	P11	C	2	0
M1	ML-	D2	P11	D	2	0
M1	ML-	D2	P12	A	2	0
M1	ML-	D2	P12	B	2	0
M1	ML-	D2	P12	C	2	0
M1	ML-	D2	P12	D	2	0
M1	ML-	D2	P13	A	2	0
M1	ML-	D2	P13	B	2	0
M1	ML-	D2	P13	C	2	0
M1	ML-	D2	P13	D	2	0
M1	ML-	D2	P14	A	2	0
M1	ML-	D2	P14	B	2	0
M1	ML-	D2	P14	C	2	0

M1	ML-	D2	P14	D	2	0
M1	ML+	D2	P2	A	2	0
M1	ML+	D2	P2	B	2	0
M1	ML+	D2	P2	C	2	0
M1	ML+	D2	P2	D	2	0
M1	ML+	D2	P5	A	2	0
M1	ML+	D2	P5	B	2	0
M1	ML+	D2	P5	C	2	0
M1	ML+	D2	P5	D	2	0
M1	ML+	D2	P6	A	2	0
M1	ML+	D2	P6	B	2	0
M1	ML+	D2	P6	C	2	0
M1	ML+	D2	P6	D	2	0
M1	ML+	D2	P7	A	2	0
M1	ML+	D2	P7	B	2	0
M1	ML+	D2	P7	C	2	0
M1	ML+	D2	P7	D	2	0
M1	ML+	D2	P8	A	2	0
M1	ML+	D2	P8	B	2	0
M1	ML+	D2	P8	C	2	0
M1	ML+	D2	P8	D	2	0
M1	ML+	D2	P10	A	2	0
M1	ML+	D2	P10	B	2	0
M1	ML+	D2	P10	C	2	0
M1	ML+	D2	P10	D	2	0
M1	ML+	D2	P12	A	2	0
M1	ML+	D2	P12	B	2	0
M1	ML+	D2	P12	C	2	0
M1	ML+	D2	P12	D	2	0
M1	ML+	D2	P13	A	2	0
M1	ML+	D2	P13	B	2	0
M1	ML+	D2	P13	C	2	0
M1	ML+	D2	P13	D	2	0
M1	ML+	D2	P14	A	2	0
M1	ML+	D2	P14	B	2	0
M1	ML+	D2	P14	C	2	0
M1	ML+	D2	P14	D	2	0
M2	ML-	D2	P1	A	2	0
M2	ML-	D2	P1	B	2	0
M2	ML-	D2	P1	C	2	0
M2	ML-	D2	P1	D	2	0
M2	ML-	D2	P2	A	2	0
M2	ML-	D2	P2	B	2	0
M2	ML-	D2	P2	C	2	0
M2	ML-	D2	P2	D	2	0
M2	ML-	D2	P3	A	2	0
M2	ML-	D2	P3	B	2	0
M2	ML-	D2	P3	C	2	0
M2	ML-	D2	P3	D	2	0
M2	ML-	D2	P5	A	2	0
M2	ML-	D2	P5	B	2	0
M2	ML-	D2	P5	C	2	0
M2	ML-	D2	P5	D	2	0
M2	ML-	D2	P6	A	2	0
M2	ML-	D2	P6	B	2	0
M2	ML-	D2	P6	C	2	0
M2	ML-	D2	P6	D	2	0
M2	ML-	D2	P7	A	2	0
M2	ML-	D2	P7	B	2	0

M2	ML-	D2	P7	C	2	0
M2	ML-	D2	P7	D	2	0
M2	ML-	D2	P8	A	2	0
M2	ML-	D2	P8	B	2	0
M2	ML-	D2	P8	C	2	0
M2	ML-	D2	P8	D	2	0
M2	ML-	D2	P9	A	2	0
M2	ML-	D2	P9	B	2	0
M2	ML-	D2	P9	C	2	0
M2	ML-	D2	P9	D	2	0
M2	ML-	D2	P10	A	2	0
M2	ML-	D2	P10	B	2	0
M2	ML-	D2	P10	C	2	0
M2	ML-	D2	P10	D	2	0
M2	ML-	D2	P12	A	2	0
M2	ML-	D2	P12	B	2	0
M2	ML-	D2	P12	C	2	0
M2	ML-	D2	P12	D	2	0
M2	ML-	D2	P13	A	2	0
M2	ML-	D2	P13	B	2	0
M2	ML-	D2	P13	C	2	0
M2	ML-	D2	P13	D	2	0
M2	ML-	D2	P14	A	2	0
M2	ML-	D2	P14	B	2	0
M2	ML-	D2	P14	C	2	0
M2	ML-	D2	P14	D	2	0
M2	ML+	D2	P1	A	2	0
M2	ML+	D2	P1	B	2	0
M2	ML+	D2	P1	C	2	0
M2	ML+	D2	P1	D	2	0
M2	ML+	D2	P3	A	2	0
M2	ML+	D2	P3	B	2	0
M2	ML+	D2	P3	C	2	0
M2	ML+	D2	P3	D	2	0
M2	ML+	D2	P4	A	2	0
M2	ML+	D2	P4	B	2	0
M2	ML+	D2	P4	C	2	0
M2	ML+	D2	P4	D	2	0
M2	ML+	D2	P5	A	2	0
M2	ML+	D2	P5	B	2	0
M2	ML+	D2	P5	C	2	0
M2	ML+	D2	P5	D	2	0
M2	ML+	D2	P6	A	2	0
M2	ML+	D2	P6	B	2	0
M2	ML+	D2	P6	C	2	0
M2	ML+	D2	P6	D	2	0
M2	ML+	D2	P7	A	2	0
M2	ML+	D2	P7	B	2	0
M2	ML+	D2	P7	C	2	0
M2	ML+	D2	P7	D	2	0
M2	ML+	D2	P9	A	2	0
M2	ML+	D2	P9	B	2	0
M2	ML+	D2	P9	C	2	0
M2	ML+	D2	P9	D	2	0
M2	ML+	D2	P10	A	2	0
M2	ML+	D2	P10	B	2	0
M2	ML+	D2	P10	C	2	0
M2	ML+	D2	P10	D	2	0
M2	ML+	D2	P11	A	2	0

M2	ML+	D2	P11	B	2	0
M2	ML+	D2	P11	C	2	0
M2	ML+	D2	P11	D	2	0
M2	ML+	D2	P12	A	2	0
M2	ML+	D2	P12	B	2	0
M2	ML+	D2	P12	C	2	0
M2	ML+	D2	P12	D	2	0
M1	ML-	D2	P1	A	9	0
M1	ML-	D2	P1	B	9	0
M1	ML-	D2	P1	C	9	0
M1	ML-	D2	P1	D	9	0
M1	ML-	D2	P2	A	9	0
M1	ML-	D2	P2	B	9	0
M1	ML-	D2	P2	C	9	0
M1	ML-	D2	P2	D	9	0
M1	ML-	D2	P4	A	9	0
M1	ML-	D2	P4	B	9	0
M1	ML-	D2	P4	C	9	1
M1	ML-	D2	P4	D	9	1
M1	ML-	D2	P5	A	9	1
M1	ML-	D2	P5	B	9	1
M1	ML-	D2	P5	C	9	1
M1	ML-	D2	P5	D	9	0
M1	ML-	D2	P6	A	9	0
M1	ML-	D2	P6	B	9	0
M1	ML-	D2	P6	C	9	0
M1	ML-	D2	P6	D	9	0
M1	ML-	D2	P7	A	9	1
M1	ML-	D2	P7	B	9	1
M1	ML-	D2	P7	C	9	0
M1	ML-	D2	P7	D	9	0
M1	ML-	D2	P8	A	9	0
M1	ML-	D2	P8	B	9	0
M1	ML-	D2	P8	C	9	0
M1	ML-	D2	P8	D	9	0
M1	ML-	D2	P9	A	9	0
M1	ML-	D2	P9	B	9	1
M1	ML-	D2	P9	C	9	0
M1	ML-	D2	P9	D	9	1
M1	ML-	D2	P11	A	9	0
M1	ML-	D2	P11	B	9	0
M1	ML-	D2	P11	C	9	0
M1	ML-	D2	P11	D	9	1
M1	ML-	D2	P12	A	9	0
M1	ML-	D2	P12	B	9	1
M1	ML-	D2	P12	C	9	0
M1	ML-	D2	P12	D	9	0
M1	ML-	D2	P13	A	9	0
M1	ML-	D2	P13	B	9	0
M1	ML-	D2	P13	C	9	0
M1	ML-	D2	P13	D	9	0
M1	ML-	D2	P14	A	9	0
M1	ML-	D2	P14	B	9	0
M1	ML-	D2	P14	C	9	0
M1	ML-	D2	P14	D	9	0
M1	ML+	D2	P2	A	9	0
M1	ML+	D2	P2	B	9	0
M1	ML+	D2	P2	C	9	0
M1	ML+	D2	P2	D	9	0

M1	ML+	D2	P5	A	9	0
M1	ML+	D2	P5	B	9	0
M1	ML+	D2	P5	C	9	0
M1	ML+	D2	P5	D	9	0
M1	ML+	D2	P6	A	9	1
M1	ML+	D2	P6	B	9	1
M1	ML+	D2	P6	C	9	0
M1	ML+	D2	P6	D	9	0
M1	ML+	D2	P7	A	9	0
M1	ML+	D2	P7	B	9	0
M1	ML+	D2	P7	C	9	0
M1	ML+	D2	P7	D	9	0
M1	ML+	D2	P8	A	9	0
M1	ML+	D2	P8	B	9	0
M1	ML+	D2	P8	C	9	0
M1	ML+	D2	P8	D	9	0
M1	ML+	D2	P10	A	9	0
M1	ML+	D2	P10	B	9	0
M1	ML+	D2	P10	C	9	0
M1	ML+	D2	P10	D	9	0
M1	ML+	D2	P12	A	9	0
M1	ML+	D2	P12	B	9	0
M1	ML+	D2	P12	C	9	0
M1	ML+	D2	P12	D	9	0
M1	ML+	D2	P13	A	9	0
M1	ML+	D2	P13	B	9	0
M1	ML+	D2	P13	C	9	0
M1	ML+	D2	P13	D	9	0
M1	ML+	D2	P14	A	9	0
M1	ML+	D2	P14	B	9	0
M1	ML+	D2	P14	C	9	0
M1	ML+	D2	P14	D	9	0
M2	ML-	D2	P1	A	9	1
M2	ML-	D2	P1	B	9	1
M2	ML-	D2	P1	C	9	0
M2	ML-	D2	P1	D	9	0
M2	ML-	D2	P2	A	9	0
M2	ML-	D2	P2	B	9	0
M2	ML-	D2	P2	C	9	0
M2	ML-	D2	P2	D	9	0
M2	ML-	D2	P3	A	9	1
M2	ML-	D2	P3	B	9	0
M2	ML-	D2	P3	C	9	0
M2	ML-	D2	P3	D	9	0
M2	ML-	D2	P5	A	9	0
M2	ML-	D2	P5	B	9	0
M2	ML-	D2	P5	C	9	0
M2	ML-	D2	P5	D	9	0
M2	ML-	D2	P6	A	9	0
M2	ML-	D2	P6	B	9	1
M2	ML-	D2	P6	C	9	1
M2	ML-	D2	P6	D	9	0
M2	ML-	D2	P7	A	9	1
M2	ML-	D2	P7	B	9	0
M2	ML-	D2	P7	C	9	1
M2	ML-	D2	P7	D	9	1
M2	ML-	D2	P8	A	9	1
M2	ML-	D2	P8	B	9	0
M2	ML-	D2	P8	C	9	0

M2	ML-	D2	P8	D	9	0
M2	ML-	D2	P9	A	9	0
M2	ML-	D2	P9	B	9	0
M2	ML-	D2	P9	C	9	0
M2	ML-	D2	P9	D	9	0
M2	ML-	D2	P10	A	9	1
M2	ML-	D2	P10	B	9	0
M2	ML-	D2	P10	C	9	1
M2	ML-	D2	P10	D	9	1
M2	ML-	D2	P12	A	9	0
M2	ML-	D2	P12	B	9	0
M2	ML-	D2	P12	C	9	0
M2	ML-	D2	P12	D	9	0
M2	ML-	D2	P13	A	9	0
M2	ML-	D2	P13	B	9	0
M2	ML-	D2	P13	C	9	0
M2	ML-	D2	P13	D	9	0
M2	ML-	D2	P14	A	9	0
M2	ML-	D2	P14	B	9	0
M2	ML-	D2	P14	C	9	0
M2	ML-	D2	P14	D	9	1
M2	ML+	D2	P1	A	9	0
M2	ML+	D2	P1	B	9	0
M2	ML+	D2	P1	C	9	1
M2	ML+	D2	P1	D	9	0
M2	ML+	D2	P3	A	9	0
M2	ML+	D2	P3	B	9	0
M2	ML+	D2	P3	C	9	0
M2	ML+	D2	P3	D	9	0
M2	ML+	D2	P4	A	9	0
M2	ML+	D2	P4	B	9	0
M2	ML+	D2	P4	C	9	0
M2	ML+	D2	P4	D	9	0
M2	ML+	D2	P5	A	9	0
M2	ML+	D2	P5	B	9	0
M2	ML+	D2	P5	C	9	0
M2	ML+	D2	P5	D	9	0
M2	ML+	D2	P6	A	9	1
M2	ML+	D2	P6	B	9	1
M2	ML+	D2	P6	C	9	0
M2	ML+	D2	P6	D	9	0
M2	ML+	D2	P7	A	9	0
M2	ML+	D2	P7	B	9	0
M2	ML+	D2	P7	C	9	0
M2	ML+	D2	P7	D	9	0
M2	ML+	D2	P9	A	9	0
M2	ML+	D2	P9	B	9	0
M2	ML+	D2	P9	C	9	0
M2	ML+	D2	P9	D	9	0
M2	ML+	D2	P10	A	9	0
M2	ML+	D2	P10	B	9	0
M2	ML+	D2	P10	C	9	1
M2	ML+	D2	P10	D	9	0
M2	ML+	D2	P11	A	9	0
M2	ML+	D2	P11	B	9	0
M2	ML+	D2	P11	C	9	0
M2	ML+	D2	P11	D	9	0
M2	ML+	D2	P12	A	9	0
M2	ML+	D2	P12	B	9	1

M2	ML+	D2	P12	C	9	0
M2	ML+	D2	P12	D	9	0
M1	ML-	D2	P1	A	16	0
M1	ML-	D2	P1	B	16	0
M1	ML-	D2	P1	C	16	0
M1	ML-	D2	P1	D	16	0
M1	ML-	D2	P2	A	16	0
M1	ML-	D2	P2	B	16	1
M1	ML-	D2	P2	C	16	0
M1	ML-	D2	P2	D	16	0
M1	ML-	D2	P4	A	16	1
M1	ML-	D2	P4	B	16	1
M1	ML-	D2	P4	C	16	1
M1	ML-	D2	P4	D	16	1
M1	ML-	D2	P5	A	16	1
M1	ML-	D2	P5	B	16	1
M1	ML-	D2	P5	C	16	1
M1	ML-	D2	P5	D	16	1
M1	ML-	D2	P6	A	16	1
M1	ML-	D2	P6	B	16	0
M1	ML-	D2	P6	C	16	1
M1	ML-	D2	P6	D	16	0
M1	ML-	D2	P7	A	16	1
M1	ML-	D2	P7	B	16	1
M1	ML-	D2	P7	C	16	1
M1	ML-	D2	P7	D	16	0
M1	ML-	D2	P8	A	16	0
M1	ML-	D2	P8	B	16	0
M1	ML-	D2	P8	C	16	1
M1	ML-	D2	P8	D	16	1
M1	ML-	D2	P9	A	16	0
M1	ML-	D2	P9	B	16	1
M1	ML-	D2	P9	C	16	0
M1	ML-	D2	P9	D	16	1
M1	ML-	D2	P11	A	16	0
M1	ML-	D2	P11	B	16	0
M1	ML-	D2	P11	C	16	0
M1	ML-	D2	P11	D	16	1
M1	ML-	D2	P12	A	16	0
M1	ML-	D2	P12	B	16	1
M1	ML-	D2	P12	C	16	1
M1	ML-	D2	P12	D	16	1
M1	ML-	D2	P13	A	16	0
M1	ML-	D2	P13	B	16	0
M1	ML-	D2	P13	C	16	1
M1	ML-	D2	P13	D	16	0
M1	ML-	D2	P14	A	16	0
M1	ML-	D2	P14	B	16	0
M1	ML-	D2	P14	C	16	1
M1	ML-	D2	P14	D	16	0
M1	ML+	D2	P2	A	16	0
M1	ML+	D2	P2	B	16	0
M1	ML+	D2	P2	C	16	0
M1	ML+	D2	P2	D	16	0
M1	ML+	D2	P5	A	16	0
M1	ML+	D2	P5	B	16	0
M1	ML+	D2	P5	C	16	0
M1	ML+	D2	P5	D	16	0
M1	ML+	D2	P6	A	16	1

M1	ML+	D2	P6	B	16	1
M1	ML+	D2	P6	C	16	0
M1	ML+	D2	P6	D	16	0
M1	ML+	D2	P7	A	16	0
M1	ML+	D2	P7	B	16	0
M1	ML+	D2	P7	C	16	0
M1	ML+	D2	P7	D	16	0
M1	ML+	D2	P8	A	16	0
M1	ML+	D2	P8	B	16	0
M1	ML+	D2	P8	C	16	0
M1	ML+	D2	P8	D	16	1
M1	ML+	D2	P10	A	16	0
M1	ML+	D2	P10	B	16	0
M1	ML+	D2	P10	C	16	0
M1	ML+	D2	P10	D	16	0
M1	ML+	D2	P12	A	16	0
M1	ML+	D2	P12	B	16	0
M1	ML+	D2	P12	C	16	0
M1	ML+	D2	P12	D	16	0
M1	ML+	D2	P13	A	16	1
M1	ML+	D2	P13	B	16	0
M1	ML+	D2	P13	C	16	0
M1	ML+	D2	P13	D	16	0
M1	ML+	D2	P14	A	16	0
M1	ML+	D2	P14	B	16	0
M1	ML+	D2	P14	C	16	0
M1	ML+	D2	P14	D	16	0
M2	ML-	D2	P1	A	16	1
M2	ML-	D2	P1	B	16	1
M2	ML-	D2	P1	C	16	1
M2	ML-	D2	P1	D	16	0
M2	ML-	D2	P2	A	16	0
M2	ML-	D2	P2	B	16	0
M2	ML-	D2	P2	C	16	0
M2	ML-	D2	P2	D	16	0
M2	ML-	D2	P3	A	16	1
M2	ML-	D2	P3	B	16	1
M2	ML-	D2	P3	C	16	0
M2	ML-	D2	P3	D	16	1
M2	ML-	D2	P5	A	16	0
M2	ML-	D2	P5	B	16	1
M2	ML-	D2	P5	C	16	0
M2	ML-	D2	P5	D	16	0
M2	ML-	D2	P6	A	16	1
M2	ML-	D2	P6	B	16	1
M2	ML-	D2	P6	C	16	1
M2	ML-	D2	P6	D	16	1
M2	ML-	D2	P7	A	16	0
M2	ML-	D2	P7	B	16	1
M2	ML-	D2	P7	C	16	1
M2	ML-	D2	P7	D	16	1
M2	ML-	D2	P8	A	16	1
M2	ML-	D2	P8	B	16	1
M2	ML-	D2	P8	C	16	1
M2	ML-	D2	P8	D	16	1
M2	ML-	D2	P9	A	16	0
M2	ML-	D2	P9	B	16	0
M2	ML-	D2	P9	C	16	1
M2	ML-	D2	P9	D	16	1

M2	ML-	D2	P10	A	16	1
M2	ML-	D2	P10	B	16	1
M2	ML-	D2	P10	C	16	1
M2	ML-	D2	P10	D	16	1
M2	ML-	D2	P12	A	16	1
M2	ML-	D2	P12	B	16	1
M2	ML-	D2	P12	C	16	0
M2	ML-	D2	P12	D	16	1
M2	ML-	D2	P13	A	16	0
M2	ML-	D2	P13	B	16	0
M2	ML-	D2	P13	C	16	0
M2	ML-	D2	P13	D	16	0
M2	ML-	D2	P14	A	16	1
M2	ML-	D2	P14	B	16	1
M2	ML-	D2	P14	C	16	1
M2	ML-	D2	P14	D	16	1
M2	ML+	D2	P1	A	16	0
M2	ML+	D2	P1	B	16	0
M2	ML+	D2	P1	C	16	1
M2	ML+	D2	P1	D	16	1
M2	ML+	D2	P3	A	16	0
M2	ML+	D2	P3	B	16	0
M2	ML+	D2	P3	C	16	0
M2	ML+	D2	P3	D	16	0
M2	ML+	D2	P4	A	16	0
M2	ML+	D2	P4	B	16	0
M2	ML+	D2	P4	C	16	0
M2	ML+	D2	P4	D	16	0
M2	ML+	D2	P5	A	16	0
M2	ML+	D2	P5	B	16	0
M2	ML+	D2	P5	C	16	0
M2	ML+	D2	P5	D	16	0
M2	ML+	D2	P6	A	16	0
M2	ML+	D2	P6	B	16	1
M2	ML+	D2	P6	C	16	0
M2	ML+	D2	P6	D	16	0
M2	ML+	D2	P7	A	16	0
M2	ML+	D2	P7	B	16	0
M2	ML+	D2	P7	C	16	0
M2	ML+	D2	P7	D	16	0
M2	ML+	D2	P9	A	16	0
M2	ML+	D2	P9	B	16	0
M2	ML+	D2	P9	C	16	0
M2	ML+	D2	P9	D	16	0
M2	ML+	D2	P10	A	16	0
M2	ML+	D2	P10	B	16	0
M2	ML+	D2	P10	C	16	1
M2	ML+	D2	P10	D	16	1
M2	ML+	D2	P11	A	16	0
M2	ML+	D2	P11	B	16	0
M2	ML+	D2	P11	C	16	0
M2	ML+	D2	P11	D	16	0
M2	ML+	D2	P12	A	16	1
M2	ML+	D2	P12	B	16	0
M2	ML+	D2	P12	C	16	0
M2	ML+	D2	P12	D	16	0
M1	ML-	D5	P2	A	2	0
M1	ML-	D5	P2	B	2	0
M1	ML-	D5	P2	C	2	0

M1	ML-	D5	P2	D	2	0
M1	ML-	D5	P3	A	2	0
M1	ML-	D5	P3	B	2	0
M1	ML-	D5	P3	C	2	0
M1	ML-	D5	P3	D	2	0
M1	ML-	D5	P4	A	2	0
M1	ML-	D5	P4	B	2	0
M1	ML-	D5	P4	C	2	0
M1	ML-	D5	P4	D	2	0
M1	ML-	D5	P5	A	2	0
M1	ML-	D5	P5	B	2	0
M1	ML-	D5	P5	C	2	0
M1	ML-	D5	P5	D	2	0
M1	ML-	D5	P8	A	2	0
M1	ML-	D5	P8	B	2	0
M1	ML-	D5	P8	C	2	0
M1	ML-	D5	P8	D	2	0
M1	ML-	D5	P9	A	2	0
M1	ML-	D5	P9	B	2	0
M1	ML-	D5	P9	C	2	0
M1	ML-	D5	P9	D	2	0
M1	ML-	D5	P10	A	2	0
M1	ML-	D5	P10	B	2	0
M1	ML-	D5	P10	C	2	0
M1	ML-	D5	P10	D	2	0
M1	ML-	D5	P12	A	2	0
M1	ML-	D5	P12	B	2	0
M1	ML-	D5	P12	C	2	0
M1	ML-	D5	P12	D	2	0
M1	ML-	D5	P13	A	2	0
M1	ML-	D5	P13	B	2	0
M1	ML-	D5	P13	C	2	0
M1	ML-	D5	P13	D	2	0
M1	ML-	D5	P14	A	2	0
M1	ML-	D5	P14	B	2	0
M1	ML-	D5	P14	C	2	0
M1	ML-	D5	P14	D	2	0
M1	ML+	D5	P1	A	2	0
M1	ML+	D5	P1	B	2	0
M1	ML+	D5	P1	C	2	0
M1	ML+	D5	P1	D	2	0
M1	ML+	D5	P2	A	2	0
M1	ML+	D5	P2	B	2	0
M1	ML+	D5	P2	C	2	0
M1	ML+	D5	P2	D	2	0
M1	ML+	D5	P5	A	2	0
M1	ML+	D5	P5	B	2	0
M1	ML+	D5	P5	C	2	0
M1	ML+	D5	P5	D	2	0
M1	ML+	D5	P6	A	2	0
M1	ML+	D5	P6	B	2	0
M1	ML+	D5	P6	C	2	0
M1	ML+	D5	P6	D	2	0
M1	ML+	D5	P7	A	2	0
M1	ML+	D5	P7	B	2	0
M1	ML+	D5	P7	C	2	0
M1	ML+	D5	P7	D	2	0
M1	ML+	D5	P8	A	2	0
M1	ML+	D5	P8	B	2	0

M1	ML+	D5	P8	C	2	0
M1	ML+	D5	P8	D	2	0
M1	ML+	D5	P10	A	2	0
M1	ML+	D5	P10	B	2	0
M1	ML+	D5	P10	C	2	0
M1	ML+	D5	P10	D	2	0
M1	ML+	D5	P12	A	2	0
M1	ML+	D5	P12	B	2	0
M1	ML+	D5	P12	C	2	0
M1	ML+	D5	P12	D	2	0
M2	ML-	D5	P1	A	2	0
M2	ML-	D5	P1	B	2	0
M2	ML-	D5	P1	C	2	0
M2	ML-	D5	P1	D	2	0
M2	ML-	D5	P2	A	2	0
M2	ML-	D5	P2	B	2	0
M2	ML-	D5	P2	C	2	0
M2	ML-	D5	P2	D	2	0
M2	ML-	D5	P3	A	2	0
M2	ML-	D5	P3	B	2	0
M2	ML-	D5	P3	C	2	0
M2	ML-	D5	P3	D	2	0
M2	ML-	D5	P4	A	2	0
M2	ML-	D5	P4	B	2	0
M2	ML-	D5	P4	C	2	0
M2	ML-	D5	P4	D	2	0
M2	ML-	D5	P6	A	2	0
M2	ML-	D5	P6	B	2	0
M2	ML-	D5	P6	C	2	0
M2	ML-	D5	P6	D	2	0
M2	ML-	D5	P7	A	2	0
M2	ML-	D5	P7	B	2	0
M2	ML-	D5	P7	C	2	0
M2	ML-	D5	P7	D	2	0
M2	ML-	D5	P8	A	2	0
M2	ML-	D5	P8	B	2	0
M2	ML-	D5	P8	C	2	0
M2	ML-	D5	P8	D	2	0
M2	ML-	D5	P9	A	2	0
M2	ML-	D5	P9	B	2	0
M2	ML-	D5	P9	C	2	0
M2	ML-	D5	P9	D	2	0
M2	ML-	D5	P11	A	2	0
M2	ML-	D5	P11	B	2	0
M2	ML-	D5	P11	C	2	0
M2	ML-	D5	P11	D	2	0
M2	ML-	D5	P12	A	2	0
M2	ML-	D5	P12	B	2	0
M2	ML-	D5	P12	C	2	0
M2	ML-	D5	P12	D	2	0
M2	ML-	D5	P13	A	2	0
M2	ML-	D5	P13	B	2	0
M2	ML-	D5	P13	C	2	0
M2	ML-	D5	P13	D	2	0
M2	ML-	D5	P14	A	2	0
M2	ML-	D5	P14	B	2	0
M2	ML-	D5	P14	C	2	0
M2	ML-	D5	P14	D	2	0
M2	ML+	D5	P1	A	2	0

M2	ML+	D5	P1	B	2	0
M2	ML+	D5	P1	C	2	0
M2	ML+	D5	P1	D	2	0
M2	ML+	D5	P2	A	2	0
M2	ML+	D5	P2	B	2	0
M2	ML+	D5	P2	C	2	0
M2	ML+	D5	P2	D	2	0
M2	ML+	D5	P3	A	2	0
M2	ML+	D5	P3	B	2	0
M2	ML+	D5	P3	C	2	0
M2	ML+	D5	P3	D	2	0
M2	ML+	D5	P4	A	2	0
M2	ML+	D5	P4	B	2	0
M2	ML+	D5	P4	C	2	0
M2	ML+	D5	P4	D	2	0
M2	ML+	D5	P5	A	2	0
M2	ML+	D5	P5	B	2	0
M2	ML+	D5	P5	C	2	0
M2	ML+	D5	P5	D	2	0
M2	ML+	D5	P6	A	2	0
M2	ML+	D5	P6	B	2	0
M2	ML+	D5	P6	C	2	0
M2	ML+	D5	P6	D	2	0
M2	ML+	D5	P7	A	2	0
M2	ML+	D5	P7	B	2	0
M2	ML+	D5	P7	C	2	0
M2	ML+	D5	P7	D	2	0
M2	ML+	D5	P8	A	2	0
M2	ML+	D5	P8	B	2	0
M2	ML+	D5	P8	C	2	0
M2	ML+	D5	P8	D	2	0
M2	ML+	D5	P9	A	2	0
M2	ML+	D5	P9	B	2	0
M2	ML+	D5	P9	C	2	0
M2	ML+	D5	P9	D	2	0
M2	ML+	D5	P10	A	2	0
M2	ML+	D5	P10	B	2	0
M2	ML+	D5	P10	C	2	0
M2	ML+	D5	P10	D	2	0
M2	ML+	D5	P11	A	2	0
M2	ML+	D5	P11	B	2	0
M2	ML+	D5	P11	C	2	0
M2	ML+	D5	P11	D	2	0
M2	ML+	D5	P13	A	2	0
M2	ML+	D5	P13	B	2	0
M2	ML+	D5	P13	C	2	0
M2	ML+	D5	P13	D	2	0
M2	ML+	D5	P14	A	2	0
M2	ML+	D5	P14	B	2	0
M2	ML+	D5	P14	C	2	0
M2	ML+	D5	P14	D	2	0
M1	ML-	D5	P2	A	9	0
M1	ML-	D5	P2	B	9	0
M1	ML-	D5	P2	C	9	0
M1	ML-	D5	P2	D	9	0
M1	ML-	D5	P3	A	9	0
M1	ML-	D5	P3	B	9	0
M1	ML-	D5	P3	C	9	0
M1	ML-	D5	P3	D	9	0

M1	ML-	D5	P4	A	9	0
M1	ML-	D5	P4	B	9	0
M1	ML-	D5	P4	C	9	0
M1	ML-	D5	P4	D	9	0
M1	ML-	D5	P5	A	9	0
M1	ML-	D5	P5	B	9	0
M1	ML-	D5	P5	C	9	0
M1	ML-	D5	P5	D	9	1
M1	ML-	D5	P8	A	9	0
M1	ML-	D5	P8	B	9	0
M1	ML-	D5	P8	C	9	0
M1	ML-	D5	P8	D	9	0
M1	ML-	D5	P9	A	9	0
M1	ML-	D5	P9	B	9	0
M1	ML-	D5	P9	C	9	0
M1	ML-	D5	P9	D	9	0
M1	ML-	D5	P10	A	9	0
M1	ML-	D5	P10	B	9	0
M1	ML-	D5	P10	C	9	0
M1	ML-	D5	P10	D	9	0
M1	ML-	D5	P12	A	9	0
M1	ML-	D5	P12	B	9	0
M1	ML-	D5	P12	C	9	0
M1	ML-	D5	P12	D	9	0
M1	ML-	D5	P13	A	9	0
M1	ML-	D5	P13	B	9	0
M1	ML-	D5	P13	C	9	0
M1	ML-	D5	P13	D	9	0
M1	ML-	D5	P14	A	9	0
M1	ML-	D5	P14	B	9	0
M1	ML-	D5	P14	C	9	0
M1	ML-	D5	P14	D	9	0
M1	ML+	D5	P1	A	9	0
M1	ML+	D5	P1	B	9	0
M1	ML+	D5	P1	C	9	0
M1	ML+	D5	P1	D	9	0
M1	ML+	D5	P2	A	9	0
M1	ML+	D5	P2	B	9	0
M1	ML+	D5	P2	C	9	0
M1	ML+	D5	P2	D	9	0
M1	ML+	D5	P5	A	9	0
M1	ML+	D5	P5	B	9	0
M1	ML+	D5	P5	C	9	0
M1	ML+	D5	P5	D	9	0
M1	ML+	D5	P6	A	9	0
M1	ML+	D5	P6	B	9	0
M1	ML+	D5	P6	C	9	0
M1	ML+	D5	P6	D	9	0
M1	ML+	D5	P7	A	9	0
M1	ML+	D5	P7	B	9	0
M1	ML+	D5	P7	C	9	0
M1	ML+	D5	P7	D	9	0
M1	ML+	D5	P8	A	9	0
M1	ML+	D5	P8	B	9	0
M1	ML+	D5	P8	C	9	0
M1	ML+	D5	P8	D	9	0
M1	ML+	D5	P10	A	9	0
M1	ML+	D5	P10	B	9	0
M1	ML+	D5	P10	C	9	0

M1	ML+	D5	P10	D	9	0
M1	ML+	D5	P12	A	9	0
M1	ML+	D5	P12	B	9	0
M1	ML+	D5	P12	C	9	0
M1	ML+	D5	P12	D	9	0
M2	ML-	D5	P1	A	9	0
M2	ML-	D5	P1	B	9	0
M2	ML-	D5	P1	C	9	0
M2	ML-	D5	P1	D	9	0
M2	ML-	D5	P2	A	9	0
M2	ML-	D5	P2	B	9	0
M2	ML-	D5	P2	C	9	0
M2	ML-	D5	P2	D	9	0
M2	ML-	D5	P3	A	9	0
M2	ML-	D5	P3	B	9	0
M2	ML-	D5	P3	C	9	0
M2	ML-	D5	P3	D	9	0
M2	ML-	D5	P4	A	9	0
M2	ML-	D5	P4	B	9	0
M2	ML-	D5	P4	C	9	0
M2	ML-	D5	P4	D	9	0
M2	ML-	D5	P6	A	9	0
M2	ML-	D5	P6	B	9	0
M2	ML-	D5	P6	C	9	0
M2	ML-	D5	P6	D	9	0
M2	ML-	D5	P7	A	9	0
M2	ML-	D5	P7	B	9	0
M2	ML-	D5	P7	C	9	0
M2	ML-	D5	P7	D	9	0
M2	ML-	D5	P8	A	9	0
M2	ML-	D5	P8	B	9	0
M2	ML-	D5	P8	C	9	0
M2	ML-	D5	P8	D	9	0
M2	ML-	D5	P9	A	9	0
M2	ML-	D5	P9	B	9	0
M2	ML-	D5	P9	C	9	0
M2	ML-	D5	P9	D	9	0
M2	ML-	D5	P11	A	9	0
M2	ML-	D5	P11	B	9	0
M2	ML-	D5	P11	C	9	0
M2	ML-	D5	P11	D	9	0
M2	ML-	D5	P12	A	9	0
M2	ML-	D5	P12	B	9	0
M2	ML-	D5	P12	C	9	1
M2	ML-	D5	P12	D	9	0
M2	ML-	D5	P13	A	9	0
M2	ML-	D5	P13	B	9	0
M2	ML-	D5	P13	C	9	0
M2	ML-	D5	P13	D	9	0
M2	ML-	D5	P14	A	9	0
M2	ML-	D5	P14	B	9	0
M2	ML-	D5	P14	C	9	0
M2	ML-	D5	P14	D	9	0
M2	ML+	D5	P1	A	9	0
M2	ML+	D5	P1	B	9	0
M2	ML+	D5	P1	C	9	0
M2	ML+	D5	P1	D	9	0
M2	ML+	D5	P2	A	9	0
M2	ML+	D5	P2	B	9	0

M2	ML+	D5	P2	C	9	0
M2	ML+	D5	P2	D	9	0
M2	ML+	D5	P3	A	9	0
M2	ML+	D5	P3	B	9	0
M2	ML+	D5	P3	C	9	0
M2	ML+	D5	P3	D	9	0
M2	ML+	D5	P4	A	9	0
M2	ML+	D5	P4	B	9	0
M2	ML+	D5	P4	C	9	0
M2	ML+	D5	P4	D	9	0
M2	ML+	D5	P5	A	9	0
M2	ML+	D5	P5	B	9	0
M2	ML+	D5	P5	C	9	0
M2	ML+	D5	P5	D	9	0
M2	ML+	D5	P6	A	9	0
M2	ML+	D5	P6	B	9	0
M2	ML+	D5	P6	C	9	0
M2	ML+	D5	P6	D	9	0
M2	ML+	D5	P7	A	9	0
M2	ML+	D5	P7	B	9	0
M2	ML+	D5	P7	C	9	0
M2	ML+	D5	P7	D	9	0
M2	ML+	D5	P8	A	9	0
M2	ML+	D5	P8	B	9	0
M2	ML+	D5	P8	C	9	0
M2	ML+	D5	P8	D	9	0
M2	ML+	D5	P9	A	9	0
M2	ML+	D5	P9	B	9	0
M2	ML+	D5	P9	C	9	0
M2	ML+	D5	P9	D	9	0
M2	ML+	D5	P10	A	9	0
M2	ML+	D5	P10	B	9	0
M2	ML+	D5	P10	C	9	0
M2	ML+	D5	P10	D	9	0
M2	ML+	D5	P11	A	9	0
M2	ML+	D5	P11	B	9	0
M2	ML+	D5	P11	C	9	0
M2	ML+	D5	P11	D	9	0
M2	ML+	D5	P13	A	9	0
M2	ML+	D5	P13	B	9	0
M2	ML+	D5	P13	C	9	0
M2	ML+	D5	P13	D	9	0
M2	ML+	D5	P14	A	9	0
M2	ML+	D5	P14	B	9	0
M2	ML+	D5	P14	C	9	0
M2	ML+	D5	P14	D	9	0
M1	ML-	D5	P2	A	16	0
M1	ML-	D5	P2	B	16	0
M1	ML-	D5	P2	C	16	0
M1	ML-	D5	P2	D	16	0
M1	ML-	D5	P3	A	16	0
M1	ML-	D5	P3	B	16	0
M1	ML-	D5	P3	C	16	0
M1	ML-	D5	P3	D	16	0
M1	ML-	D5	P4	A	16	0
M1	ML-	D5	P4	B	16	0
M1	ML-	D5	P4	C	16	0
M1	ML-	D5	P4	D	16	0
M1	ML-	D5	P5	A	16	1

M1	ML-	D5	P5	B	16	1
M1	ML-	D5	P5	C	16	1
M1	ML-	D5	P5	D	16	1
M1	ML-	D5	P8	A	16	0
M1	ML-	D5	P8	B	16	0
M1	ML-	D5	P8	C	16	0
M1	ML-	D5	P8	D	16	0
M1	ML-	D5	P9	A	16	0
M1	ML-	D5	P9	B	16	0
M1	ML-	D5	P9	C	16	0
M1	ML-	D5	P9	D	16	0
M1	ML-	D5	P10	A	16	0
M1	ML-	D5	P10	B	16	0
M1	ML-	D5	P10	C	16	1
M1	ML-	D5	P10	D	16	0
M1	ML-	D5	P12	A	16	0
M1	ML-	D5	P12	B	16	0
M1	ML-	D5	P12	C	16	0
M1	ML-	D5	P12	D	16	0
M1	ML-	D5	P13	A	16	0
M1	ML-	D5	P13	B	16	0
M1	ML-	D5	P13	C	16	1
M1	ML-	D5	P13	D	16	1
M1	ML-	D5	P14	A	16	0
M1	ML-	D5	P14	B	16	0
M1	ML-	D5	P14	C	16	0
M1	ML-	D5	P14	D	16	1
M1	ML+	D5	P1	A	16	0
M1	ML+	D5	P1	B	16	0
M1	ML+	D5	P1	C	16	0
M1	ML+	D5	P1	D	16	0
M1	ML+	D5	P2	A	16	1
M1	ML+	D5	P2	B	16	1
M1	ML+	D5	P2	C	16	0
M1	ML+	D5	P2	D	16	0
M1	ML+	D5	P5	A	16	0
M1	ML+	D5	P5	B	16	0
M1	ML+	D5	P5	C	16	0
M1	ML+	D5	P5	D	16	0
M1	ML+	D5	P6	A	16	1
M1	ML+	D5	P6	B	16	0
M1	ML+	D5	P6	C	16	0
M1	ML+	D5	P6	D	16	0
M1	ML+	D5	P7	A	16	0
M1	ML+	D5	P7	B	16	0
M1	ML+	D5	P7	C	16	0
M1	ML+	D5	P7	D	16	0
M1	ML+	D5	P8	A	16	0
M1	ML+	D5	P8	B	16	0
M1	ML+	D5	P8	C	16	0
M1	ML+	D5	P8	D	16	0
M1	ML+	D5	P10	A	16	0
M1	ML+	D5	P10	B	16	0
M1	ML+	D5	P10	C	16	0
M1	ML+	D5	P10	D	16	0
M1	ML+	D5	P12	A	16	0
M1	ML+	D5	P12	B	16	0
M1	ML+	D5	P12	C	16	0
M1	ML+	D5	P12	D	16	0

M2	ML-	D5	P1	A	16	0
M2	ML-	D5	P1	B	16	0
M2	ML-	D5	P1	C	16	0
M2	ML-	D5	P1	D	16	0
M2	ML-	D5	P2	A	16	0
M2	ML-	D5	P2	B	16	0
M2	ML-	D5	P2	C	16	0
M2	ML-	D5	P2	D	16	0
M2	ML-	D5	P3	A	16	0
M2	ML-	D5	P3	B	16	1
M2	ML-	D5	P3	C	16	0
M2	ML-	D5	P3	D	16	1
M2	ML-	D5	P4	A	16	0
M2	ML-	D5	P4	B	16	0
M2	ML-	D5	P4	C	16	0
M2	ML-	D5	P4	D	16	0
M2	ML-	D5	P6	A	16	0
M2	ML-	D5	P6	B	16	0
M2	ML-	D5	P6	C	16	1
M2	ML-	D5	P6	D	16	0
M2	ML-	D5	P7	A	16	0
M2	ML-	D5	P7	B	16	0
M2	ML-	D5	P7	C	16	0
M2	ML-	D5	P7	D	16	0
M2	ML-	D5	P8	A	16	1
M2	ML-	D5	P8	B	16	1
M2	ML-	D5	P8	C	16	1
M2	ML-	D5	P8	D	16	1
M2	ML-	D5	P9	A	16	1
M2	ML-	D5	P9	B	16	0
M2	ML-	D5	P9	C	16	0
M2	ML-	D5	P9	D	16	0
M2	ML-	D5	P11	A	16	0
M2	ML-	D5	P11	B	16	0
M2	ML-	D5	P11	C	16	0
M2	ML-	D5	P11	D	16	1
M2	ML-	D5	P12	A	16	0
M2	ML-	D5	P12	B	16	1
M2	ML-	D5	P12	C	16	1
M2	ML-	D5	P12	D	16	1
M2	ML-	D5	P13	A	16	1
M2	ML-	D5	P13	B	16	1
M2	ML-	D5	P13	C	16	1
M2	ML-	D5	P13	D	16	1
M2	ML-	D5	P14	A	16	1
M2	ML-	D5	P14	B	16	1
M2	ML-	D5	P14	C	16	1
M2	ML-	D5	P14	D	16	1
M2	ML+	D5	P1	A	16	0
M2	ML+	D5	P1	B	16	0
M2	ML+	D5	P1	C	16	0
M2	ML+	D5	P1	D	16	0
M2	ML+	D5	P2	A	16	0
M2	ML+	D5	P2	B	16	0
M2	ML+	D5	P2	C	16	0
M2	ML+	D5	P2	D	16	0
M2	ML+	D5	P3	A	16	0
M2	ML+	D5	P3	B	16	0
M2	ML+	D5	P3	C	16	0

M2	ML+	D5	P3	D	16	0
M2	ML+	D5	P4	A	16	0
M2	ML+	D5	P4	B	16	0
M2	ML+	D5	P4	C	16	0
M2	ML+	D5	P4	D	16	0
M2	ML+	D5	P5	A	16	0
M2	ML+	D5	P5	B	16	0
M2	ML+	D5	P5	C	16	0
M2	ML+	D5	P5	D	16	0
M2	ML+	D5	P6	A	16	0
M2	ML+	D5	P6	B	16	0
M2	ML+	D5	P6	C	16	0
M2	ML+	D5	P6	D	16	0
M2	ML+	D5	P7	A	16	0
M2	ML+	D5	P7	B	16	0
M2	ML+	D5	P7	C	16	0
M2	ML+	D5	P7	D	16	0
M2	ML+	D5	P8	A	16	0
M2	ML+	D5	P8	B	16	0
M2	ML+	D5	P8	C	16	0
M2	ML+	D5	P8	D	16	0
M2	ML+	D5	P9	A	16	0
M2	ML+	D5	P9	B	16	0
M2	ML+	D5	P9	C	16	0
M2	ML+	D5	P9	D	16	0
M2	ML+	D5	P10	A	16	0
M2	ML+	D5	P10	B	16	0
M2	ML+	D5	P10	C	16	0
M2	ML+	D5	P10	D	16	0
M2	ML+	D5	P11	A	16	0
M2	ML+	D5	P11	B	16	0
M2	ML+	D5	P11	C	16	0
M2	ML+	D5	P11	D	16	0
M2	ML+	D5	P13	A	16	0
M2	ML+	D5	P13	B	16	0
M2	ML+	D5	P13	C	16	0
M2	ML+	D5	P13	D	16	0
M2	ML+	D5	P14	A	16	0
M2	ML+	D5	P14	B	16	0
M2	ML+	D5	P14	C	16	0
M2	ML+	D5	P14	D	16	0
M1	ML-	D7	P2	A	2	0
M1	ML-	D7	P2	B	2	0
M1	ML-	D7	P2	C	2	0
M1	ML-	D7	P2	D	2	0
M1	ML-	D7	P5	A	2	0
M1	ML-	D7	P5	B	2	0
M1	ML-	D7	P5	C	2	0
M1	ML-	D7	P5	D	2	0
M1	ML-	D7	P8	A	2	0
M1	ML-	D7	P8	B	2	0
M1	ML-	D7	P8	C	2	0
M1	ML-	D7	P8	D	2	0
M1	ML-	D7	P9	A	2	0
M1	ML-	D7	P9	B	2	0
M1	ML-	D7	P9	C	2	0
M1	ML-	D7	P9	D	2	0
M1	ML-	D7	P10	A	2	0
M1	ML-	D7	P10	B	2	0

M1	ML-	D7	P10	C	2	0
M1	ML-	D7	P10	D	2	0
M1	ML-	D7	P11	A	2	0
M1	ML-	D7	P11	B	2	0
M1	ML-	D7	P11	C	2	0
M1	ML-	D7	P11	D	2	0
M1	ML-	D7	P12	A	2	0
M1	ML-	D7	P12	B	2	0
M1	ML-	D7	P12	C	2	0
M1	ML-	D7	P12	D	2	0
M1	ML-	D7	P13	A	2	0
M1	ML-	D7	P13	B	2	0
M1	ML-	D7	P13	C	2	0
M1	ML-	D7	P13	D	2	0
M1	ML+	D7	P1	A	2	0
M1	ML+	D7	P1	B	2	0
M1	ML+	D7	P1	C	2	0
M1	ML+	D7	P1	D	2	0
M1	ML+	D7	P2	A	2	0
M1	ML+	D7	P2	B	2	0
M1	ML+	D7	P2	C	2	0
M1	ML+	D7	P2	D	2	0
M1	ML+	D7	P3	A	2	0
M1	ML+	D7	P3	B	2	0
M1	ML+	D7	P3	C	2	0
M1	ML+	D7	P3	D	2	0
M1	ML+	D7	P4	A	2	0
M1	ML+	D7	P4	B	2	0
M1	ML+	D7	P4	C	2	0
M1	ML+	D7	P4	D	2	0
M1	ML+	D7	P5	A	2	0
M1	ML+	D7	P5	B	2	0
M1	ML+	D7	P5	C	2	0
M1	ML+	D7	P5	D	2	0
M1	ML+	D7	P6	A	2	0
M1	ML+	D7	P6	B	2	0
M1	ML+	D7	P6	C	2	0
M1	ML+	D7	P6	D	2	0
M1	ML+	D7	P7	A	2	0
M1	ML+	D7	P7	B	2	0
M1	ML+	D7	P7	C	2	0
M1	ML+	D7	P7	D	2	0
M1	ML+	D7	P8	A	2	0
M1	ML+	D7	P8	B	2	0
M1	ML+	D7	P8	C	2	0
M1	ML+	D7	P8	D	2	0
M1	ML+	D7	P9	A	2	0
M1	ML+	D7	P9	B	2	0
M1	ML+	D7	P9	C	2	0
M1	ML+	D7	P9	D	2	0
M1	ML+	D7	P10	A	2	0
M1	ML+	D7	P10	B	2	0
M1	ML+	D7	P10	C	2	0
M1	ML+	D7	P10	D	2	0
M1	ML+	D7	P11	A	2	0
M1	ML+	D7	P11	B	2	0
M1	ML+	D7	P11	C	2	0
M1	ML+	D7	P11	D	2	0
M1	ML+	D7	P12	A	2	0

M1	ML+	D7	P12	B	2	0
M1	ML+	D7	P12	C	2	0
M1	ML+	D7	P12	D	2	0
M1	ML+	D7	P13	A	2	0
M1	ML+	D7	P13	B	2	0
M1	ML+	D7	P13	C	2	0
M1	ML+	D7	P13	D	2	0
M1	ML+	D7	P14	A	2	0
M1	ML+	D7	P14	B	2	0
M1	ML+	D7	P14	C	2	0
M1	ML+	D7	P14	D	2	0
M2	ML-	D7	P1	A	2	0
M2	ML-	D7	P1	B	2	0
M2	ML-	D7	P1	C	2	0
M2	ML-	D7	P1	D	2	0
M2	ML-	D7	P2	A	2	0
M2	ML-	D7	P2	B	2	0
M2	ML-	D7	P2	C	2	0
M2	ML-	D7	P2	D	2	0
M2	ML-	D7	P4	A	2	0
M2	ML-	D7	P4	B	2	0
M2	ML-	D7	P4	C	2	0
M2	ML-	D7	P4	D	2	0
M2	ML-	D7	P5	A	2	0
M2	ML-	D7	P5	B	2	0
M2	ML-	D7	P5	C	2	0
M2	ML-	D7	P5	D	2	0
M2	ML-	D7	P6	A	2	0
M2	ML-	D7	P6	B	2	0
M2	ML-	D7	P6	C	2	0
M2	ML-	D7	P6	D	2	0
M2	ML-	D7	P7	A	2	0
M2	ML-	D7	P7	B	2	0
M2	ML-	D7	P7	C	2	0
M2	ML-	D7	P7	D	2	0
M2	ML-	D7	P9	A	2	0
M2	ML-	D7	P9	B	2	0
M2	ML-	D7	P9	C	2	0
M2	ML-	D7	P9	D	2	0
M2	ML-	D7	P10	A	2	0
M2	ML-	D7	P10	B	2	0
M2	ML-	D7	P10	C	2	0
M2	ML-	D7	P10	D	2	0
M2	ML-	D7	P11	A	2	0
M2	ML-	D7	P11	B	2	0
M2	ML-	D7	P11	C	2	0
M2	ML-	D7	P11	D	2	0
M2	ML-	D7	P13	A	2	0
M2	ML-	D7	P13	B	2	0
M2	ML-	D7	P13	C	2	0
M2	ML-	D7	P13	D	2	0
M2	ML-	D7	P14	A	2	0
M2	ML-	D7	P14	B	2	0
M2	ML-	D7	P14	C	2	0
M2	ML-	D7	P14	D	2	0
M2	ML+	D7	P2	A	2	0
M2	ML+	D7	P2	B	2	0
M2	ML+	D7	P2	C	2	0
M2	ML+	D7	P2	D	2	0

M2	ML+	D7	P3	A	2	0
M2	ML+	D7	P3	B	2	0
M2	ML+	D7	P3	C	2	0
M2	ML+	D7	P3	D	2	0
M2	ML+	D7	P4	A	2	0
M2	ML+	D7	P4	B	2	0
M2	ML+	D7	P4	C	2	0
M2	ML+	D7	P4	D	2	0
M2	ML+	D7	P5	A	2	0
M2	ML+	D7	P5	B	2	0
M2	ML+	D7	P5	C	2	0
M2	ML+	D7	P5	D	2	0
M2	ML+	D7	P6	A	2	0
M2	ML+	D7	P6	B	2	0
M2	ML+	D7	P6	C	2	0
M2	ML+	D7	P6	D	2	0
M2	ML+	D7	P7	A	2	0
M2	ML+	D7	P7	B	2	0
M2	ML+	D7	P7	C	2	0
M2	ML+	D7	P7	D	2	0
M2	ML+	D7	P11	A	2	0
M2	ML+	D7	P11	B	2	0
M2	ML+	D7	P11	C	2	0
M2	ML+	D7	P11	D	2	0
M2	ML+	D7	P13	A	2	0
M2	ML+	D7	P13	B	2	0
M2	ML+	D7	P13	C	2	0
M2	ML+	D7	P13	D	2	0
M2	ML+	D7	P14	A	2	0
M2	ML+	D7	P14	B	2	0
M2	ML+	D7	P14	C	2	0
M2	ML+	D7	P14	D	2	0
M1	ML-	D7	P2	A	9	0
M1	ML-	D7	P2	B	9	0
M1	ML-	D7	P2	C	9	0
M1	ML-	D7	P2	D	9	0
M1	ML-	D7	P5	A	9	0
M1	ML-	D7	P5	B	9	0
M1	ML-	D7	P5	C	9	0
M1	ML-	D7	P5	D	9	0
M1	ML-	D7	P8	A	9	0
M1	ML-	D7	P8	B	9	0
M1	ML-	D7	P8	C	9	0
M1	ML-	D7	P8	D	9	0
M1	ML-	D7	P9	A	9	0
M1	ML-	D7	P9	B	9	0
M1	ML-	D7	P9	C	9	0
M1	ML-	D7	P9	D	9	0
M1	ML-	D7	P10	A	9	0
M1	ML-	D7	P10	B	9	0
M1	ML-	D7	P10	C	9	0
M1	ML-	D7	P10	D	9	0
M1	ML-	D7	P11	A	9	0
M1	ML-	D7	P11	B	9	0
M1	ML-	D7	P11	C	9	0
M1	ML-	D7	P11	D	9	0
M1	ML-	D7	P12	A	9	0
M1	ML-	D7	P12	B	9	0
M1	ML-	D7	P12	C	9	0

M1	ML-	D7	P12	D	9	0
M1	ML-	D7	P13	A	9	0
M1	ML-	D7	P13	B	9	0
M1	ML-	D7	P13	C	9	0
M1	ML-	D7	P13	D	9	0
M1	ML+	D7	P1	A	9	0
M1	ML+	D7	P1	B	9	0
M1	ML+	D7	P1	C	9	0
M1	ML+	D7	P1	D	9	0
M1	ML+	D7	P2	A	9	0
M1	ML+	D7	P2	B	9	0
M1	ML+	D7	P2	C	9	0
M1	ML+	D7	P2	D	9	0
M1	ML+	D7	P3	A	9	0
M1	ML+	D7	P3	B	9	0
M1	ML+	D7	P3	C	9	0
M1	ML+	D7	P3	D	9	0
M1	ML+	D7	P4	A	9	0
M1	ML+	D7	P4	B	9	0
M1	ML+	D7	P4	C	9	0
M1	ML+	D7	P4	D	9	0
M1	ML+	D7	P5	A	9	0
M1	ML+	D7	P5	B	9	0
M1	ML+	D7	P5	C	9	0
M1	ML+	D7	P5	D	9	0
M1	ML+	D7	P6	A	9	0
M1	ML+	D7	P6	B	9	0
M1	ML+	D7	P6	C	9	0
M1	ML+	D7	P6	D	9	0
M1	ML+	D7	P7	A	9	0
M1	ML+	D7	P7	B	9	0
M1	ML+	D7	P7	C	9	0
M1	ML+	D7	P7	D	9	0
M1	ML+	D7	P8	A	9	0
M1	ML+	D7	P8	B	9	0
M1	ML+	D7	P8	C	9	0
M1	ML+	D7	P8	D	9	0
M1	ML+	D7	P9	A	9	0
M1	ML+	D7	P9	B	9	0
M1	ML+	D7	P9	C	9	0
M1	ML+	D7	P9	D	9	0
M1	ML+	D7	P10	A	9	0
M1	ML+	D7	P10	B	9	0
M1	ML+	D7	P10	C	9	0
M1	ML+	D7	P10	D	9	0
M1	ML+	D7	P11	A	9	0
M1	ML+	D7	P11	B	9	0
M1	ML+	D7	P11	C	9	0
M1	ML+	D7	P11	D	9	0
M1	ML+	D7	P12	A	9	0
M1	ML+	D7	P12	B	9	0
M1	ML+	D7	P12	C	9	0
M1	ML+	D7	P12	D	9	0
M1	ML+	D7	P13	A	9	0
M1	ML+	D7	P13	B	9	0
M1	ML+	D7	P13	C	9	0
M1	ML+	D7	P13	D	9	0
M1	ML+	D7	P14	A	9	0
M1	ML+	D7	P14	B	9	0

M1	ML+	D7	P14	C	9	0
M1	ML+	D7	P14	D	9	0
M2	ML-	D7	P1	A	9	0
M2	ML-	D7	P1	B	9	0
M2	ML-	D7	P1	C	9	0
M2	ML-	D7	P1	D	9	0
M2	ML-	D7	P2	A	9	0
M2	ML-	D7	P2	B	9	0
M2	ML-	D7	P2	C	9	0
M2	ML-	D7	P2	D	9	0
M2	ML-	D7	P4	A	9	0
M2	ML-	D7	P4	B	9	0
M2	ML-	D7	P4	C	9	0
M2	ML-	D7	P4	D	9	0
M2	ML-	D7	P5	A	9	0
M2	ML-	D7	P5	B	9	0
M2	ML-	D7	P5	C	9	0
M2	ML-	D7	P5	D	9	0
M2	ML-	D7	P6	A	9	0
M2	ML-	D7	P6	B	9	0
M2	ML-	D7	P6	C	9	0
M2	ML-	D7	P6	D	9	0
M2	ML-	D7	P7	A	9	0
M2	ML-	D7	P7	B	9	0
M2	ML-	D7	P7	C	9	0
M2	ML-	D7	P7	D	9	0
M2	ML-	D7	P9	A	9	0
M2	ML-	D7	P9	B	9	0
M2	ML-	D7	P9	C	9	0
M2	ML-	D7	P9	D	9	0
M2	ML-	D7	P10	A	9	0
M2	ML-	D7	P10	B	9	0
M2	ML-	D7	P10	C	9	0
M2	ML-	D7	P10	D	9	0
M2	ML-	D7	P11	A	9	0
M2	ML-	D7	P11	B	9	0
M2	ML-	D7	P11	C	9	0
M2	ML-	D7	P11	D	9	0
M2	ML-	D7	P13	A	9	0
M2	ML-	D7	P13	B	9	0
M2	ML-	D7	P13	C	9	0
M2	ML-	D7	P13	D	9	0
M2	ML-	D7	P14	A	9	0
M2	ML-	D7	P14	B	9	0
M2	ML-	D7	P14	C	9	0
M2	ML-	D7	P14	D	9	0
M2	ML+	D7	P2	A	9	0
M2	ML+	D7	P2	B	9	0
M2	ML+	D7	P2	C	9	0
M2	ML+	D7	P2	D	9	0
M2	ML+	D7	P3	A	9	0
M2	ML+	D7	P3	B	9	0
M2	ML+	D7	P3	C	9	0
M2	ML+	D7	P3	D	9	0
M2	ML+	D7	P4	A	9	0
M2	ML+	D7	P4	B	9	0
M2	ML+	D7	P4	C	9	0
M2	ML+	D7	P4	D	9	0
M2	ML+	D7	P5	A	9	0

M2	ML+	D7	P5	B	9	0
M2	ML+	D7	P5	C	9	0
M2	ML+	D7	P5	D	9	0
M2	ML+	D7	P6	A	9	0
M2	ML+	D7	P6	B	9	0
M2	ML+	D7	P6	C	9	0
M2	ML+	D7	P6	D	9	0
M2	ML+	D7	P7	A	9	0
M2	ML+	D7	P7	B	9	0
M2	ML+	D7	P7	C	9	0
M2	ML+	D7	P7	D	9	0
M2	ML+	D7	P11	A	9	0
M2	ML+	D7	P11	B	9	0
M2	ML+	D7	P11	C	9	0
M2	ML+	D7	P11	D	9	0
M2	ML+	D7	P13	A	9	0
M2	ML+	D7	P13	B	9	0
M2	ML+	D7	P13	C	9	0
M2	ML+	D7	P13	D	9	0
M2	ML+	D7	P14	A	9	0
M2	ML+	D7	P14	B	9	0
M2	ML+	D7	P14	C	9	0
M2	ML+	D7	P14	D	9	0
M1	ML-	D7	P2	A	16	0
M1	ML-	D7	P2	B	16	0
M1	ML-	D7	P2	C	16	0
M1	ML-	D7	P2	D	16	0
M1	ML-	D7	P5	A	16	0
M1	ML-	D7	P5	B	16	0
M1	ML-	D7	P5	C	16	0
M1	ML-	D7	P5	D	16	0
M1	ML-	D7	P8	A	16	0
M1	ML-	D7	P8	B	16	0
M1	ML-	D7	P8	C	16	0
M1	ML-	D7	P8	D	16	0
M1	ML-	D7	P9	A	16	0
M1	ML-	D7	P9	B	16	0
M1	ML-	D7	P9	C	16	0
M1	ML-	D7	P9	D	16	0
M1	ML-	D7	P10	A	16	0
M1	ML-	D7	P10	B	16	0
M1	ML-	D7	P10	C	16	0
M1	ML-	D7	P10	D	16	0
M1	ML-	D7	P11	A	16	0
M1	ML-	D7	P11	B	16	0
M1	ML-	D7	P11	C	16	0
M1	ML-	D7	P11	D	16	0
M1	ML-	D7	P12	A	16	0
M1	ML-	D7	P12	B	16	0
M1	ML-	D7	P12	C	16	0
M1	ML-	D7	P12	D	16	0
M1	ML-	D7	P13	A	16	0
M1	ML-	D7	P13	B	16	0
M1	ML-	D7	P13	C	16	0
M1	ML-	D7	P13	D	16	0
M1	ML+	D7	P1	A	16	0
M1	ML+	D7	P1	B	16	0
M1	ML+	D7	P1	C	16	0
M1	ML+	D7	P1	D	16	0

M1	ML+	D7	P2	A	16	0
M1	ML+	D7	P2	B	16	0
M1	ML+	D7	P2	C	16	0
M1	ML+	D7	P2	D	16	0
M1	ML+	D7	P3	A	16	0
M1	ML+	D7	P3	B	16	0
M1	ML+	D7	P3	C	16	0
M1	ML+	D7	P3	D	16	0
M1	ML+	D7	P4	A	16	0
M1	ML+	D7	P4	B	16	0
M1	ML+	D7	P4	C	16	0
M1	ML+	D7	P4	D	16	0
M1	ML+	D7	P5	A	16	0
M1	ML+	D7	P5	B	16	0
M1	ML+	D7	P5	C	16	0
M1	ML+	D7	P5	D	16	0
M1	ML+	D7	P6	A	16	0
M1	ML+	D7	P6	B	16	0
M1	ML+	D7	P6	C	16	0
M1	ML+	D7	P6	D	16	0
M1	ML+	D7	P7	A	16	0
M1	ML+	D7	P7	B	16	0
M1	ML+	D7	P7	C	16	0
M1	ML+	D7	P7	D	16	0
M1	ML+	D7	P8	A	16	0
M1	ML+	D7	P8	B	16	0
M1	ML+	D7	P8	C	16	0
M1	ML+	D7	P8	D	16	0
M1	ML+	D7	P9	A	16	0
M1	ML+	D7	P9	B	16	0
M1	ML+	D7	P9	C	16	0
M1	ML+	D7	P9	D	16	0
M1	ML+	D7	P10	A	16	0
M1	ML+	D7	P10	B	16	0
M1	ML+	D7	P10	C	16	0
M1	ML+	D7	P10	D	16	0
M1	ML+	D7	P11	A	16	0
M1	ML+	D7	P11	B	16	0
M1	ML+	D7	P11	C	16	0
M1	ML+	D7	P11	D	16	0
M1	ML+	D7	P12	A	16	0
M1	ML+	D7	P12	B	16	0
M1	ML+	D7	P12	C	16	0
M1	ML+	D7	P12	D	16	0
M1	ML+	D7	P13	A	16	0
M1	ML+	D7	P13	B	16	0
M1	ML+	D7	P13	C	16	0
M1	ML+	D7	P13	D	16	0
M1	ML+	D7	P14	A	16	0
M1	ML+	D7	P14	B	16	0
M1	ML+	D7	P14	C	16	0
M1	ML+	D7	P14	D	16	0
M2	ML-	D7	P1	A	16	0
M2	ML-	D7	P1	B	16	0
M2	ML-	D7	P1	C	16	0
M2	ML-	D7	P1	D	16	0
M2	ML-	D7	P2	A	16	0
M2	ML-	D7	P2	B	16	0
M2	ML-	D7	P2	C	16	0

M2	ML-	D7	P2	D	16	0
M2	ML-	D7	P4	A	16	0
M2	ML-	D7	P4	B	16	0
M2	ML-	D7	P4	C	16	0
M2	ML-	D7	P4	D	16	0
M2	ML-	D7	P5	A	16	0
M2	ML-	D7	P5	B	16	0
M2	ML-	D7	P5	C	16	0
M2	ML-	D7	P5	D	16	0
M2	ML-	D7	P6	A	16	0
M2	ML-	D7	P6	B	16	0
M2	ML-	D7	P6	C	16	0
M2	ML-	D7	P6	D	16	0
M2	ML-	D7	P7	A	16	0
M2	ML-	D7	P7	B	16	0
M2	ML-	D7	P7	C	16	0
M2	ML-	D7	P7	D	16	0
M2	ML-	D7	P9	A	16	0
M2	ML-	D7	P9	B	16	0
M2	ML-	D7	P9	C	16	0
M2	ML-	D7	P9	D	16	0
M2	ML-	D7	P10	A	16	0
M2	ML-	D7	P10	B	16	1
M2	ML-	D7	P10	C	16	1
M2	ML-	D7	P10	D	16	1
M2	ML-	D7	P11	A	16	0
M2	ML-	D7	P11	B	16	1
M2	ML-	D7	P11	C	16	0
M2	ML-	D7	P11	D	16	1
M2	ML-	D7	P13	A	16	0
M2	ML-	D7	P13	B	16	0
M2	ML-	D7	P13	C	16	0
M2	ML-	D7	P13	D	16	0
M2	ML-	D7	P14	A	16	0
M2	ML-	D7	P14	B	16	0
M2	ML-	D7	P14	C	16	0
M2	ML-	D7	P14	D	16	0
M2	ML+	D7	P2	A	16	0
M2	ML+	D7	P2	B	16	0
M2	ML+	D7	P2	C	16	0
M2	ML+	D7	P2	D	16	0
M2	ML+	D7	P3	A	16	0
M2	ML+	D7	P3	B	16	0
M2	ML+	D7	P3	C	16	0
M2	ML+	D7	P3	D	16	0
M2	ML+	D7	P4	A	16	0
M2	ML+	D7	P4	B	16	0
M2	ML+	D7	P4	C	16	1
M2	ML+	D7	P4	D	16	1
M2	ML+	D7	P5	A	16	0
M2	ML+	D7	P5	B	16	0
M2	ML+	D7	P5	C	16	0
M2	ML+	D7	P5	D	16	0
M2	ML+	D7	P6	A	16	0
M2	ML+	D7	P6	B	16	0
M2	ML+	D7	P6	C	16	0
M2	ML+	D7	P6	D	16	0
M2	ML+	D7	P7	A	16	0
M2	ML+	D7	P7	B	16	0

M2	ML+	D7	P7	C	16	0
M2	ML+	D7	P7	D	16	0
M2	ML+	D7	P11	A	16	0
M2	ML+	D7	P11	B	16	0
M2	ML+	D7	P11	C	16	0
M2	ML+	D7	P11	D	16	0
M2	ML+	D7	P13	A	16	0
M2	ML+	D7	P13	B	16	0
M2	ML+	D7	P13	C	16	0
M2	ML+	D7	P13	D	16	0
M2	ML+	D7	P14	A	16	0
M2	ML+	D7	P14	B	16	0
M2	ML+	D7	P14	C	16	0
M2	ML+	D7	P14	D	16	0

</Data>
